# The structure of the tetraploid sour cherry ‘Schattenmorelle’ (*Prunus cerasus* L.) genome reveals insights into its segmental allopolyploid nature

**DOI:** 10.3389/fpls.2023.1284478

**Published:** 2023-12-01

**Authors:** Thomas W. Wöhner, Ofere F. Emeriewen, Alexander H. J. Wittenberg, Koen Nijbroek, Rui Peng Wang, Evert-Jan Blom, Harrie Schneiders, Jens Keilwagen, Thomas Berner, Katharina J. Hoff, Lars Gabriel, Hannah Thierfeldt, Omar Almolla, Lorenzo Barchi, Mirko Schuster, Janne Lempe, Andreas Peil, Henryk Flachowsky

**Affiliations:** ^1^ Institute for Breeding Research on Fruit Crops, Julius Kühn Institute (JKI) – Federal Research Centre for Cultivated Plants, Dresden, Saxony, Germany; ^2^ KeyGene N.V., Wageningen, Netherlands; ^3^ Institute for Biosafety in Plant Biotechnology, Julius Kühn Institute (JKI) – Federal Research Centre for Cultivated Plants, Quedlinburg, Saxony-Anhalt, Germany; ^4^ Institute of Mathematics and Computer Science, University of Greifswald, Greifswald, Mecklenburg-Western Pomerania, Germany; ^5^ Dipartimento di Scienze Agrarie, Forestali e Alimentari (DISAFA) – Plant Genetics, University of Turin, Grugliasco, Italy

**Keywords:** genome assembly, *P. cerasus*, sour cherry, segmental tetraploid, third generation sequencing, Oxford Nanopore, long reads

## Abstract

Sour cherry (*Prunus cerasus* L.) is an important allotetraploid cherry species that evolved in the Caspian Sea and Black Sea regions from a hybridization of the tetraploid ground cherry (*Prunus fruticosa* Pall.) and an unreduced pollen of the diploid sweet cherry (*P. avium* L.) ancestor. Details of when and where the evolution of this species occurred are unclear, as well as the effect of hybridization on the genome structure. To gain insight, the genome of the sour cherry cultivar ‘Schattenmorelle’ was sequenced using Illumina NovaSeqTM and Oxford Nanopore long-read technologies, resulting in a ~629-Mbp pseudomolecule reference genome. The genome could be separated into two subgenomes, with subgenome *Pce_S_* _a originating from *P. avium* and subgenome *Pce_S_* _f originating from *P. fruticosa*. The genome also showed size reduction compared to ancestral species and traces of homoeologous sequence exchanges throughout. Comparative analysis confirmed that the genome of sour cherry is segmental allotetraploid and evolved very recently in the past.

## 1 Introduction

**Figure 1:**
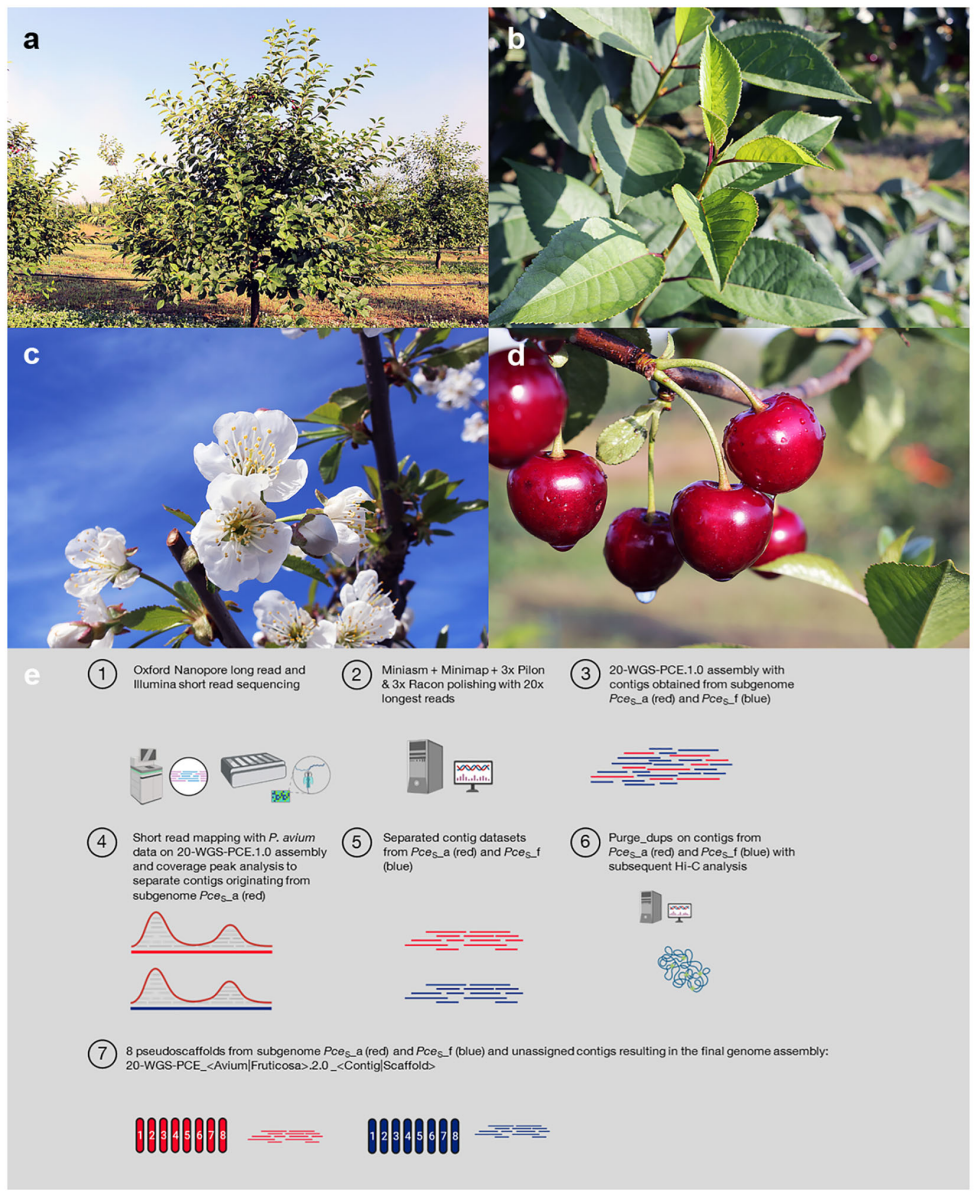
Morphology of *P. cerasus* L. ‘Schattenmorelle’. **(A)** Mature tree habitus, **(B)** leaves, **(C)** inflorescence, **(D)** fruits, **(E)** genome sequencing and assembly strategy (created with BioRender.com).

Cherries include several species of the genus *Prunus*, which belong to the sub-family Spiraeoideae in the plant family Rosaceae (Potter et al., 2007). The two economically most important cherry species worldwide are the sweet cherry (*Prunus avium* L.) and the sour cherry (*Prunus cerasus* L.). Both species are thought to have originated in the Caspian Sea and Black Sea region (Quero-García et al., 2019). Sour cherry commercial cultivation is concentrated in Eastern and Central Europe, North America, and Central and Western Asia, covering 217,960 ha. In 2021, the global production reached 1.51 million tons of fruit, with a production value of $1.2 billion in 2020 (https://www.fao.org/faostat/en/#data). Primarily grown for the production of jams, juices, and preserved or dried whole fruits, they also find use in dairy products and baked goods. Sour cherries display significant variation in morphological traits, including fruit characteristics and tree growth. This diversity is found within ecotypes and includes traits like cold tolerance and growth habits, which have been selectively bred across Europe over time (Dirlewanger et al., 2007; Hancock, 2008; Schuster et al., 2017). However, just a small number of cultivars actually dominate the cultivation of sour cherry. ‘Schattenmorelle’ is the dominant cultivar (cv) in Middle Europe (**Figure 1**), whereas sour cherry production in the United States is still based on Montmorency (Quero-García et al., 2019). ‘Schattenmorelle’ was first described in France and today it is known in many countries with different names. In Poland, for example, it is called Łutovka, and in France, it is called Griotte du Nord or Griotte Noir Tardive. The sour cherry is an allotetraploid with 2n=4x=32 chromosomes. It originated as a hybrid of an unreduced 2n pollen grain of *P*. *avium* (2n=2x=16) and a 1n egg cell of the tetraploid ground cherry *P*. *fruticosa* (2n=4x=32) (Kobel, 1927; Oldén and Nybom, 1968). Evidence of hybridization events between sweet and ground cherries has already been found several times in areas where both species occur simultaneously (Macková et al., 2018; Hrotkó et al., 2020). The resulting hybrids were usually triploid and were assigned to the secondary species *P.* ×*mohacsyana* Kárpáti. Natural occurrences of tetraploid sour cherries can be found in Eastern Turkey and the Caucasus region. There, they grow in forests and are used as wild forms for fruit production. The real area of origin is not known so far. Although *P. cerasus* can also be found in the wild in Europe, it is rather unlikely that those sour cherries are spontaneous hybrids. Since sour cherries are cultivated almost in many areas of the Northern hemisphere, they are often rather allochthonous individuals. The origin of the sour cherry thus seems to be based on a few hybridization events. The results obtained by Oldén and Nybom (1968) in experiments on the resynthesis of the species *P. cerasus* confirmed this hypothesis. The progeny from crosses between sweet and ground cherry showed the characteristic phenotype of the sour cherry. Studies based on chloroplast DNA markers strongly suggest that hybridization between *P. avium* and *P. fruticosa* led to the emergence of *P. cerasus* at least twice (Dirlewanger et al., 2007). Furthermore, the hypothesis could also be confirmed by genomic *in situ* hybridization (Schuster and Schreibner, 2000) and transcriptome sequencing (Bird et al., 2022).

The sour cherry genome is estimated at 599 Mbp (Dirlewanger et al., 2007) with two subgenomes, each featuring eight chromosomes in the haploid set. One subgenome originates from sweet cherry (*Pce*_a), and the other from ground cherry (*Pce*_f). Nonetheless, the genome is not entirely allopolyploid, as there is long-standing suspicion that segments of the sour cherry genome are of different origin (Raptopoulos, 1941; Oldén and Nybom, 1968; Beaver and Iezzoni, 1993; Schuster and Wolfram, 2008). The origins and stability of hybridization and polyploidization between sweet and ground cherry remain unexplored. Cai et al. (2018) suggest that a combination of multi- and bivalent pairing may have led to chromosome segregation imbalance in sour cherry, indicating an ongoing genome stabilization process (Mason and Wendel, 2020). Recent genome sequencing advances, including studies by Zhang et al. (2021), Edger et al. (2019); Bertioli et al. (2019), Wu et al. (2021), and Wang et al. (2019), provide insight into the intricate structure and evolution of polyploid genomes. We present a high-quality pseudo-chromosome-level genome assembly of tetraploid sour cherry ‘Schattenmorelle’ (referred to as *Pce*_S_), created using a combination of Illumina NovaSeq short-read and Oxford Nanopore long-read sequencing. Hi-C technology was employed to scaffold the sequences into chromosomes. Additionally, we generated a full-length transcriptome of ‘Schattenmorelle’ with PacBio Sequel II SMRT cell long-read technology. Comparative sequence and amino acid analyses were conducted across datasets of *Prunus avium* cv Tieton (*Pa*_T_) and *Prunus fruticosa* ecotype Hármashatárhegy (*Pf*_eH_), representing the two ancestral species, alongside the two subgenomes of ‘Schattenmorelle’ (*Pce*_S__a and *Pce*_S__f). These analyses shed light on the evolution of sour cherry and reveal homoeologous exchanges (HE) within the sub-genomic structure, explaining the segmental allopolyploidy in the sour cherry genome.

## 2 Materials and methods

### 2.1 Plant material, DNA and RNA extraction, sequencing and Iso-Seq analysis

Snap-frozen *Prunus cerasus* L. ‘Schattenmorelle’ (accession KIZC99-2, **Figure 1**, supplements 1.1) young leaf material was sent to KeyGene N.V. (Wageningen, The Netherlands). High-molecular-weight extracted DNA (Wöhner et al., 2021) was used to generate 1D ligation (SQK-LSK109) libraries that were subsequently sequenced on two Oxford Nanopore Technologies (ONT) R9.4.1 PromethION flow cells. The same material was used to generate an Illumina PCR free paired-end library (insert size of ~550 bp) that was sequenced on a Illumina NovaSeq™ platform using 150-bp and 125-bp paired-end sequencing.

Snap-frozen tissues from buds, flowers, leaves, and fruits were collected in the field, and total RNA was extracted with Maxwell^®^ RSC Plant RNA Kit (Promega). Two pools were generated and used for PacBio Iso-Seq library preparation (Procedure & Checklist – Iso-Seq™ Express Template Preparation for Sequel^®^ and Sequel II Systems, PN 101-763-800 Version 02). Each library pool was sequenced on a single 8M ZMW PacBio Sequel II SMRT cell (supplements 1.1). Obtained full-length reads with the 5′-end primer, the 3′-end primer, and the poly-A tail were filtered, and these sequences were trimmed off. Transcripts containing (artificial) concatemers were completely discarded. Isoforms (consensus sequence) generated by full-length read clustering (based on sequence similarity) were finally polished with non-full-length reads using Arrow (SMRT Link v7.0.0, https://www.pacb.com/wp-content/uploads/SMRT_Tools_Reference_Guide_v600.pdf).

### 2.2 *De novo* assembly and scaffolding

The aligner Minimap2 (v2.16-r922, Li, 2018) and assembler Miniasm (v0.2-r137-dirty, Li, 2016) were used for raw data assembly generation. Racon (vv1.4.10, Vaser et al., 2017) and Pilon (v1.22, Walker et al., 2014) were used for base-quality improvement with raw ONT and Illumina read data. Chromosome-scale scaffolding was performed by Phase Genomics (Seattle, Washington, USA) with Proximo Hi-C (supplements 1.2). The resulting assembly was designated as 20-WGS-PCE_<Avium|Fruticosa>.2.0 _<Contig|Scaffold> (**Figure 1E**).

### 2.3 Correctness, completeness, and contiguity of the *Prunus cerasus* genome sequence

The BUSCO (Benchmark Universal Single-Copy Orthologs – Galaxy Version 4.1.4) software was used for quantitative and quality assessment of the genome assemblies based on near-universal single-copy orthologs. The long terminal repeat (LTR) assembly Index (LAI) (Ou et al., 2018) was calculated with LTR_retriever 2.9.0 (https://github.com/oushujun/LTR_retriever) to evaluate the assembly continuity between the final genome sequence of *P. cerasus* ‘Schattenmorelle’ and *Prunus fruticosa* ecotype Hármashatárhegy (Wöhner et al., 2021, Pf_1.0), *P. avium* Tieton (Wang et al., 2020), and *P. persica* Lovell (Verde et al., 2017), respectively. LTR_harvest (genometools 1.6.1 implementation) was used to obtain LTR-RT candidates. The genome size was also estimated by k-mer analysis (supplements 1.3) using Illumina short read data. The merged datasets were subsequently used to generate a histogram dataset representing the k-mers from all datasets. GenomeScope (Galaxy Version 2.0, Ranallo-Benavidez et al., 2020) was used to generate a histogram plot of k-mer frequency of different coverage depths using the tetraploid ploidy level (k-mer length 19). Marker sequences and genetic positions from five available genetic sour cherry maps (M172x25-F1, US-F1, 25x25-F1, Montx25-F1, and RE-F1) and 14,644 SNP markers (9 + 6k array) were downloaded from the Genome Database for Rosaceae (GDR, https://www.rosaceae.org/). The marker sequences were mapped on the chromosome sequences using the mapping software bowtie2 (Galaxy Version 2.5.0+galaxy0, Langmead and Salzberg, 2012) implementation on the Galaxy server (https://usegalaxy.org) with standard settings.

### 2.4 Structural and functional annotation

For an interspecies repeat comparison, a species-specific repeat library was generated with RepeatModeler open-1.0.11, and the genome was subsequently masked with RepeatMasker open-4.0.7. For structural genome annotation, another species-specific repeat library for PCE_1.0 was generated with RepeatModeler2 (Flynn et al., 2020) version 2.0.2, and the genome was subsequently masked with RepeatMasker 4.1.2. (Further details on the repeat masking software configuration are available in supplements 1.4.1.).

To generate extrinsic evidence for structural annotation of protein coding genes, short-read RNA-Seq library SRR2290965 (Jo et al., 2015) was aligned to the genome using HiSat2 version 2.1.0 (Kim et al., 2019). The output SAM file was converted to BAM format using SAMtools (Li et al., 2009). The resulting alignment file was further used by both BRAKER1 (Hoff et al., 2016; Hoff et al., 2019) and GeMoMa (Keilwagen et al., 2016; Keilwagen et al., 2019).

Furthermore, a custom protocol was used for integrating long-read RNA-Seq data into genome annotation (supplements 1.4.2). In short, protein coding genes were called in Cupcake transcripts using GeneMarkS-T (Tang et al., 2015), and these predictions were converted to hints for BRAKER1. In addition, intron coverage information from long-read to genome-spliced alignment with Minimap2 (Li, 2018) was provided to BRAKER1.

A combination of BRAKER1 (Hoff et al., 2016; Hoff et al., 2019), BRAKER2 (Brůna et al., 2021), TSEBRA (Gabriel et al., 2021), and GeMoMa (Keilwagen et al., 2016) was used for the final annotation of protein coding genes. BRAKER pipelines use a combination of evidence-supported self-training GeneMark-ET/EP (Lomsadze et al., 2014; Brůna et al., 2020) (here version 4.68) to generate a training gene set for the gene prediction tool AUGUSTUS (Stanke et al., 2008; here version 3.3.2). BRAKER1 version 2.1.6 was here provided with BAM-files of from short- and long-read RNA-Seq to genome alignments, and with gene structure information derived from Cupcake transcripts using GeneMarkS-T. This generated a gene set that consists of *ab initio* and evidence-supported predictions. A separate gene set was generated with BRAKER2, which uses protein to generate a gene set. We used the OrthoDB version 10 (Kriventseva et al., 2019) partition of plants in combination with the full protein sets of *Prunus fruticosa* (Wöhner et al., 2021), *Prunus armeniaca* (GCA 903112645.1)*, Prunus avium* (GCF_002207925.1)*, Prunus dulcis* (GCF_902201215.1)*, Prunus mume* (GCF_000346735.1), and *Prunus persica* (GCF_000346465.2) as input for BRAKER2. Both the BRAKER1 and BRAKER2 AUGUSTUS gene sets were combined with a GeneMarkS-T derived gene set using TSEBRA (Gabriel et al., 2021) from the long_reads branch on GitHub with a custom configuration file (supplements 1.4.3.) incorporating evidence from BRAKER1 and BRAKER2.

GeMoMa was run on the genome assembly of ‘Schattenmorelle’ using 14 reference species and experimental transcript evidence (supplements 1.4.4). GeMoMa gene predictions of each reference species were combined with TSEBRA predictions using the GeMoMa module GAF, and subsequently, UTRs were predicted in a two-step process based on mapped Iso-Seq and RNA-seq data using the GeMoMa module AnnotationFinalizer (supplements 1.4.5). First, UTRs were predicted based on Iso-Seq data. Second, UTRs were predicted based on RNA-seq data for gene predictions without UTR prediction from the first step. An assembly hub for visualization of the *Prunus cerasus* genome with structural annotation was generated using MakeHub (Hoff, 2019; supplements 2). The functional annotation was performed with the Galaxy Europe implementation of InterProScan (Galaxy Version 5.59-91.0+galaxy3, Zdobnov and Apweiler, 2001; Quevillon et al., 2005; Hunter et al., 2009; Cock et al., 2013; Jones et al., 2014). The chloroplast and mitochondria sequences were annotated with GeSeq (Tillich et al., 2017, supplements 1.4.5).

### 2.5 Identification of syntenic regions

Structural comparison of orthologous loci between the subgenomes *Pce*_S__a and *Pce*_S__f of *Prunus cerasus* and the two genotypes *Pa*_T_ and *Pf*_eH_ as representatives of the two genome donor species *P. avium* and *P. fruticosa* was calculated with the final annotations using SynMap2 (Haug-Baltzell et al., 2017) available at the CoGe platform (https://genomevolution.org/coge/). Analysis on triplication events was performed with standard settings and Last as Blast algorithm at a ratio coverage depth of 3:3 in SynMap2 (Haug-Baltzell et al., 2017).

### 2.6 Identification of homoeologous exchange regions

Homoeologous exchanges were identified on the amino acid, transcript, and genomic level.

#### 2.6.1 Calculation of amino acid identity

Identity of amino acids (IAA) between all reference annotation homology-based gene prediction was calculated by GeMoMa using the default parameters. Subsequently, the *Pce*_S_ genome was divided into 250k windows, and the percentage of proteins showing a higher IAA between *Pf_eH_* (Wöhner et al., 2021) and *Pa_T_* (Wang et al., 2020) to the respective subgenome (*Pce*_S__a and *Pce*_S__f) was determined. The percentage of proteins in this window, which were more similar to *Pa*_T_, was finally subtracted from the percentage of proteins that were more similar to *Pf*_eH_. A proportion of transcripts with higher intraspecific amino acid identity (between *Pa_T_* and *Pce_S_* _a or *Pf_eH_* and *Pce_S_* _f) is expected compared to the proportion of transcripts with interspecific amino acid identity match (*Pf_eH_* and *Pce_S_* _a or *Pa_T_* and *Pce_S_* _f). Opposite cases indicate potential translocations between the two subgenomes *Pce*_S__a and *Pce*_S__f and were plotted into a circos plot (**Figure S1**).

#### 2.6.2 Read mapping and coverage analysis

RNAseq raw data published by Bird et al. (2022) were obtained from NCBI sequence read archive (SRA) for the following species: *P. cerasus* (SRX14816146, SRX14816142, and SRX14816138), *P. fruticosa* (SRX14816141), *P. avium* (SRX14816143), *P. canescens* (SRX14816137), *P. serrulata* (SRX14816136), *P. mahaleb* (SRX14816140), *P. pensylvanica* (SRX14816144), *P. maackii* (SRX14816139), and *P. subhirtella* (SRX14816145). Reads were adapter and quality trimmed using the software Trim Galore (version 0.6.3, parameters –quality 30 –length 50). Trimmed reads were mapped against the *P. cerasus* subgenomes *Pce*_S__a and *Pce*_S__f using STAR (version 2.7.8a, parameter –twopassMode Basic). The subsequent analysis was performed in accordance to Keilwagen et al. (2022). The *Pce*_S_ genome was divided into 250k windows. The percentage of covered bases using RNAseq data of *P. cerasus* (SRX14816146, SRX14816142, and SRX14816138) was estimated at a depth of 1 for each window. The same was done with all other RNAseq datasets. The percentage of covered bases from *P. avium* (SRX14816143) was subtracted from the percentage of covered bases from *P. cerasus* (SRX14816146, SRX14816142, and SRX14816138). The same was done using the reads of *P. fruticosa* (SRX14816141). For subgenome *Pce*_S__a, it is expected that the intraspecific difference for transcripts of dataset *P. avium* (SRX14816143) is lower (close to 0) than the interspecific difference for transcripts of dataset *P. fruticosa* (SRX14816141) and *vice versa*. Opposite cases indicate potential homoeologous exchanges between the two subgenomes *Pce*_S__a and *Pce*_S__f and were plotted into a circos plot (**Figure S1**).

The nucleotide short reads from *Pce_S_* were mapped against the genomes of the two ancestral species *Pa*_T_ and *Pf*_eH._ Subsequently, the mapped reads were filtered using samtools for mapped reads in proper pair (-f 3) and primary alignments and not supplementary alignment (-F 2304). Those reads were divided into four groups according to the following criteria: (1) unique match to *Pa*_T_, (2) unique match to *Pf*_eH_, (3) match to *Pa*_T_ and *Pf*_eH_, and (4) no match to *Pa*_T_ and *Pf*_eH_ (unique to *Pce_S_*). The first two separated read sets were then re-mapped against the subgenomes *Pce*_S__a and *Pce*_S__f. The percentage of covered bases was calculated for a 100k window. For the subgenomes of *Pce*_s_, the percentage of intraspecific covered bases (*Pce_S_* _a to *Pa_T_*, *Pce_S_* _f to *Pf_eH_*) should be higher compared to the percentage of interspecific covered bases (*Pce_S_* _a to *Pf_eH_*, *Pce_S_* _f to *Pa_T_*). The opposite case indicates possible translocations and were plotted into a circos plot (**Figure S1**). Additionally, regions of the ‘Schattenmorelle’ genome assembly were determined that are uniquely covered by *Pa*_T_ and *Pf*_eH_ filtered read sets.

### 2.7 LTR insertion estimation

The difference (identity) of left and right LTR was calculated using the script EDTA_raw.pl from the software EDTA version 1.9 (https://github.com/oushujun/EDTA, Ou et al., 2019). As input files, we used the genome sequences of *P. cerasus* (*Pce*_S__a and *Pce*_S__f), *Pa*_T_ (NCBI BioProject acc. no. PRJNA596862), *Pf*_eH_ (NCBI BioProject acc. no. PRJNA727075), and a curated library of representative transposable elements from *Viridiplantae* (https://www.girinst.org/repbase/). Because trees are not annual plants, the identity obtained from the resulting.pass.list file was used for the estimation of generation time after LTR insertion using the formula T=K/2µ (K is the divergence of the LTR = 1 − identity) assuming a *Prunus-*specific mutation frequency of µ=7.7 × 10^−9^ (Xie et al., 2016) per generation.

### 2.8 Protein clustering, multiple sequence alignment, and divergence of time estimation

The protein datasets from *Pce*_S_ *_a* and *Pce*_S_ *_f, Pa*_T_ *, Pf*_eH_ *, Pp* (*Prunus persica* Whole Genome Assembly v2.0, v2.0.a1)*, Pm* (*Prunus mume* Tortuosa Genome v1.0)*, Py* (*Prunus yedoensis* var. *nudiflora* Genome v1.0)*, Md* (*Malus* x *domestica* HFTH1 Whole Genome v1.0), and *At* (TAIR10.1, RefSeq GCF_000001735.4) from the annotation step were uploaded to Galaxy_Europe server as.fasta. The Proteinortho (Galaxy Version 6.0.32+galaxy0) was used to find orthologous proteins within the datasets. MAFFT (Galaxy Version 7.505+galaxy0) was used to align the obtained single copy orthogroups. The final alignments were merged with the Merge.files function (Galaxy Version 1.39.5.0). Finally, the alignments were concatenated into a super protein and the final sequences were aligned with MAFFT. A phylogenetic tree was reconstructed with RAxML (maximum likelihood based inference of large phylogenetic trees, Galaxy Version 8.2.4+galaxy3) and the obtained.nhx file was reformatted as.nwk file for further processing using CLC Mainworkbench (21.0.1, QIAGEN Aarhus A/S). Evolutionary analyses were conducted in MEGA X (Kumar et al., 2018). Estimation of pairwise divergence time was performed according to Shirasawa et al. (2019) with a divergence time from the reference species peach and apple (34–67 Mya, www.timetree.org). Specific parameters for the calculation are listed in supplements.

## 3 Results

### 3.1 *De novo* assembly and scaffolding

**Figure 2:**
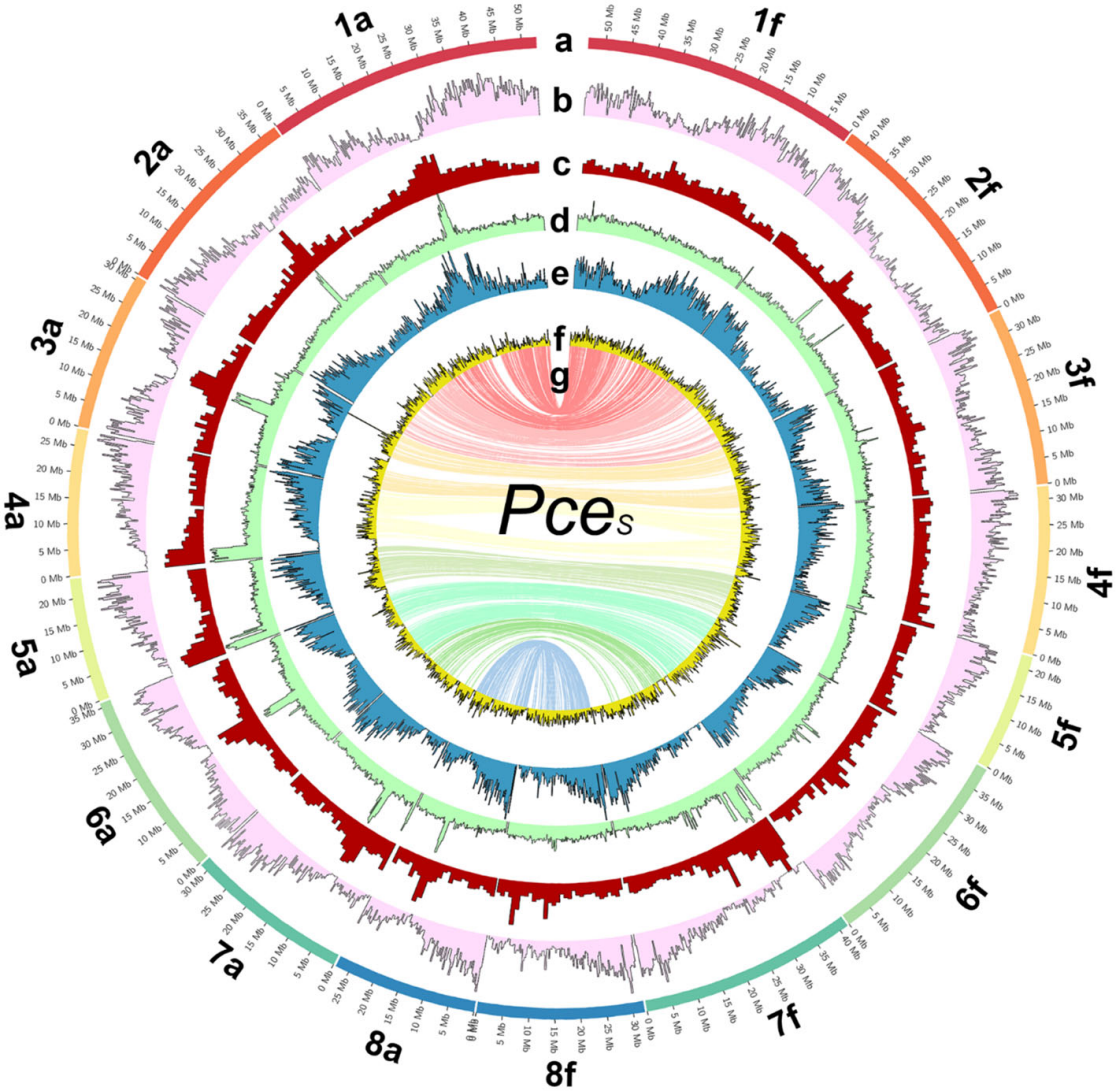
The genome of *P. cerasus* ‘Schattenmorelle’. Circos plot of 16 pseudomolecules of the subgenomes of *Pce_S_* _a and *Pce_S_* _f. **(A)** Chromosome length (Mb). **(B)** Gene density in blocks of 250k. **(C)** Distribution of repetitive sequences in blocks of 250k. **(D)** Gypsy elements in blocks of 250k. **(E)** Copia elements in block of 250k. **(F)** GC content in blocks of 1 Mb. **(G)** The inner ring shows markers from the 6 + 9k SNP array located on both subgenomes.

A total of 68 Gb of paired-end Illumina sequencing data were obtained, corresponding to ~114× coverage of the estimated genome size of 599 Mbp. Using two PromethION flow cells, a total of 178 Gb was produced (~300× coverage). The longest ONT reads that together resulted in a 20× coverage were selected for assembly, having a minimum read length of 64,214 bp. **Table S1** summarizes the properties of the 20-WGS-PCE.1.0 assembly after polishing. The *Prunus avium* and *Prunus fruticosa* contigs were then separated successfully by read mapping and contig selection that fit the hypothesis of 1 or more clear coverage peaks from the 20-WGS-PCE.1.0 assembly. The resulting two datasets, representing the subgenomes *Pce*_S__a and *Pce*_S__f, were purged and used for HI-C scaffolding. After manual curation of both datasets, the final consensus genome assembly was scaffolded from 935 and 865 contigs of the *Pce*_S__a and *Pce*_S__f subgenomes, respectively. Eight clusters ideally representing the eight chromosomes were obtained for each subgenome (**Figure S2**). The final genome sequence is 628.5 Mbp long and consists of eight chromosomes for each subgenome (**Figure 2**). A total of 269 Mbp were assigned to subgenome *Pce*_S__a (N50 of 31.5 Mbp) and 299.5 Mbp (N50 of 39.4 Mbp) to *Pce*_S__f. Eighty-six and 134 unassembled contigs were unassigned to chromosomes for *Pce*_S__a (22.7 Mbp) and *Pce*_S__f (37.3 Mbp), respectively. The longest scaffold from *Pce*_S__a is 52.8 Mbp and 53.5 Mbp from subgenome *Pce*_S__f (**Table S2**). Except for chromosome five, all scaffolds obtained from subgenome *Pce*_S__f are longer compared to the corresponding chromosome of subgenome *Pce*_S__a. The chloroplast sequence obtained was 158,178 bp and the mitochondrial sequence was 343,516 bp long (**Figure S3**).

### 3.2 Transcriptome sequencing, Iso-Seq analysis, structural and functional annotation

The total repeat content of the entire *Pce*_S_ genome sequence was 49.7%. The total repeat content of subgenome *Pce*_S__a was 48.3% and that of subgenome *Pce*_S__f was 50.9% (**Table 1**). The class I elements Gypsy comprised the largest fractions of repetitive elements in the *Pce*_S_ genome sequence. A quantitative reduction between elements of this family was also detected in the *Pce*_S__f subgenome with a difference of 10.7%. Several elements could only be detected in one genotype of the two ancestral species. The TAD1 class I element only occurred in *Pf*_eH_, while class II, order TIR - IS3EU, P, and Sola-3 were specifically detected in the genome of *Pa*_T_. No element was found, which was only present in one of the two subgenomes of *Pce*_S_. Several elements occurred in both subgenomes (class I, LINE – R1-LOA, RTE-X, SINE – tRNA-DEU- L2, class II, TIR – TcMar- Mariner, and DADA elements) but were not detected in *Pf*_eH_ and *Pa*_T_. The class I elements of the order LTR (ERV1, Pao) and Academ/-2 were only detected in one of the two genomes representing the ancestral species and in *Pce*_S__a and *Pce*_S__f. Iso-Seq results are summarized in **Table S3**. In total, 248,218 high-quality isoforms have been identified. Both the high- and the low-quality isoforms have been used for genome annotation where each gene might be represented by multiple isoforms. A total of 107,508 transcripts (*Pce*_S__a: 53,497; *Pce*_S__f: 54,011) were predicted from the 60,123 gene models (*Pce*_S__a: 29,069; *Pce*_S__f: 31,054) obtained by structural annotation procedures (**Table S4**). Interproscan analysis detected 1,381,841 functional annotations (*Pce*_S__a: 649,310; *Pce*_S__f: 687,531) using 16 databases. Two-thirds (71,870) of the transcripts were assigned with GO terms and 9,114 were found to be involved in annotated pathways.

**Table 1:** Characterization of repetitive sequences of *P. fruticosa* ecotype Hármashatárhegy (*Pf*_eH_) compared to *P. avium* Tieton (*Pa*_T_)*, P. persica* Lovell, and the two subgenomes of *P. cerasus* ‘Schattenmorelle’ *Pce*_S__a and *Pce*_S__f.

Class	Order	Family	No. of elements					Length (bp)					Percentage of the genome (%)				
			*Pf*_eH_	*Pa*_T_	*Pp*	*Pce*_S__a	*Pce*_S__f	*Pf*_eH_	*Pa*_T_	*Pp*	*Pce*_S__a	*Pce*_S__f	*Pf*_eH_	*Pa*_T_	*Pp*	*Pce*_S__a	*Pce*_S__f
I (retro-transposons)	**LTR**	–	2142	1723	2607	2483	2800	472290	264621	446395	502909	589486	0.13	0.08	0.20	0.09	0.10
		Cassandra	1852	1179	753	962	1378	910040	323669	329299	338144	605740	0.25	0.09	0.15	0.06	0.11
		Caulimovirus	793	1586	697	1260	798	627333	861208	933515	1376203	692369	0.17	0.25	0.41	0.24	0.12
		**Copia**	**41192**	**32612**	**23578**	**25346**	**31727**	**27822528**	**12487829**	**14606294**	**15713285**	**21571049**	**7.59**	**3.64**	**6.47**	**2.76**	**3.79**
		**Gypsy**	**68445**	**45868**	**25860**	**41178**	**55259**	**76652400**	**20554372**	**19004947**	**41439100**	**57972951**	**20.91**	**5.99**	**8.42**	**7.29**	**10.20**
		**ERV1**	**-**	**94**	**-**	**317**	**306**	**-**	**17404**	**-**	**29672**	**32867**	**-**	**0.01**	**-**	**0.01**	**0.01**
		**ERVK**	**-**	**-**	**195**	**35**	**29**	**-**	**-**	**41513**	**20720**	**17722**	**-**	**-**	**0.02**	**0.004**	**0.003**
		**Pao**	**344**	**-**	**200**	**265**	**235**	**96802**	**-**	**156072**	**133246**	**117728**	**0.03**	**-**	**0.07**	**0.02**	**0.02**
	LINE	I-Jockey	413	464	107	–	–	140619	110916	38834	–	–	0.04	0.03	0.02	–	–
		L1	8844	6349	4286	6703	7722	4167515	2449897	1392308	2637481	3118916	1.14	0.71	0.62	0.46	0.55
		L2	434	–	110	–	–	64430	–	21600	–	–	0.02	–	0.01	–	–
		Penelope	176	–	218	–	–	25448	–	34866	–	–	0.01	–	0.02	–	–
		RTE-BovB	516	214	–	201	281	87801	91608	–	62501	79770	0.02	0.03	–	0.01	0.01
		**L1-Tx1**	**-**	**-**	**211**	**-**	**-**	**-**	**-**	**46145**	**-**	**-**	**-**	**-**	**0.02**	**-**	**-**
		**R1-LOA**	**-**	**-**	**-**	**88**	**72**	**-**	**-**	**-**	**27316**	**17623**	**-**	**-**	**-**	**0.005**	**0.003**
		**RTE-X**	**-**	**-**	**-**	**38**	**82**	**-**	**-**	**-**	**52387**	**55967**	**-**	**-**	**-**	**0.01**	**0.01**
		**R2-NeSL**	**-**	**-**	**184**	**-**	**-**	**-**	**-**	**36760**	**-**	**-**	**-**	**-**	**0.02**	**-**	**-**
		**Rex-Babar**	**-**	**-**	**89**	**-**	**-**	**-**	**-**	**30378**	**-**	**-**	**-**	**-**	**0.01**	**-**	**-**
		**CR1**	**-**	**-**	**712**	**-**	**-**	**-**	**-**	**609177**	**-**	**-**	**-**	**-**	**0.27**	**-**	**-**
		**TAD1**	**-**	**37**	**-**	**-**	**-**	**-**	**6991**	**-**	**-**	**-**	**-**	**0.002**	**-**	**-**	**-**
	SINE	–	457	620	1886	2039	1854	62956	49823	192330	213023	193058	0.02	0.01	0.09	0.04	0.03
		**ID**	**-**	**-**	**1450**		**-**	**-**	**-**	**121213**	**-**	**-**	**-**	**-**	**0.05**	**-**	**-**
		**tRNA-DEU-L2**	**-**	**-**	**-**	**587**	**578**	**-**	**-**	**-**	**45058**	**46403**	**-**	**-**	**-**	**0.01**	**0.01**
		**tRNA-Core-L2**	**-**	**-**	**1109**		**-**	**-**	**-**	**122977**	**-**	**-**	**-**	**-**	**0.05**	**-**	**-**
		B2	1517	123	–	670	619	122973	9505	–	49331	46555	0.03	0.003	–	0.01	0.01
		tRNA	4593	5378	2229	3110	3118	509966	517003	203010	283183	283895	0.14	0.15	0.09	0.05	0.05
II (DNA transposons)	TIR	TcMar-Fot1	276	123	–	–	–	210022	48369	–	–	–	0.06	0.01	–	–	–
Subclass I		**hAT-Charlie**	**-**	**-**	**1984**	**-**	**-**	**-**	**-**	**468323**	**-**	**-**	**-**	**-**	**0.21**	**-**	**-**
		**IS3EU**	**-**	**103**	**-**	**-**	**-**	**-**	**19992**	**-**	**-**	**-**	**-**	**0.01**	**-**	**-**	**-**
		**P**	**-**	**141**	**-**	**-**	**-**	**-**	**23742**	**-**	**-**	**-**	**-**	**0.01**	**-**	**-**	**-**
		**Sola-3**	**-**	**111**	**-**	**-**	**-**	**-**	**42070**	**-**	**-**	**-**	**-**	**0.01**	**-**	**-**	**-**
		**TcMAr**	**-**	**-**	**51**	**-**	**-**	**-**	**-**	**2513**	**-**	**-**	**-**	**-**	**0.00**	**-**	**-**
		**TcMAr-Tigger**	**-**	**-**	**152**	**-**	**-**	**-**	**-**	**121325**	**-**	**-**	**-**	**-**	**0.05**	**-**	**-**
		**Zisupton**	**-**	**-**	**141**	**-**	**-**	**-**	**-**	**18330**	**-**	**-**	**-**	**-**	**0.01**	**-**	**-**
		**TcMar-ISRm11**	**81**	**55**	**-**	**-**	**-**	**24496**	**21760**	**-**	**-**	**-**	**0.01**	**0.01**	**-**	**-**	**-**
		**TcMar-Mariner**	**-**	**-**	**-**	**240**	**190**	**-**	**-**	**-**	**57867**	**51568**	**-**	**-**	**-**	**0.01**	**0.01**
		hAT-Ac	11533	14886	6511	7832	10293	3430880	2101683	2127280	2654943	3223143	0.94	0.61	0.01	0.47	0.57
		hAT-Tag1	5353	5258	3219	3462	3909	1263452	971199	896657	799082	899518	0.34	0.28	0.40	0.14	0.16
		hAT-Tip100	9680	8622	6079	6784	7869	2348735	1677234	1336903	1302197	1611332	0.64	0.49	0.59	0.23	0.28
		PIF-Harbinger	14230	14435	9390	10305	10886	4268364	3498800	4123535	3331917	3337330	1.16	1.02	1.83	0.59	0.59
		**PIF-Spy**	**-**	**-**	**-**	**31**	**22**	**-**	**-**	**-**	**1588**	**1094**	**-**	**-**	**-**	**0.0003**	**0.0002**
Subclass II	Crypton	**Crypton-H/A**	**237**	**-**	**-**	**90**	**146**	**195974**	**-**	**-**	**19058**	**31113**	**0.05**	**-**	**-**	**0.003**	**0.005**
	Maverick	Maverick	576	357	–	172	333	155067	76204	–	70984	138588	0.04	0.02	–	0.01	0.02
	Helitron	Helitron	5378	4241	3584	2534	3367	2220498	1496918	1255959	1016700	1295589	0.61	0.44	0.56	0.18	0.23
		unknown/Helitron	228	78	92	106	92	155920	17774	44752	84000	71994	0.04	0.01	0.02	0.01	0.01
Other		–	13120	9946	9302	8616	9926	2310744	1774664	1865367	1592948	1972787	0.63	0.52	0.83	0.28	0.35
		**Academ /-2**	**42**	**-**	**-**	**479**	**590**	**20252**	**-**	**-**	**129228**	**147664**	**0.01**	**-**	**-**	**0.02**	**0.03**
		**CMC-EnSpm**	**16958**	**14886**	**10222**	**12443**	**14104**	**8879643**	**4747818**	**12856725**	**7174757**	**7768726**	**2.42**	**1.38**	**5.70**	**1.26**	**1.37**
		**Dada**	**-**	**-**	**-**	**110**	**113**	**-**	**-**	**-**	**51998**	**50989**	**-**	**-**	**-**	**0.01**	**0.01**
		Ginger	325	–	99	–	–	77794	–	12430	–	–	0.02	–	0.01	–	–
		MULE-MuDR	17464	16744	9606	14069	14558	4459943	3751469	3049949	3668069	3844162	1.22	1.09	1.35	0.65	0.68
rRNA			326	30	223	84	346	231622	10508	334205	66518	473346	0.06	0.003	0.15	0.01	0.08
snRNA			–	55	127	–	–	–	4318	21457	–	–	–	0.001	0.01	–	–
Satellite			870	397	342	229	244	220737	88777	137022	53968	55261	0.06	0.03	0.06	0.01	0.01
Simple repeat			106232	81995	77870	82364	84513	4353840	3831673	6797949	3270826	3450293	1.19	1.12	3.01	0.58	0.61
Low complexity			19611	15501	13876	15436	16617	984829	756687	653107	769005	839253	0.27	0.22	0.29	0.14	0.15
**Unknown**			**168094**	**171338**	**99691**	**208161**	**179132**	**42110587**	**41991523**	**21700006**	40951426	37691937	**11.49**	**12.25**	**9.61**	**7.20**	**6.63**
**SUM**			**522228**	**450112**	**319042**	**458829**	**464108**	**189663955**	**104698028**	**96191427**	**129990638**	**152397786**	**51.75**	**30.53**	**42.62**	**48.32**	**50.89**

Bold values indicate repetitive elements which represent the highest proportion or uniqueness in the investigated datasets.

### 3.3 Completeness and quality of the genome and transcriptome

**Figure 3:**
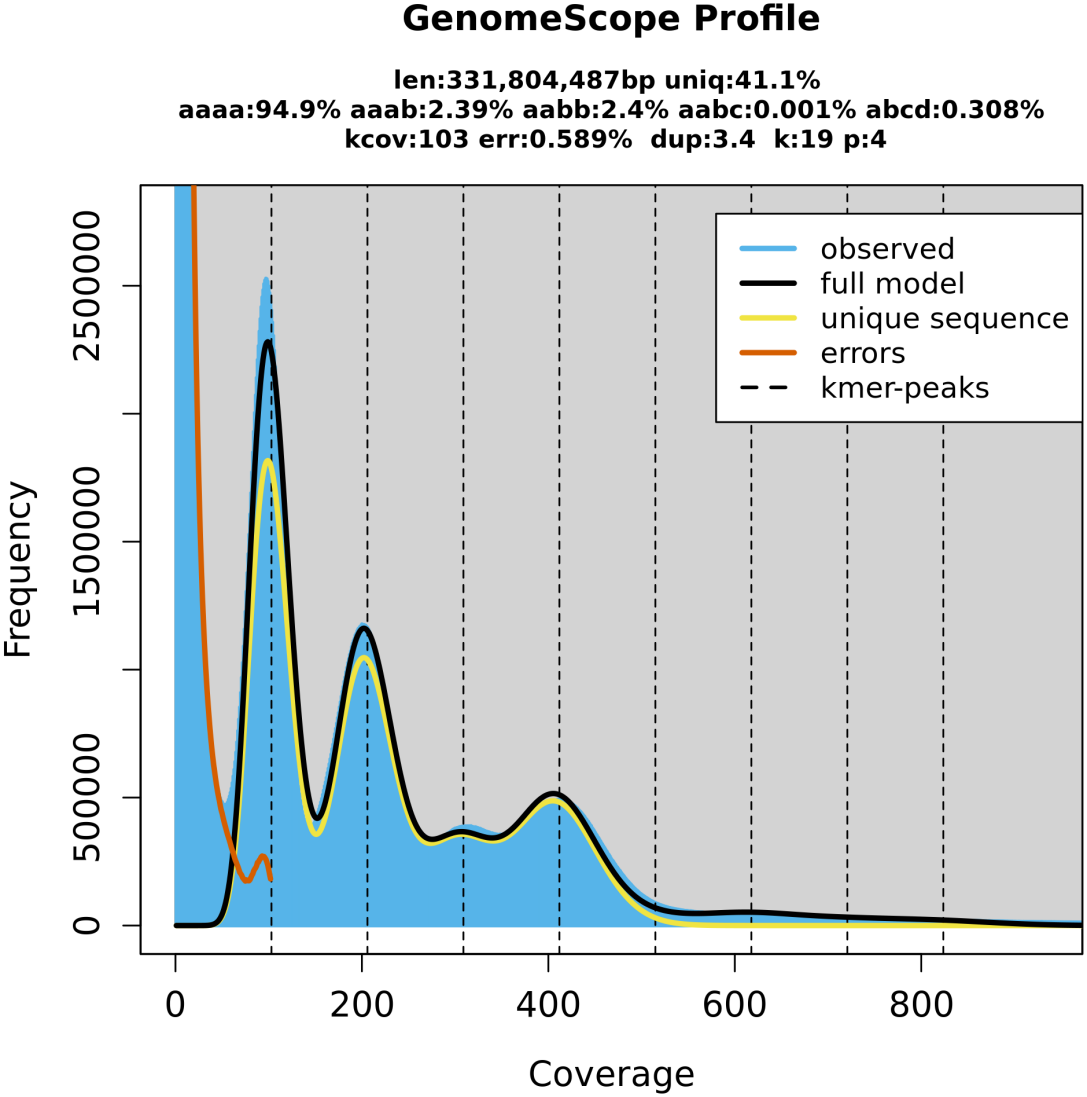
GenomeScope (Galaxy Version 2.0) estimation of the *P. cerasus* genome size by k-mer counts obtained from the software Meryl (Galaxy Version 1.3+galaxy2). Both programs are integrated on the GalaxyServerEurope. The k-mer peaks indicate that k-mers with a length of 19 bp occur in heterozygote (100× depth, 200× depth, 300× depth) and homozygote (400× depth) constitution within the genome. Coverage depth of individual k-mers is assigned as coverage.

BUSCO completeness of the *Pce*_S_ genome was 99.0% (S: 16.7%, D: 82.3%, F: 0.4%, M: 0.6%, n: 1,614) respectively and comparable with *P. persica* Lovell (99.3%) and *P. avium* Tieton (98.3%, **Figure S4**). Completeness of subgenome *Pce*_S__a was higher (C: 89.4%, S: 84.8%, D: 4.6%, F: 1.5%, M: 9.1%, n: 1,614) compared to subgenome *Pce*_S__f (C: 87.1%, S: 80.9%, D: 6.2%, F: 1.2%, M: 11.7%, n: 1,614). The calculated LAI index was 6.3 and low in comparison to other genomes (*Pp*_L_: 17.6, *Pa*_T_: 10.3, *Pf*_eH_: 13.1). The LAI index for subgenome *Pce*_S__a was 7.1. The LAI index for subgenome *Pce*_S__f was 5.6 (**Figure S5**). The nucleotide heterozygosity rates were 94.9% for aaaa, 2.39 for aaab, 2.4 for aabb, 0.001 for aabc, and 0.308 for abcd (**Figure 3**). The comparison of genetic position and physical position of up to 1,856 markers of the five genetic sour cherry maps (**Table S5A**) showed a good co-linearity to the genome sequence (**Figure S6**). BUSCO evaluation on completeness of the annotated proteins resulted in 99.2% [C: 99.2% (S: 8.4%, D: 90.8%), F: 0.4%, M: 0.4%, n: 1,614]. The chloroplast sequence obtained contained 427 genes, 21 rRNAs, and 136 tRNAs, whereas the mitochondrial sequence contained 188 genes, 3 rRNAs, and 152 tRNAs (**Figure S3**). An *ab initio* and homology-based gene prediction with 14 reference species was performed (IAA). Based on the homology prediction, 34% of the proteins showed the highest IAA towards *Prunus fruticosa* and 17.9% towards *P. avium*. Only 5.2% of the proteins showed no IAA to any of the used reference datasets used, which was due to *ab initio* prediction. The data are summarized in **Figure S7**.

**Table 2A** shows the general BUSCO statistics of transcriptomic data. A comparison of transcripts of *Pce*_S_ and the annotation datasets of *Pf*_eH_ and *Pa*_T_ enabled a quantitative comparison of shared transcripts within the datasets (**Table 2B**). A total number of 26,532 shared transcripts were found between the two subgenomes *Pce*_S__a and *Pce*_S__f and the genomes of *Pf*_eH_ and *Pa*_T_. Thirty-eight percent of the *P. cerasus* proteins had a greater IAA to *Pf*_eH_, whereas 54% showed a greater IAA to *Pa*_T_. Eight percent showed an identical IAA to both ancestral species. A larger number of transcripts of both sour cherry subgenomes (**Table 2B**) were assigned to the annotation dataset of *Pf*_eH_. A total of 13,425 transcripts from the *Pce*_S__a subgenome and 13,107 from the *Pce*_S__f subgenome were found in the genome sequences of *Pf*_eH_ and *Pa*_T_. Seventy-five percent of the pool from the *Pce*_S__a subgenome showed a higher IAA to *Pa*_T_ and 17% to *Pf*_eH_, while 59% from the pool originating from the *Pce*_S__f subgenome showed a higher IAA to *Pf*_eH_ and 32% to *Pa*_T_.

**Table 2A:** BUSCO statistics of the transcriptomic data generated in this study (n:1614).

Assembly	*Pce_S_*		*Pce_S__avium*		*Pce_S__fruticosa*	
Level			Chr.	Chr. + UC	Chr.	Chr. + UC
BUSCO (%)	C:	99.2	89.0	94.7	84.7	91.6
	S:	8.4	43.0	45.8	41.6	44.2
	D:	90.8	46.0	48.9	43.1	47.4
	F:	0.4	1.3	1.3	2.0	1.9
	M:	0.4	9.7	4.0	13.3	6.5

**Table 2B:** Comparison between the number of transcripts and %-IAA obtained from P. fruticosa ecotype Hármashatárhegy PfeH and P. avium cv ‘Tieton’ PaT representing the two ancestral species of P. cerasus.

Chromosome	No. of transcripts	No. obtained from donor species			%higher iAA to		
		*Pf*_eH_	*Pa*_T_	*Pf*_eH_ & *Pa*_T_	*Pf*_eH_	*Pf*_eH_ & *Pa*_T_	*Pa*_T_
*Pce*_S__a _Chro1	10675	6162	3953	2934	0.12	0.07	0.81
*Pce*_S__a _Chro2	6511	3757	2301	1713	0.18	0.09	0.73
*Pce*_S__a _Chro3	5597	3248	2019	1491	0.17	0.07	0.76
*Pce*_S__a _Chro4	4969	2830	1743	1281	0.19	0.07	0.74
*Pce*_S__a _Chro5	4446	2575	1627	1242	0.15	0.05	0.80
*Pce*_S__a _Chro6	6963	4212	2555	1986	0.19	0.07	0.74
*Pce*_S__a _Chro7	5280	3025	1969	1418	0.21	0.07	0.72
*Pce*_S__a _Chro8	5257	3100	1860	1360	0.21	0.11	0.68
*Pce*_S__f _Chro1	10133	6345	3495	2835	0.56	0.09	0.35
*Pce*_S__f _Chro2	6254	3865	2056	1602	0.53	0.13	0.34
*Pce*_S__f _Chro3	5869	3686	1953	1564	0.61	0.09	0.30
*Pce*_S__f _Chro4	5362	3296	1820	1411	0.61	0.09	0.29
*Pce*_S__f _Chro5	4032	2511	1425	1172	0.64	0.07	0.29
*Pce*_S__f _Chro6	6968	4512	2441	1981	0.60	0.09	0.31
*Pce*_S__f _Chro7	5033	3201	1687	1312	0.60	0.09	0.31
*Pce*_S__f _Chro8	4925	3158	1566	1230	0.61	0.12	0.28
Sum							
*Pce*_S__a	49698	28909	18027	13425	0.17	0.07	**0.75**
*Pce*_S__f	48576	30574	16443	13107	**0.59**	0.10	0.32
*Pce*_S_	98274	59483	34470	26532	0.38	0.08	0.54

Bold values indicate repetitive elements which represent the highest proportion or uniqueness in the investigated datasets.

### 3.4 Identification of syntenic regions and inversions

The sequences of the two subgenomes *Pce*_S__a and *Pce*_S__f and the genotypes *Pa*_T_ and *Pf*_eH_ of the two ancestral species *P. avium* and *P. fruticosa* were screened for duplicated regions using DAGchainer as previously published for peach (International Peach Genome Initiative et al., 2013). The seven major triplicated regions were found nearly one to one in *P. avium* but not in *P. fruticosa*, which lacked regions 4 and 7 corresponding to International Peach Genome Initiative et al., 2013. *P. avium* and *P. fruticosa* seem to derive from the same paleohexaploid event like peach, but with a loss of the fourth and seventh paleoset of paralogs in *P. fruticosa.* The graphical analysis is summarized in **Figure S8**.

Thirteen inversions were detected through positional co-linearity comparison between the two subgenomes using the molecular markers from the 9 + 6k SNP array (**Figure S9**). Five inversions were found between subgenome *Pce*_S__a and the genome sequence of *Pa*_T_. Eleven inversions were found between subgenome *Pce*_S__f and *Pf*_eH_ (**Table S6**). By comparing the position of amino acid sequences of orthologous proteins (synteny), we found 21 inversions when comparing *Pce*_S__f with *Pf*_eH_. Only 7 were found between *Pce*_S__a and *Pa*_T_ and 16 were found between both subgenomes *Pce*_S__a and *Pce*_S__f (**Figure S10**).

### 3.5 Detection of *de novo* homoeologous exchanges

**Figure 4:**
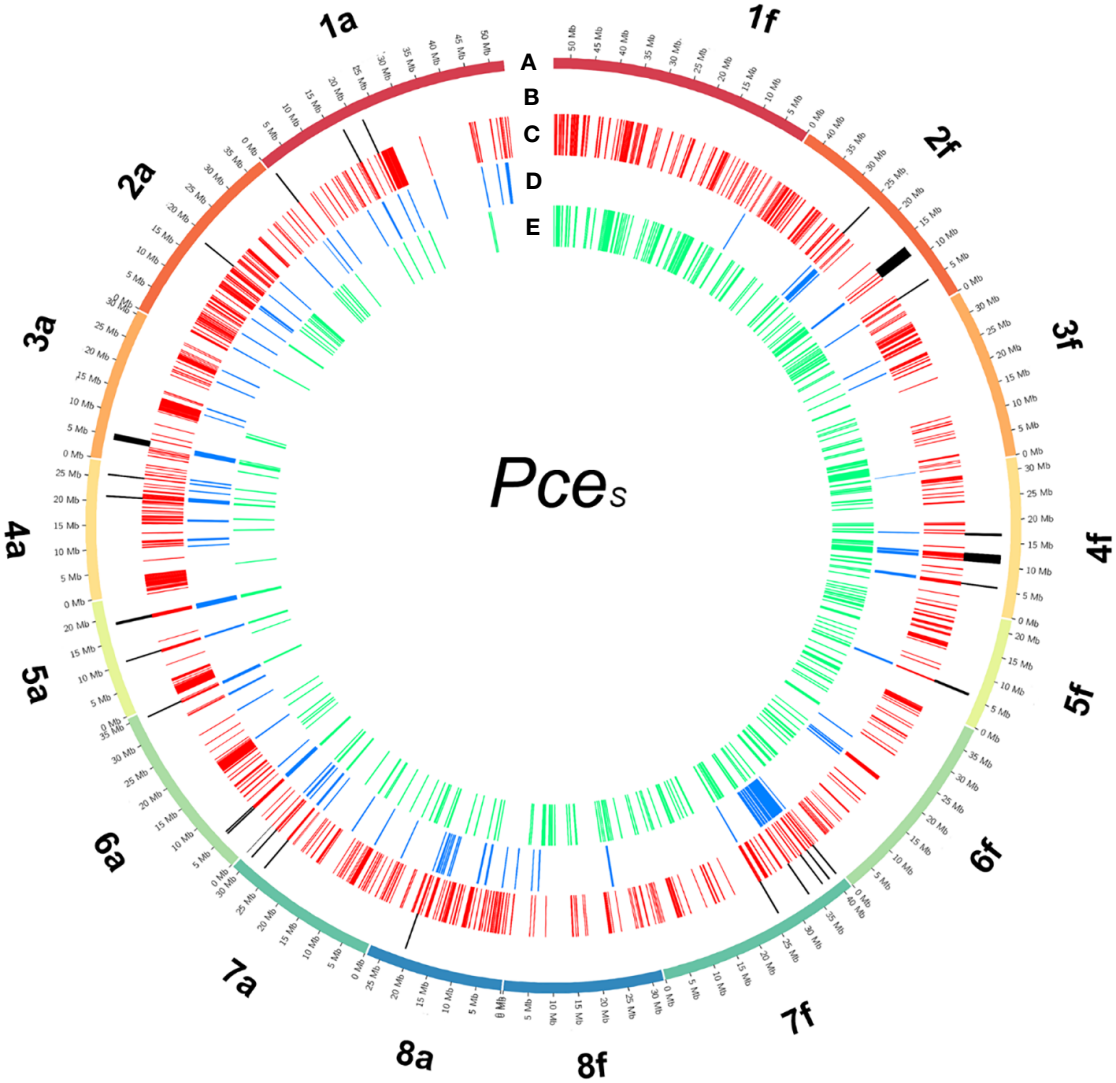
Detected regions of homoeologous exchanges in the genome of *P. cerasus* ‘Schattenmorelle’. Circos plot of 16 pseudomolecules of the subgenomes of *Pce*_S__a and *Pce*_S__f. **(A)** Chromosome length (Mb); **(B)** 16 in *Pce_S_* _a and 12 in *Pce*_S__f detected regions that match all three following analysis methods: **(C)** 1,024 regions (100k window) were intraspecific %-covered bases from mapped reads (*Pce*_S__a to *Pa*_T_, *Pce*_S__f to *Pf*_eH_) was less than interspecific %-covered bases from mapped reads (*Pce*_S__a to *Pf*_eH_, *Pce*_S__f to *Pa*_T_); **(D)** 148 regions were intraspecific difference of %-covered bases from obtained RNAseq reads (*Pa* and *Pce*_S__a, *Pf* and *Pce*_S__f) greater than interspecific difference of %-covered bases from obtained RNAseq reads (*Pf* and *Pce*_S__a, *Pa* and *Pce*_S__f); **(E)** 367 regions were the proportion of transcripts with intraspecific amino acid identity (*Pa*_T_ and *Pce*_S__a, *Pf*_eH_ and *Pce*_S__f) less than the proportion of transcripts with interspecific amino acid identity (*Pf*_eH_ and *Pce*_S__a, *Pa*_T_ and *Pce*_S__f).

For the detection of *de novo* homoeologous exchanges, we used three approaches by comparing inter- and intraspecific %-covered bases (genomic and transcriptomic) and %-IAA between proteins of *Pce*_S_ to *Pa*_T_ and *Pf*_eH_ (**Figure 4**; **Figure S11**). *Pce*_S_ short reads were mapped against *Pa*_T_ and *Pf*_eH_ and only species specific reads (*Pa*_T_ and *Pf*_eH_) were filtered into read subsets. The obtained read subsets were re-mapped against *Pce*_S__a and *Pce*_S__f and base coverage was calculated. A total of 1,024 regions (100k window) that were intraspecific %-covered bases from mapped reads (*Pce*_S__a to *Pa*_T_, *Pce*_S__f to *Pf*_eH_) and were less than interspecific %-covered bases from mapped reads (*Pce*_S__a to *Pf*_eH_, *Pce*_S__f to *Pa*_T_) were discovered. In a second approach, translocations between the two subgenomes were localized by short-read mapping analyses. Short-reads (RNAseq) from *P. cerasus, P. avium*, and *P. fruticosa* obtained from Bird et al. (2022) were mapped on *Pce*_S_. A total of 148 regions whose intraspecific difference of %-covered bases from obtained RNAseq reads (*Pa* and *Pce*_S__a, *Pf* and *Pce*_S__f) was greater than the interspecific difference of %-covered bases from obtained RNAseq reads (*Pf* and *Pce*_S__a, *Pa* and *Pce*_S__f) indicated homoeologous exchanges between the two subgenomes. Finally, 367 regions in which the proportion of transcripts with intraspecific amino acid identity (*Pa*_T_ and *Pce*_S__a, *Pf*_eH_ and *Pce*_S__f) was less than the proportion of transcripts with interspecific amino acid identity (*Pf*_eH_ and *Pce*_S__a, *Pa*_T_ and *Pce*_S__f) were identified (**Figure 4**). Several regions were confirmed by calculating the 70% quantile of the IAA value within a window of 1-Mbp windows (Note S1). This confirms that there are transcripts in the *Pce_S_* genome whose IAA to the homoeologous representative genome (*Pf*_eH_) is greater than that to the homologous (*Pa*_T_) representative. A total of 21 in *Pce*_S__a and 29 in *Pce*_S__f regions spanning 250k windows were finally identified that match all three criteria indicating *de novo* homoeologous exchanges within the subgenomes (**Figure 4**; **Figure S11**). No evidence for an introgression of other *Prunus* species was found (Note S1, Note S2). Using 14 reference species, 60,123 gene models were annotated. Almost the same number was assigned to the two *P. cerasus* subgenomes (**Table 2B**). No evidence was found for large introgressions from any of the reference species (Note S1, S3). By comparing the amino acid identity of the proteins of *Pa*_T_ and *Pf*_eH_ with the respective sour cherry subgenome, the identified translocations via read mapping could be confirmed. The majority of the transcripts (51%) could be assigned to the genotypes *Pa*_T_ and *Pf*_eH_ of the two ancestral species *P. avium* and *P. fruticosa* (**Figure S7**); 5.2% of the transcripts could not be assigned to any of the reference species. Only <1% of the transcripts could not be assigned to one of the ancestral species. They showed equivalent matches to both species and are probably a product of *ab initio* prediction. A total of 49,698 proteins in subgenome *Pce*_S__a and 48,576 proteins in *Pce*_S__f shared only 13,435 and 13,107 proteins with *Pa*_T_ and *Pf*_eH_, respectively. A total of 75% of the proteins of subgenome *Pce*_S__a matched better to *Pa*_T_ compared to *Pf*_eH_, whereas only 59% of *Pce*_S__f mapped better to *Pf*_eH_ than to *Pa*_T_ (**Table 2B**).

### 3.6 LTR dating and divergence of time estimation

**Figure 5:**
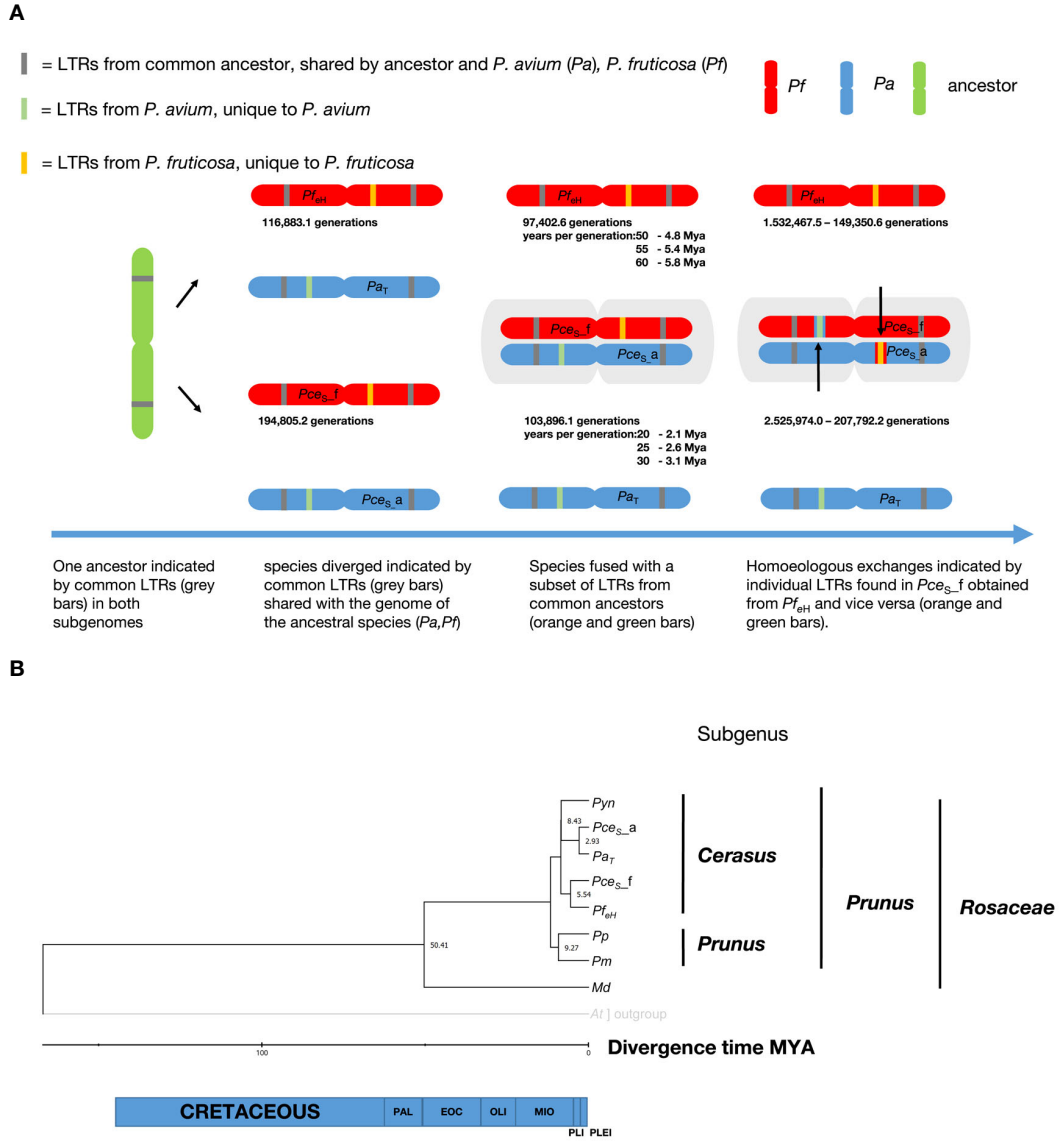
Investigation on the evolution of the genome of *P. cerasus* ‘Schattenmorelle’. **(A)** Determination of insertion time from shared long terminal repeats (LTRs) in *P. cerasus* subgenome *avium* (*Pce*_S__a) and *P. cerasus* subgenome *fruticosa* (*Pce*_S__f) compared to *P. avium* Tieton (*Pa*_T_) and *P. fruticosa* ecotype Hármashatárhegy (*Pf*_eH_). **(B)** Estimation of divergence of time (Mya) of *P. cerasus* subgenomes *Pce*_S__a and *Pce*_S__f compared to the donor species *P. avium* (Pa) and *P. fruticosa* (Pf)*. Prunus yedonensis* (Pyn); *Prunus avium* (Pa); *Prunus persica* (Pp); *Prunus mume* (Pm); *Malus domestica* (Md); Paleocene (PAL); Eocene (EOC); Oligocene (OLI); Miocene (MIO); Pliocene (PlI); Pleistocene (PLEI).

The left and the right LTR identity of a subset of 2,385 (*Pce*_S__a), 3,028 (*Pce*_S__f), 3,130 (*Pa*_T_), and 3,992 (*Pf*_eH_) LTRs were analyzed. The homologous genomes shared 200 (*Pce*_S__a versus *Pa*_T_) and 100 (*Pce*_S__f versus *Pf*_eH_) LTRs whereas 12 LTRs were shared by *Pce*_S__a versus *Pf*_eH_ and *Pce*_S__f versus *Pa*_T_. Only five common LTRs were found between *Pce*_S__a and *Pce*_S__f and 13 between *Pa*_T_ and *Pf*_eH_. A summary of the LTRs’ insertion time is shown in **Figure 5A**. The youngest shared LTRs between *Pce*_S__a and *Pa*_T_ and between *Pce*_S__f and *Pf*_eH_ were calculated with 103,896.1 and 97,402.6 generations, respectively. When comparing the homoeologous chromosomes, the youngest shared LTRs between *Pa*_T_ and *Pf*_eH_ and between *Pce*_S__a and *Pce*_S__f were calculated with 116,883.1 and 194,805.2 generations, respectively. LTRs of *Pa*_T_ were also found in subgenome *Pce*_S__f and calculated with 207,792.2 generations. LTRs of *Pf*_eH_ were detected in subgenome of *Pce*_S__a and calculated with 149,350.6 generations. This indicates an exchange of LTRs between the two subgenomes. A total of 834 single-copy orthogroups among nine genomes were found and used for single protein alignments. Single alignments were concatenated and a final alignment with nine amino acid sequences representing each species with 419,586 amino acid positions was used for phylogenetic tree construction. Using the RelTime method, the estimated divergence time between the genera *Malus* and *Prunus* was 50.4 Mya. The species groups *P. persica* and *P. mume* diverged from the *P. yedonensis/P. avium/P. fruticosa* group 11.6 Mya. Based on this model, the divergence of the two subgenomes of *P. cerasus* compared to the genome sequences of *Pa*_T_ and *Pf*_eH_ was estimated with 2.93 Mya and 5.5 Mya respectively (**Figure 5B**).

## 4 Discussion

The genome of the economically most important sour cherry ‘Schattenmorelle’ in Europe was sequenced using a combination of Oxford Nanopore R9.4.1 PromethION long-read technology and Illumina NovaSeq™ short-read technology. After assignment of the long reads to the two subgenomes and Hi-C analysis, the final assembly was 629 Mbp and showed an overall acceptable contiguity of the subgenome *P. avium* to a recently published genome of *P. cerasus* Montmorency - *Pce*_M_ (Goeckeritz et al., 2023). Larger differences were found between the haplotypes of *P. fruticosa*, which was expected (Note S4). This sequence was used to study structural changes present in the allotetraploid sour cherry genome after its emergence. Therefore, the sour cherry genome sequence was compared to the published genome sequences of *Prunus avium* Tieton (*Pa*_T_, Wang et al., 2020) and *Prunus fruticosa* ecotype Hármashatárhegy (*Pf*_eH_, Wöhner et al., 2021) representing genotypes of the two ancestral species. The size of the subgenome *Pce*_S_ originating from *P. avium* was 269 Mbp. A similar genome size (271 Mbp) is described for the *Prunus avium* Big Star (Pinosio et al., 2020) and Sato Nishiki (Shirasawa et al., 2017). Larger differences were found in Regina with 279 Mbp and Tieton with 344 Mbp (Le Dantec et al., 2018; Wang et al., 2020). Differences were also found between the size of subgenome *Pce*_S__f (299 Mbp) and the genome of the ground cherry genotype *P. fruticosa* ecotype Hármashatárhegy (366 Mbp, Wöhner et al., 2021). These differences indicate a reduction of the subgenome *Pce*_S__a by 0.49%–21%, whereas for subgenome *Pce*_S__f, a reduction of 18.29% was found. This was further confirmed by a reduced genome size of the recently published subgenome sequences of *Pce*_M_ (**Table 3**) published by Goeckeritz et al. (2023). The reduction in genome size for allotetraploid species in comparison to their ancestral genomes was reported for *Nicotiana tabacum* (1.9%–14.3%) and *Gossypium* species (Leitch et al., 2008; Hawkins et al., 2009; Renny-Byfield et al., 2011), with an overall downsizing rate for angiosperms calculated as 0%–30% (Zenil-Ferguson et al., 2016). Genome downsizing in response to a genome hybridization event can be explained with evolutionary advantages, which give these species with smaller genomes a selection advantage in the long term (Knight et al., 2005; Zenil-Ferguson et al., 2016). Although downsizing of the *P. cerasus* subgenomes is most probable, enlargement and expansion of the genomes of ancestral species during evolution would be another possibility. However, an increase in genome size during the evolution of a species has only rarely been documented (Jakob et al., 2004; Leitch et al., 2008; Kim et al., 2014). BUSCO analysis provides additional evidence for the reduction of genome size. Although the number of genes does not correlate with genome size in eukaryotes (Pierce, 2012), differences between the ancestral genomes and sour cherry could be observed when looking at BUSCO completeness (**Table 3**). Considering both subgenomes and the genomes of *Pa*_T_ and *Pf*_eH_, a completeness of >96.4% was obtained. However, the completeness of the single subgenomes was only 89.4% for *Pce*_S__a and 87.1% for *Pce*_S__f. This could also be observed for *Pce*_M_ (**Table 3**). Comparisons (Note S5) within the subgenomes of *Pce*_S_ and *Pce*_M_ showed a loss between 2.4% and 6.4% of BUSCOs (NoteS5, A), between 0.6% and 0.7% when comparing the subgenomes of *Pce*_S_ and *Pce*_M_ (Note S5, B), and between 3.3% and 6.9% between the subgenomes of *Pce*_S_ and *Pce*_M_ (Note S5, C) and the representing ancestral genomes (*Pa*_T_ and *Pf*_eH_). Structural differences between the *P. cerasus* subgenomes and the genomes of *Pa*_T_ and *Pf*_eH_ were also found by comparing the number of repetitive elements. While the content of repetitive elements differs by only 0.86% between *Pf*_eH_ and *Pce*_S__f, it is 17.8% between *Pa*_T_ and *Pce*_S__a. Whether this is a consequence of hybridization remains speculative and would deserve further studies. An increase of class I elements Gypsy from 6% in the *Pa*_T_ genome to 7.3% in subgenome *Pce*_S__a indicates an expansion of this class following the formation of the sour cherry genome or a possible reduction of non-repetitive sequences in the corresponding subgenome resulting in a smaller genome size.

**Table 3:** BUSCO and assembly statistics of the genomic data generated in this study and comparative datasets (n:1614).

Prunus spec.		*cerasus*			*avium*		*fruticosa*		*avium*		*fruticosa*		*cerasus*		*avium*	*fruticosa*	
Assembly*		*Pce_S_*	*Pce_S_*		*Pce_S_* _a		*Pce_S_* _f		*Pa_T_*		*Pf*_eH_		*Pce*_M_		*Pce_M_* _B	*Pce_M_* _A	*Pce_M_* _A_
Level		Ct	Chr	Chr + UC	Chr	Chr + UC	Ch.	Chr + UC	Chr	Chr + UC	Chr	Chr + UC	Chr	Chr + UC	Chr	Chr	Chr
BUSCO (%)	C:	99.4	97.4	99.2	89.5	95.7	87.1	94.1	97.8	97.8	96.9	96.9	99.0	99.5	91.1	93.1	95.5
	S:	4.6	26.3	16.7	84.9	90.4	80.9	85.9	91.4	91.4	85.0	84.9	5.3	1.5	86.9	90.5	93.0
	D:	94.8	71.1	82.5	4.6	5.3	6.2	8.2	6.4	6.4	11.9	12.0	93.7	98.0	4.2	2.6	2.5
	F:	0.2	0.6	0.4	1.5	1.4	1.2	1.1	0.7	0.7	1.2	1.2	0.3	0.2	1.3	0.7	0.6
	M:	0.4	2.0	0.4	9.0	2.9	11.7	4.8	1.5	1.5	1.9	1.9	0.7	0.3	7.6	6.2	3.9
Assembly statistics	Number of scaffolds	3750	16	236	8	94	8	142	8	61	8	57	24	3592	8	8	8
	Number of contigs	3750	1800	2020	935	1021	865	999	557	610	1198	1247	760	4765	438	196	126
	Total length (Mbp)	1.071,9	568,5	628,2	269,0	291,7	299,5	336,4	342,9	344,3	366,5	375,3	771,8	1.066,0	250,5	262,8	258,6
	Percent gaps	0.00	0.03	0.03	0.03	0.03	0.03	0.03	0.02	0.02	0.03	0.03	0.05	0.06	0.09	0.04	0.02
	Scaffold N50 (Mbp)	0.38	35	34	31	31	39	39	42	42	43	43	31	28	28	31	31
	Contigs N50 (Mbp)	0.38	0.5	0.5	0.44	0.43	0.57	0.57	3	3	0.54	0.53	4	2	1	6	10

*Pce_S_ - ‘Schattenmorelle’, *Pce_S_* _a – subgenome *P. avium*, *Pce_S_* _f – subgenome *P. fruticosa*, *Pa_T_* – Tieton, *Pf_eH_* –ecotype Hármashatárhegy, *Pce*_M_ - Montmorency, *Pce_M_* _B – subgenome *P. avium*, *Pce_M_* _A – subgenome *P. fruticosa* haplotype 1, *Pce_M_* _A_– subgenome *P. fruticosa* haplotype 2.

Ct, contig scale; Chr, chromosome scale; UC, unassigned contigs; C, complete busco; S, single busco; D, duplicated busco; F, fragmented busco; M, missing busco.

A comparison of syntenic regions showed a high degree of collinearity between *Pce*_S_, *Pa*_T_, and *Pf*_eH_ genomes (**Figure 2**; **Figures S8, S9**), with single inversions between the respective chromosome pairs. Using the genome of *P. persica*, seven triplicated regions were detected in *Pce*_S__a, confirming that these genomes descend from a palaeohexploid ancestor. However, the triplicated regions 4 and 7 in *Pce*_S__f were only detected in highly fragmented form or have been lost. Hao et al. (2022), who described a rapid loss of homoeologs immediately after polyploidy events, described a similar finding. Based on Ranallo-Benavidez et al. (2020), the results from the k-mer analysis confirm that the genome of sour cherry can be considered as highly heterozygous and segmental allotetraploid. Furthermore, genomes of segmental allopolyploids may possess a mix of auto- and allopolyploid segments through duplication–deletion events as a result of homoeologous exchanges leading to either reciprocal translocations or homoeologous non-reciprocal translocations (Mason and Wendel, 2020). Whereas autotetraploids have an aaab > aabb rate, allotetraploids are considered to have aaab < aabb. The near identical rate between aaab and aabb in *Pce*_S_ provides strong evidence that the sour cherry is a segmental mix of auto- and allotetraploidy.

Due to this assumption of segmental allotetraploidy, homoeologous recombination between homoeologous chromosomes is very likely. This is confirmed by the coverage and amino acid identity analyses. Homoeologous exchange events between the chromosomes of subgenome *Pce*_S__a and *Pce*_S__f were detected (**Figure 4**; **Figure S11**). These exchanges are not balanced but probably a product of a duplication/deletion event as described by Mason and Wendel (2020), generating the proposed mosaic of genomic regions representing one or the other subgenome. In this study, we found 14 homoeologous exchanges in *Pce*_S__a and 3 in *Pce*_S__f within one assembled contig obtained from the primary assembly 20-WGS-PCE.1.0 and 33 spanning multiple contigs (Note S6). Whether these regions were a result of incorrect assembly was not investigated and could be part of future studies.

Evidence that subgenome *Pce*_S__a seems to be closer to *Pa*_T_ than *Pce*_S__f to *Pf*_eH_ was found. This was confirmed by an evolutionary approach that calculated the separation of the subgenome *Pce*_S__f from *P. fruticosa* 5.5 Mya, while subgenome *Pce*_S__a separated from *P. avium* 2.93 Mya (**Figure 5B**).

To validate these results, the insertion events of long terminal repeats between *P. avium, P. fruticosa*, and the subgenomes were calculated. Assuming a *Prunus*-specific rate of 7.7 × 10^−9^ mutations per generation (Xie et al., 2016), LTRs of the same type with the same insertion time were identified in the same positional order in the different (sub)genomes. The most recent co-occurring LTRs between the genomes of *Pa*_T_ and *Pf*_eH_ could be dated at 116,883.1 generations. Exact data on the duration of the generation time of *Prunus* species in natural habitats do not exist. Although the juvenile phase of many *Prunus* species is usually completed after 5 years (Besford et al., 1996), it can be assumed that the times for a generation are considerably higher. Many fruit species are hardly or not at all able to rejuvenate by seeds under natural conditions (Coart et al., 2003), or they rejuvenate mainly by root suckers (Li et al., 2022). Other studies on *Prunus* therefore assume a duration of 10 years per generation (Wang et al., 2021), although even this seems rather too little.

Assuming that a generation change is to be expected after 10 to 60 years (Besford et al., 1996), this would correspond to a time period of ~1 to 6 Mya. The youngest co-occurring LTR could be estimated at 1.9 Mya. This suggests that *P. fruticosa* and *P. avium* probably shared a gene pool between ~1 Mya and 2 Mya. It should be noted that this estimate can vary greatly depending on the number of years per generation used in the calculation (**Figure 5A**). Based on the results of the protein dating, a generation time of 30 years is more likely for *P. avium*. For *P. fruticosa*, which occurs less frequently in natural habitats and reproduces mainly via root suckers, the generation time seems to be somewhat longer at 55 to 60 years. Some LTRs present in *Pa*_T_, but absent in *Pf*_eH_, were found in subgenome *Pce*_S__f only and *vice versa*. Other class I elements (LTR - ERV1, Pao) and Academ/-2 were specifically detected in one of the two genotypes *Pa*_T_ and *Pf*_eH_ representing the two ancestral species of sour cherry and in *Pce*_S__a and *Pce*_S__f, which indicates a transfer of these elements between the two subgenomes following the formation of the allotetraploid *P. cerasus* genome. This is a further indication for a segmental exchange between the two sour cherry subgenomes. Mason and Wendel (2020) speculated that unidirectional homoeologous exchange was observed in recent or synthetic allopolyploids. However, our results confirm this hypothesis by the evidence that sour cherry is a recent allopolyploid with autopolyploid segments derived from unidirectional homoeologous exchanges.

## 5 Conclusion

Sequencing of the genome of the European sour cherry cv. ‘Schattenmorelle’ has provided strong evidence that it is indeed a segmental allotetraploid consisting of two subgenomes, one derived from the sweet cherry *P. avium* and one from the ground cherry *P. fruticosa*. DNA sequences have been repeatedly exchanged between the two subgenomes. Our findings differ slightly from the recently sequenced genome of Montmorency—the sour cherry cultivar predominant in the US (Goeckeritz et al., 2023). Although Montmorency was shown to possess two subgenomes inherited from *P. avium* and *P. fruticosa*, it inherited two copies of the same subgenome from the former and two distinct subgenomes from the latter, making it trigenomic (Goeckeritz et al., 2023). We could not show that ‘Schattenmorelle’ is trigenomic. This discrepancy between both studies could be attributed to the sequencing technologies used. Whereas we used Illumina and Oxford Nanopore long read, Goeckeritz et al. (2023) used PacBio Sequel II. At the same time, a reduction in genome size has taken place. Other *Prunus* species have not contributed to the evolution of this species. No evidence was found for introgressions in the sour cherry genome derived from *Prunus* species other than *P. avium* and *P.* ‘Schattenmorelle’ approximately 1 Mya at the earliest.

## Data availability statement

The datasets presented in this study can be found in online repositories. The names of the repository/repositories and accession number(s) can be found in the article/**Supplementary Material**.

## Author contributions

TW: Conceptualization, Data curation, Formal Analysis, Investigation, Methodology, Project administration, Software, Supervision, Writing – original draft, Writing – review & editing. OE: Conceptualization, Validation, Writing – original draft, Writing – review & editing. AW: Conceptualization, Data curation, Investigation, Methodology, Project administration, Software, Validation, Writing – original draft. KN: Data curation, Investigation, Methodology, Software, Writing – original draft. RW: Data curation, Investigation, Methodology, Software, Writing – original draft. E-JB: Data curation, Investigation, Methodology, Software, Writing – original draft. HS: Data curation, Investigation, Methodology, Software, Writing – original draft. JK: Conceptualization, Data curation, Investigation, Methodology, Software, Writing – original draft. TB: Data curation, Investigation, Writing – review & editing. KH: Conceptualization, Investigation, Methodology, Software, Writing – original draft. LG: Data curation, Investigation, Methodology, Software, Writing – original draft. HT: Investigation, Methodology, Software, Writing – original draft. OA: Conceptualization, Methodology, Software, Writing – review & editing. LB: Conceptualization, Validation, Writing – review & editing. MS: Conceptualization, Validation, Writing – review & editing. JL: Formal Analysis, Software, Writing – original draft. AP: Conceptualization, Validation, Writing – original draft, Writing – review & editing. HF: Conceptualization, Supervision, Validation, Writing – original draft, Writing – review & editing.
