# Supplementary material for: The structure of the tetraploid sour cherry ‘Schattenmorelle’ (Prunus cerasus L.) genome reveals insights into its segmental allopolyploid nature
Source: Front Plant Sci. 2023 Dec 1;14:1284478. doi: 10.3389/fpls.2023.1284478 (PMC10722297; doi:10.3389/fpls.2023.1284478)
Supplement: Supplementary file 1 [file DataSheet_1.pdf]

The structure of the tetraploid sour cherry 'Schattenmorelle' (*Prunus cerasus* L.) genome reveals insights into its segmental allopolyploid nature

Thomas W. Wöhner[1], Ofere F. Emeriewen[1], Alexander H.J. Wittenberg[2], Koen Nijbroek[2], Rui Peng Wang[2], Evert-Jan Blom[2], Jens Keilwagen[4], Thomas Berner[4], Katharina J. Hoff[3], Lars Gabriel[3], Hannah Thierfeldt[3], Omar Almolla[5], Lorenzo Barchi[5], Mirko Schuster[1], Janne Lempe[1], Andreas Peil[1], Henryk Flachowsky[1]

[1]Julius Kühn Institute (JKI) – Federal Research Centre for Cultivated Plants, Institute for Breeding Research on Fruit Crops, Pillnitzer Platz 3a, D-01326, Dresden, Germany

[2]Keygene N.V., P.O. Box 216, 6700 AE Wageningen, Netherlands

[3]Institute of Mathematics and Computer Science, University of Greifswald, Walther-Rathenau-Str. 47, 17489 Greifswald, Germany

[4]Julius Kühn Institute (JKI) – Federal Research Centre for Cultivated Plants, Institute for Biosafety in Plant Biotechnology, Erwin-Baur-Str. 27, D-06484 Quedlinburg, Germany

[5]DISAFA – Plant genetics, University of Turin, Grugliasco (TO), 10095 Italy

[*]corresponding author: thomas.woehner@julius-kuehn.de

Keywords:

genome assembly, *P. cerasus*, sour cherry, tetraploid

This file contains information about supplemental material and methods

1. Supplemental material and methods

1.1 Plant Material and RNA extraction, sequencing and iso-seq analysis

Prunus cerasus L. 'Schattenmorelle' (accession KIZC99-2) young leaf material (tetraploid, small tree, size ca. 1,5 m, BioProject accession PRJNA742509) was collected from single grafted trees grown in the experimental field of the Julius Kühn Institute (JKI) – Federal Research Centre for Cultivated Plants, Institute for Breeding Research on Fruit Crops, Dresden, Germany (Figure 1, coordinates 50.996389 N 13.885465 E).

Two pools were generated for RNA extraction (pool 1: mature leaves, premature leaves, flower buds, vegetative buds and pool 2: open flower after pollination, green fruits, fruits in color change from green to red).

1.2 De novo assembly and scaffolding

To provide compatibility with Phase Genomics Hi-C scaffolding pipeline, separation of the ancestral genomes was performed by mapping publicly available *Prunus avium* reads (DRA004760, DRA004761, DRA004762, DRA004763, DRA004764, DRA004765, DRA004768, DRA004769, DRA004770, DRA004771, DRA004772) to the preliminary assembly with BWA-Mem (Li and Durbin 2009, Li and Durbin 2010). A combination of external python packages (scipy linregress, scipy find_peaks) were used to select contigs that fit the hypothesis of 1 or more clear coverage peaks on all *Prunus avium* derived contigs. The remaining contigs were then assigned to *Prunus fruticosa*. To test whether the separation was successful, the two subgenomes *Pce*s_a and *Pce*s_f were purged using the purge_dups (V1.0.1).

1.3 Correctness, completeness and contiguity of the Prunus cerasus genome sequence

K-mer analysis was performed due to the following steps: adapter sequences of both short-read sequence data sets (125 bp and 150bp paired end) were trimmed with Trimmomatic software (Bolger et al. 2014) and the trimmed data were used to estimate the best k-mer size using the genomic k-mer counting software Meryl (Galaxy Version 1.3+galaxy2, Rhie 2020): count the occurrence of canonical k-mers | estimate the best k-mer size | 300 Mb | 0.001. A database file was processed for each data set. Using the union-sum function, the resulting database files were merged.

1.4 Structural and functional annotation

1.4.1 Data preparation

Repeat masking for structural genome annotation was performed with RepeatModeler2 using the following dependency software versions: rmblast 2.11.0+, TRF 4.09 (Benson, 1999), RECON (Bao and Eddy, 2002), RepeatScout 1.0.6, RepeatMasker 4.1.2, LTR Structural Analysis: Enabled (GenomeTools 1.6.2 (Gremme et al., 2013), LTR_Retriever (Ou and Jiang, 2018) v2.9.0, Ninja (Wheeler, 2009) 0.95-cluster_only, MAFFT (Katoh and Standley, 2013) 7.487, CD-HIT (Fu et al, 2012) 4.8.1).

1.4.2 Long read integration

A primary gene set (transcriptome.gff) was generated with Cupcake.

Coverage information of PacBio transcripts is by default stored in the header of the transcript FASTA file. Genome annotation pipelines have not yet been adapted to use the information this way, but they require coverage information for adequate data incorporation. Therefore, we created coverage-equivalent redundant copies of transcripts with a coverage > 1 using a custom script explode_pacbio_ccs.pl (availabe at https://github.com/Gaius-Augustus/BRAKER/blob/long_reads/scripts/explode_pacbio_ccs.pl) as follows:

```
cat longreads.fastq | ./explode_pacbio_ccs.pl > exploded.fq
```

This file was spliced-aligned to the genome using Minimap2 version 2.17-r941 (Li, 2018). The resuling SAM file was converted to BAM format using SAMtools (Li et al., 2009). The resulting BAM file was provided to BRAKER1 (Hoff et al., 2016; Hoff et al., 2019) as input.

In addition, the Cupcake transcripts were processed as follows: The script stringtie2fa.py (available at https://github.com/Gaius-Augustus/Augustus/blob/master/scripts/stringtie2fa.py) was used to convert the Cupcake GTF-file into transcripts with the following command line:

```
stringtie2fa.py -g genome.chr.fa.masked -f transcriptome.chr.gff ¥     -o cupcake.fa
```

GeneMarkS-T version 5.1 March 2014 was executed to find CDS in transcript fasta file as follows:

```
gmst.pl --strand direct cupcake.fa.mrna --output gmst.out ¥
        --format GFF
```

The local CDS coordinates were projected to the genome with another custom script (available at https://github.com/Gaius-Augustus/BRAKER/blob/long_reads/scripts/gmst2globalCoords.py):

```
gmst2globalCoords.py -t transcriptome.chr.gff -p gmst.out ¥
        -o gmst.global.gtf -g genome.chr.fa.masked
```

The global coordinate GTF-File was converted to Hints for Augustus with another custom script contained in folder scripts (availble at https://github.com/Gaius-

Augustus/BRAKER/blob/long_reads/scripts/gmst_global2hints.pl), the source key is M, i.e. the prediction of these hints will be enforced in AUGUSTUS:
gmst_global2hints.pl gmst.global.gtf > pacbio.hints

1.4.3. Combination of BRAKER gene sets with TSEBRA

BRAKER1, BRAKER2, and long read gene sets were combined with a modified version of the TSEBRA combiner tool (modified version available at https://github.com/Gaius-Augustus/TSEBRA/tree/long_reads) using a custom configuration file:

```
tsebra.py -g braker1/augustus.hints.gtf,braker2/augustus.hints.gtf ¥   -e
braker1/hintsfile.gff,braker2/hintsfile.gff ¥
       -l gmst.global.gtf -c long_reads.cfg -o tsebra.gtf
```

Content of the custom TSEBRA configuration file (long_reads.cfg):

```
# Weight for each hint source
# Values have to be >= 0
P 31
E 0.150
C 15
M 100.5
L 0.5
# Required fraction of supported introns or supported start/stop-codons for a transcript
# Values have to be in [0,1]
intron_support 10.8
stopstasto_support 1
start_support 2
# Allowed difference for each feature
# Values have to be in [0,1]
e_1 0.1
e_2 0.51
e_3 1
# Values have to be >0
e_34 25300
e_54 1050
```

1.4.4. Gene structure prediction using GeMoMa

Homology-based gene annotation was performed with GeMoMa version 1.9 (Keilwagen et al. 2019) using the mapped RNA-seq data from *P. cerasus* cv 'Schattenmorelle' and the genome and gene annotation from the following reference organisms that are available at NCBI: *Arabidopsis thaliana* (TAIR10.1, RefSeq GCF_000001735.4), *Vitis vinifera* (12x, RefSeq GCF_000003745.3), *Populus trichocarpa* (Pop_tri_v3, GCF_000002775.4).

Other references were downloaded from the GDR database (www. https://www.rosaceae.org): *M. domestica* (*Malus* x *domestica* HFTH1 Whole Genome v1.0), *F. vesca* (*Fragaria vesca* Whole Genome v4.0.a1), *P. avium* (*Prunus avium* Tieton Genome v2.0), *P. persica* (*Prunus persica* Whole Genome Assembly v2.0, v2.0.a1), *P. dulcis* (*Prunus dulcis* Lauranne Genome v1.0) and *P. armeniaca* (*Prunus armeniaca* Marouch n14 Whole Genome v1.0), *P. yedonensis* (*Prunus yedoensis* var. *nudiflora* Genome v1.0), *P. domestica* (*Prunus domestica* Draft Genome Assembly v1.0), *P. communis* (*Pyrus communis* Bartlett DH Genome v2.0), *R. occidentalis* (*Rubus occidentalis* Whole Genome v3.0). *P. fruticosa* was downloaded from OpenAgrar (Wöhner et al. 2021 b).

1.4.5 Final gene structure generation

BUSCO version 5.2.2 with set embryophyta_odb10 (number of genomes: 50, number of BUSCOs: 1614) was used for the assessment of protein completeness. For handling alternative transcripts correctly and not as duplicates, a custom script was ran on the BUSCO full table, assigning gene ID instead of transcript ID.

The chloroplast and mitochondria sequences were annotated with GeSeq (Tillich et al. 2017) using the all available references for chloroplast (*P. armenica, P. avium, P. campanulata, P. cerasoides, P. davidiana, P. dictyoneura, P. domestica, P. dulcis, P. humilis, P. kansuensis, P. matuurae, P. maximowiczii, P. mira, P. mongolica, P. mume, P. penduculata, P. persica, P. pseudocerasus, P. rufa, P. salicina, P. serotine, P. speciosa, P. takesimensis, P. tenella, P. tomentosa, P. triloba, P. yedonensis, P. zippeliana*) from NCBI and mitochondria from *P. avium* (GenBank accession MK816392) published by Yan et al. (2019). GeSeq pipeline analysis was performed using the annotation packages ARAGORN, blatN, blatX, Chloe and HMMER.

1.4.6 Protein clustering, multiple sequence alignment and divergence of time estimation

The Proteinortho (Galaxy Version 6.0.32+galaxy0) was used to find orthologous proteins within the datasets with the following parameters: LAST, e-value treshold = 0.001, minimal algebraic connectivity = 0.1, in add. options: Minimal coverage of best alignment in % = 50, min. seq. similarity in % = 95, minimal percent identity of best blast hits in % = 25.

MAFFT (Galaxy Version 7.505+galaxy0, Katoh and Standley 2013) was used to align each obtained orthogroup with the following parameters: gap extent 0.123, gap open 1.53, no matrix, output fasta.

A timetree was inferred by applying the RelTime method (Tamura et al. 2012, Tamura et al. 2018) to the user-supplied phylogenetic tree whose branch lengths were calculated using the Ordinary Least Squares method. The timetree was computed using 1 calibration constraint. The Tao et al. (2020) method was used to set minimum and maximum time boundaries on nodes for which calibration densities were provided. Confidence intervals were computed using the Tao et al. (2020) method. The evolutionary distances were computed using the JTT matrix-based method (Jones et al. 1992) and are in the units of the number of amino acid substitutions per site. The rate variation among sites was modelled with a gamma distribution (shape parameter = 1). This analysis involved nine amino acid sequences. There were 419,586 positions in the final dataset.

References

Kim, D., Paggi, J. M., Park, C., Bennett, C., & Salzberg, S. L. (2019). Graph-based genome alignment and genotyping with HISAT2 and HISAT-genotype. Nature biotechnology, 37(8), 907-915.

Li, H., & Durbin, R. (2009). Fast and accurate short read alignment with Burrows-Wheeler transform. Bioinformatics, 25(14), 1754–1760. https://doi.org/10.1093/bioinformatics/btp324

Li, H., & Durbin, R. (2010). Fast and accurate long-read alignment with Burrows–Wheeler transform. Bioinformatics, 26(5), 589–595. https://doi.org/10.1093/bioinformatics/btp698

Benson, G. (1999). Tandem repeats finder: a program to analyze DNA sequences. Nucleic acids research, 27(2), 573-580

Bao, Z., & Eddy, S. R. (2002). Automated de novo identification of repeat sequence families in sequenced genomes. Genome research, 12(8), 1269-1276.

Gremme, G., Steinbiss, S., & Kurtz, S. (2013). GenomeTools: a comprehensive software library for efficient processing of structured genome annotations. IEEE/ACM transactions on computational biology and bioinformatics, 10(3), 645-656.

Ou, S., & Jiang, N. (2018). LTR_retriever: a highly accurate and sensitive program for identification of long terminal repeat retrotransposons. Plant physiology, 176(2), 1410-1422.

Wheeler, T. J. (2009, September). Large-scale neighbor-joining with NINJA. In International Workshop on Algorithms in Bioinformatics (pp. 375-389). Springer, Berlin, Heidelberg.

Katoh, K., & Standley, D. M. (2013). MAFFT multiple sequence alignment software version 7: improvements in performance and usability. Molecular biology and evolution, 30(4), 772-780.

Fu, L., Niu, B., Zhu, Z., Wu, S., & Li, W. (2012). CD-HIT: accelerated for clustering the next-generation sequencing data. Bioinformatics, 28(23), 3150-3152.

Li, H. (2018). Minimap2: pairwise alignment for nucleotide sequences. Bioinformatics, 34(18), 3094-3100.

Li, H., Handsaker, B., Wysoker, A., Fennell, T., Ruan, J., Homer, N., ... & Durbin, R. (2009). The sequence alignment/map format and SAMtools. Bioinformatics, 25(16), 2078-2079.

Hoff, K. J., Lange, S., Lomsadze, A., Borodovsky, M., & Stanke, M. (2016). BRAKER1: unsupervised RNA-Seq-based genome annotation with GeneMark-ET and AUGUSTUS. Bioinformatics, 32(5), 767-769.

Hoff, K. J., Lomsadze, A., Borodovsky, M., & Stanke, M. (2019). Whole-genome annotation with BRAKER. In Gene Prediction (pp. 65-95). Humana, New York, NY

J. Keilwagen, F. Hartung, J. Grau, GeMoMa: Homology-based gene prediction utilizing intron position conservation and RNA-seq data, in: Gene Prediction, Springer, 2019, pp. 161–177.

M. Tillich, P. Lehwark, T. Pellizzer, E.S. Ulbricht-Jones, A. Fischer, R. Bock, S. Greiner, GeSeq–versatile and accurate annotation of organelle genomes, Nucleic acids research 45 (2017) W6-W11.

M. Yan, X. Zhang, X. Zhao, Z. Yuan, The complete mitochondrial genome sequence of sweet cherry (*Prunus avium* cv.'summit'), Mitochondrial DNA Part B 4 (2019) 1996–1997.

Katoh, K., & Standley, D. M. (2013). MAFFT Multiple Sequence Alignment Software Version 7: Improvements in Performance and Usability. *Molecular Biology and Evolution*, *30*(4), 772–780. https://doi.org/10.1093/molbev/mst010

Tamura K., Battistuzzi FU, Billing-Ross P, Murillo O, Filipski A, and Kumar S. (**2012**). Estimating Divergence Times in Large Molecular Phylogenies. *Proceedings of the National Academy of Sciences* **109**:19333-19338.

Tamura K., Qiqing T., and Kumar S. (**2018**). Theoretical Foundation of the RelTime Method for Estimating Divergence Times from Variable Evolutionary Rates. *Molecular Biology and Evolution* **35**: 1770-1782.

Tao Q., Tamura K,. Mello B., and Kumar S. (**2020**) Reliable Confidence Intervals for RelTime Estimates of Evolutionary Divergence Times. *Molecular Biology and Evolution, 37(1): 280-290*

Wöhner, T. W., Emeriewen, O. F., Wittenberg, A. H., Schneiders, H., Vrijenhoek, I., Halász, J., ... & Flachowsky, H. (2021a). The draft chromosome-level genome assembly of tetraploid ground cherry (Prunus fruticosa Pall.) from long reads. *Genomics*, *113*(6), 4173-4183.

T.W. Wöhner, O.F. Emeriewen, A.H.J. Wittenberg, H. Schneiders, I. Vrijenhoek, J. Halász, K. Hrotkó, K.J. Hoff, L. Gabriel, J. Keilwagen, T. Berner, M. Schuster, A. Peil, J. Wünsche, S. Kropop, H. Flachowsky (2021b). Supporting Materials for - The Draft Chromosome-level Genome Assembly of Tetraploid Ground Cherry (*Prunus fruticosa* Pall.) from Long Reads. https://www.openagrar.de/receive/openagrar_mods_00070329 (2021) (accessed 1 June 2021)

Jones D.T., Taylor W.R., and Thornton J.M. (1992). The rapid generation of mutation data matrices from protein sequences. Computer Applications in the Biosciences 8: 275-282.

Bolger AM, Lohse M, Usadel B. Trimmomatic: a flexible trimmer for Illumina sequence data. Bioinformatics 2014;30(15):2114–20. https://doi.org/10.1093/bioinformatics/btu170.

Arang Rhie (2020). Meryl. In GitHub repository. GitHub. https://github.com/marbl/meryl.

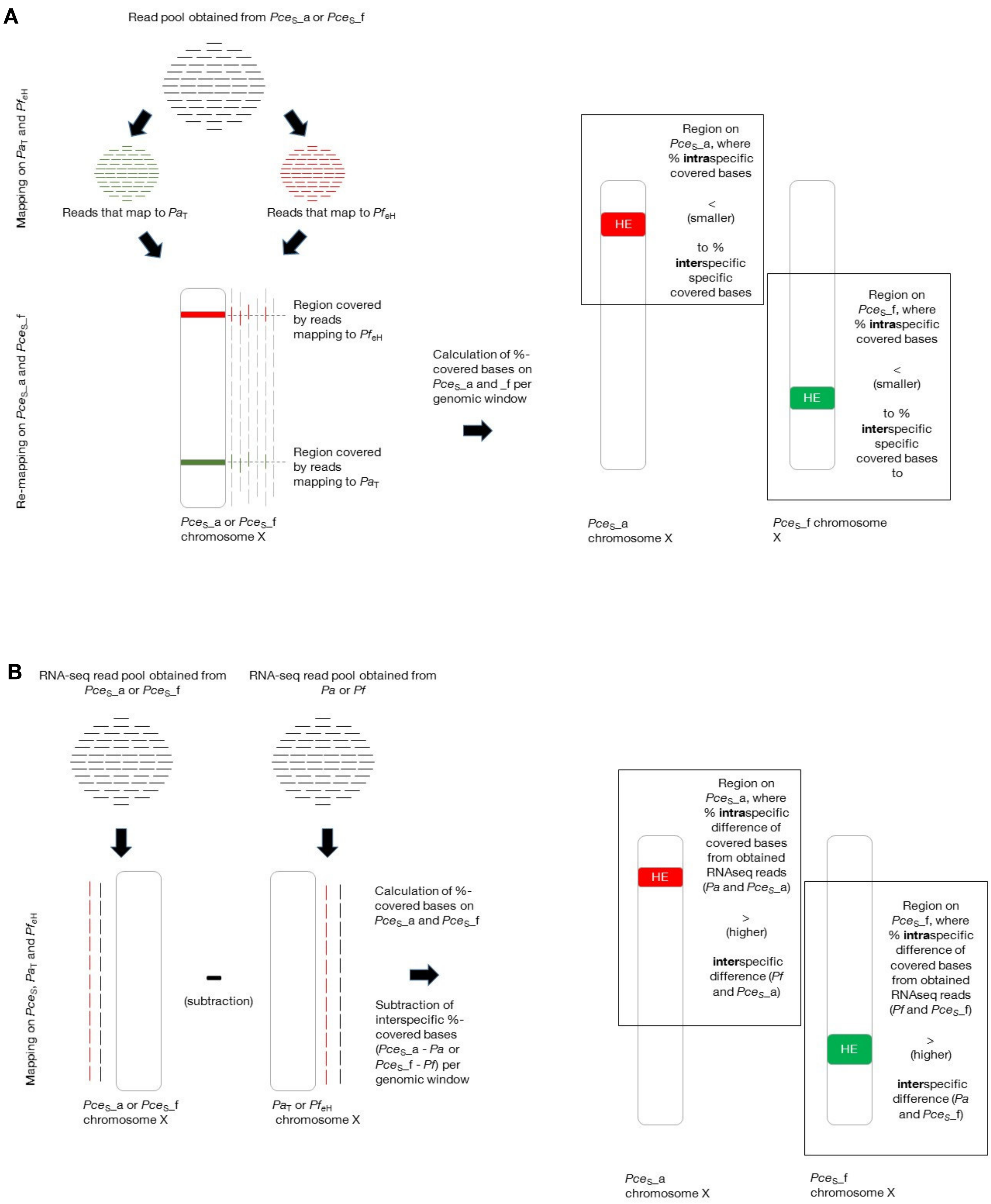

A
Read pool obtained from Pce_S_a or Pce_S_f
Mapping on Pa_T and Pf_eH
Reads that map to Pa_T
Reads that map to Pf_eH
Re-mapping on Pce_S_a and Pce_S_f
Region covered by reads mapping to Pf_eH
Region covered by reads mapping to Pa_T
Pce_S_a or Pce_S_f chromosome X
Calculation of %-covered bases on Pce_S_a and _f per genomic window
Region on Pce_S_a, where % **intra**specific covered bases
<
(smaller)
to % **inter**specific specific covered bases
HE
Region on Pce_S_f, where % **intra**specific covered bases
<
(smaller)
to % **inter**specific specific covered bases to
HE
Pce_S_a chromosome X
Pce_S_f chromosome X
B
RNA-seq read pool obtained from Pce_S_a or Pce_S_f
RNA-seq read pool obtained from Pa or Pf
Mapping on Pce_S, Pa_T and Pf_eH
(subtraction)
Pce_S_a or Pce_S_f chromosome X
Pa_T or Pf_eH chromosome X
Calculation of %-covered bases on Pce_S_a and Pce_S_f
Subtraction of interspecific %-covered bases (Pce_S_a - Pa or Pce_S_f - Pf) per genomic window
Region on Pce_S_a, where % **intra**specific difference of covered bases from obtained RNAseq reads (Pa and Pce_S_a)
>
(higher)
interspecific difference (Pf and Pce_S_a)
HE
Region on Pce_S_f, where % **intra**specific difference of covered bases from obtained RNAseq reads (Pf and Pce_S_f)
>
(higher)
interspecific difference (Pa and Pce_S_f)
HE
Pce_S_a chromosome X
Pce_S_f chromosome X

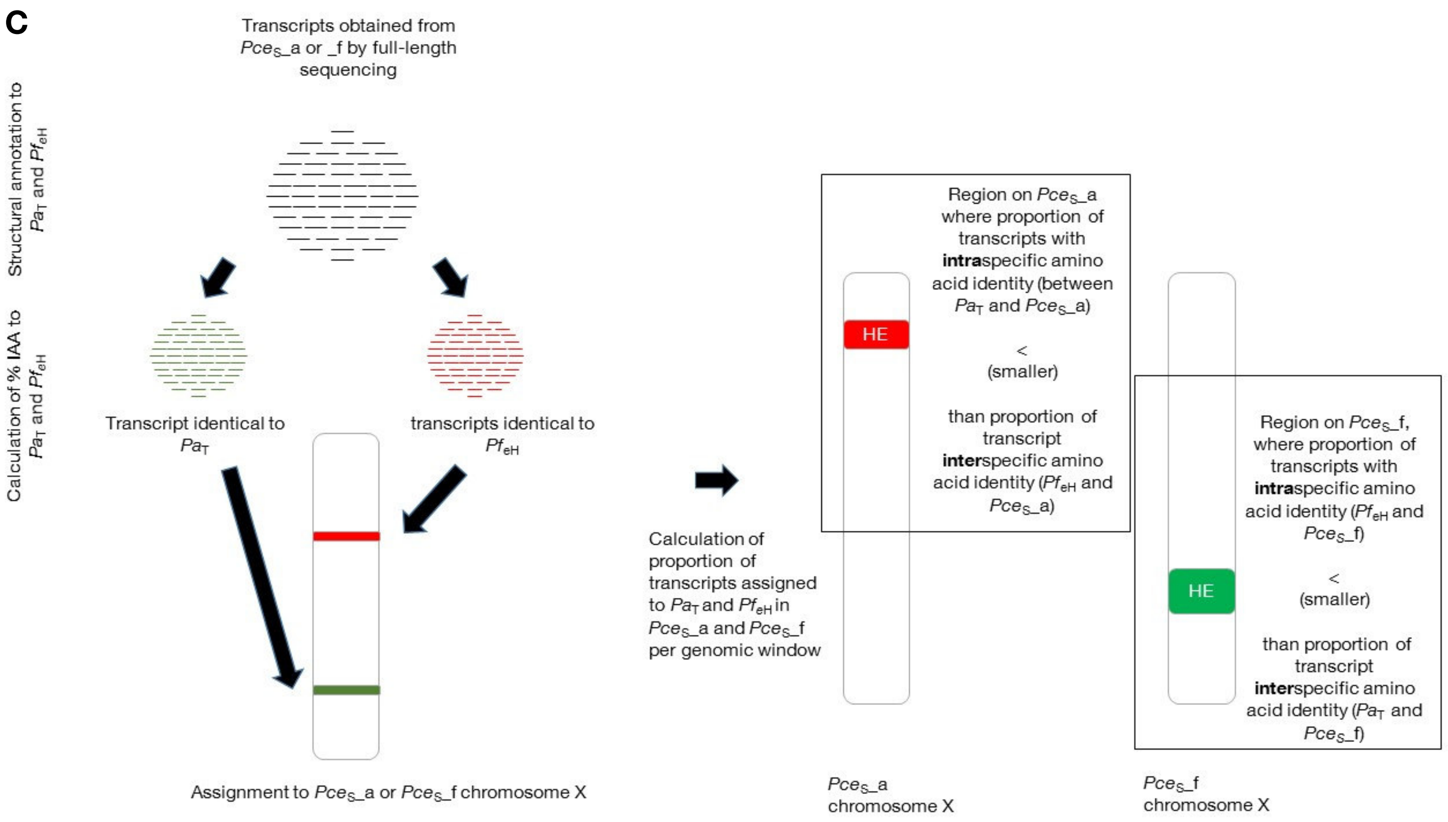

Figure S1 Schematic summary of the 3 approaches to evaluate homoelogous exchanges (HE) in the *P. cerasus* L. genome sequence of 'Schattenmorelle' by (**A**) genomic reads: the intraspecific % of covered bases from mapped reads (Pce_S_a to Pa_T, Pce_S_f to Pf_{eH}) is smaller (<) than the interspecific % of covered bases from mapped reads (Pce_S_a to Pf_{eH}, Pce_S_f to Pa_T); (**B**) transcriptomic reads: Intraspecific difference of covered bases from obtained RNAseq reads (% covered bases from *Pa* reads subtracted (-) from % covered bases from Pce_S_a, and vice versa for *Pf* and Pce_S_f) is higher (>) than the interspecific difference of covered bases from obtained RNAseq reads (% covered bases from *Pf* subtracted from Pce_S_a, and vice versa for *Pa* and Pce_S_f); (**C**) Identity of amino acids (IAA): were proportion of transcripts with intraspecific amino acid identity (Pa_T and Pce_S_a, Pf_{eH} and Pce_S_f) smaller (<) than the proportion of transcripts with interspecific amino acid identity (Pf_{eH} and Pce_S_a , Pa_T and Pce_S_f).

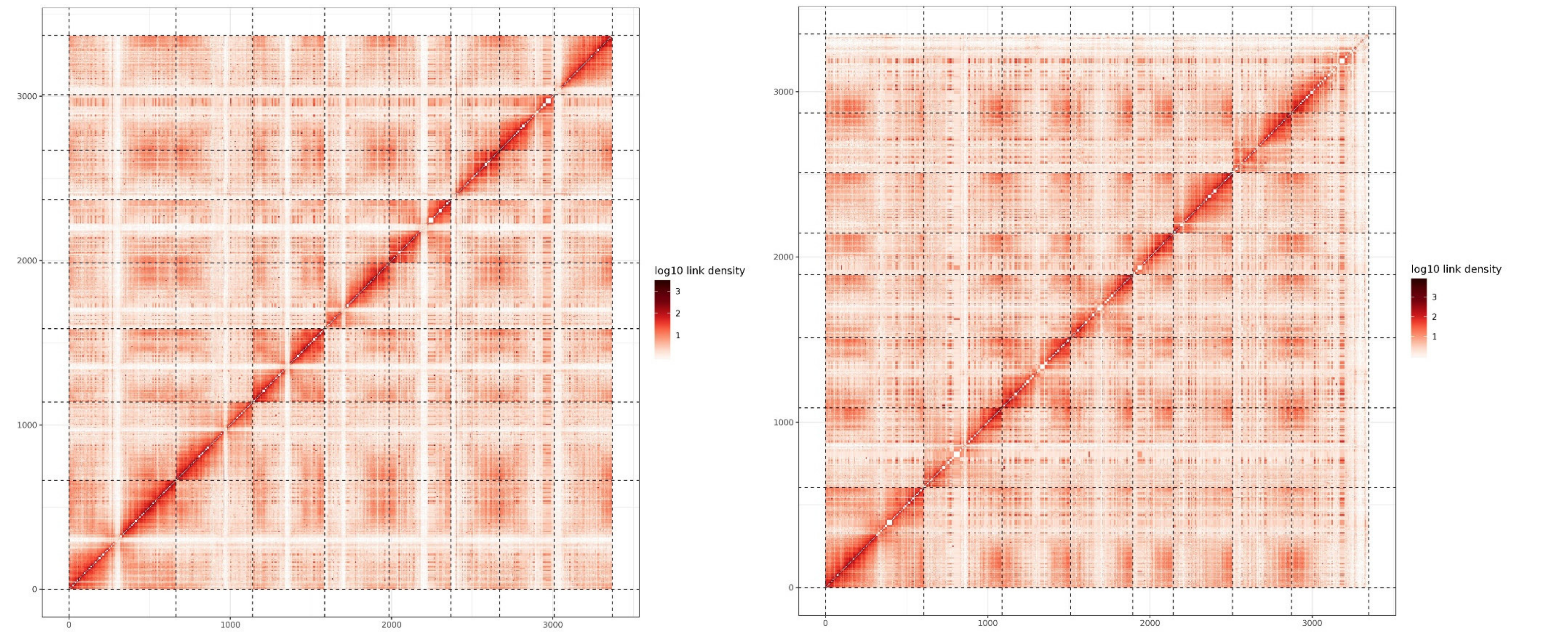

Figure S2 Hi-C heatmap post-scaffolding for the subgenomes *Pce*S_a (left) and *Pce*S_f (right) of *P. cerasus* cv 'Schattenmorelle'. The heatmap indicates the density of paired Hi-C reads which interact to each other in close proximity. High intense colour indicates high interaction.

(A)

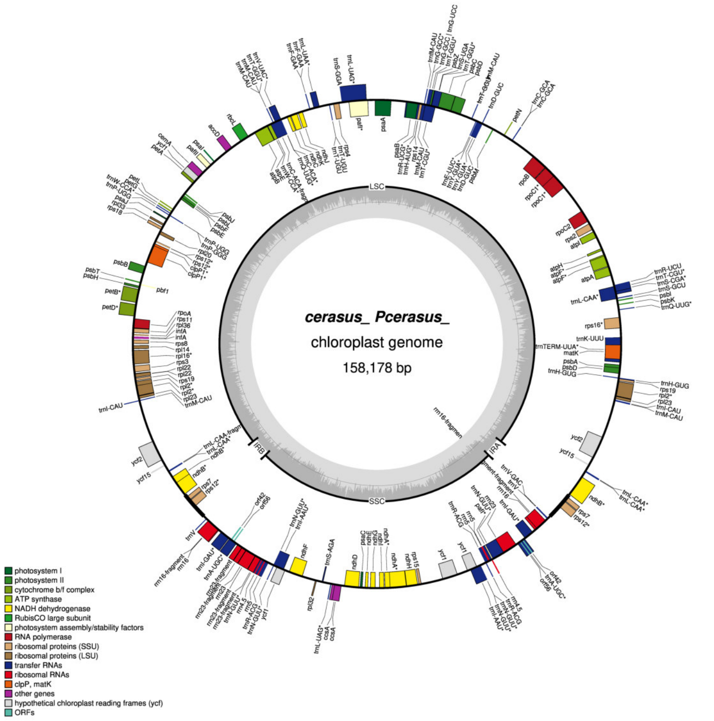

(B)

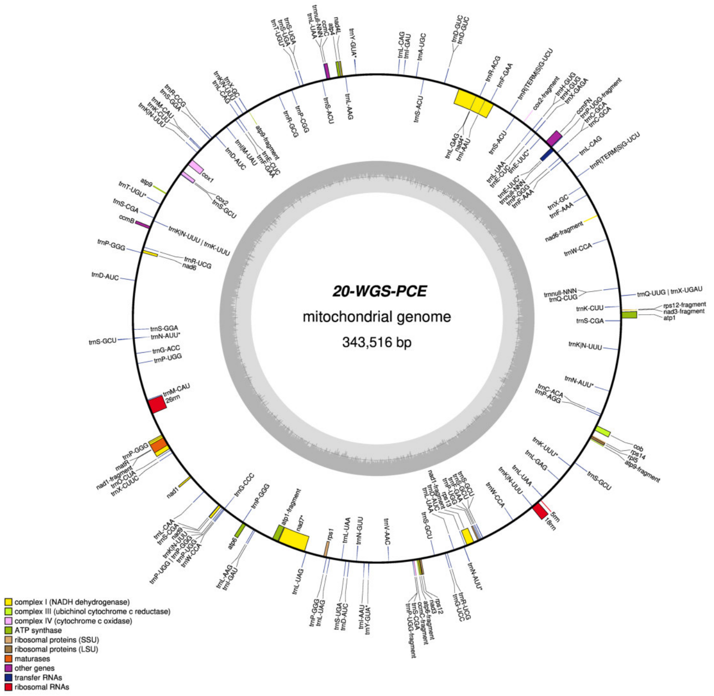

Figure S3 The chloroplast (a) and mitochondrial (b) sequences of *P. cerasus* L. cv 'Schattenmorelle'.

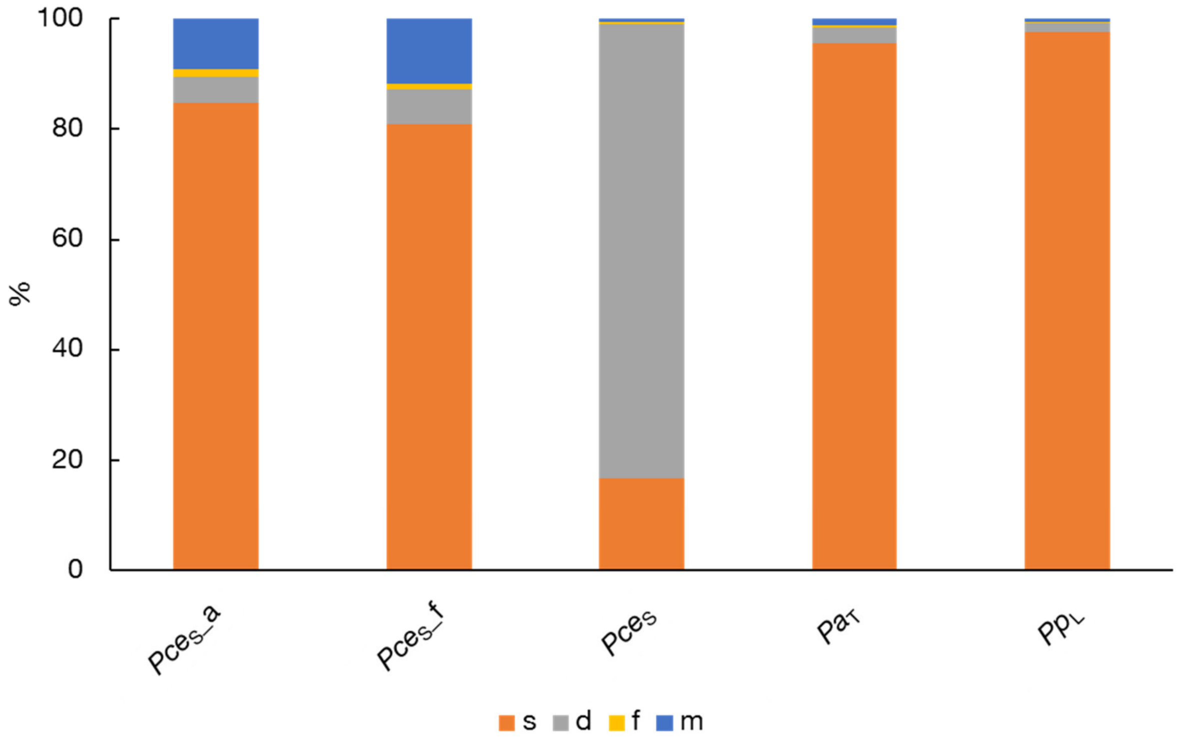

Figure S4 Analysis of completeness of the *P. cerasus* cv. Schattenmorelle subgenomes *P. cerasus* cv 'Schattenmorelle' subgenome *avium* (*Pce*S_a) and *P. cerasus* cv 'Schattenmorelle' subgenome *fruticosa* (*Pce*S_f) and combined datasets compared to *P. avium* cv. 'Tieton' (*Pa*T) and *P. persica* cv. Lovell (*Pp*L) by mapping of a set of universal single-copy orthologs using BUSCO. The bar charts indicate complete **s**ingle copy (orange), complete **d**uplicated (gray), **f**ragmented (yellow) and **m**issing (blue) genes. For evaluation the embryophyta_odb10 BUSCO dataset (n=1614) was used. *P. cerasus* cv. Schattenmorelle show a 99 % completeness (S: 16.7 %, D: 82.3 %, F: 0.4 %, M: 0.6 %, n: 1614) which reaches the completeness of *P. avium* cv. 'Tieton' (C: 98.3 %, S: 95.6 %, D: 2.7 %, F: 0.5 %, M:1.5 %, n:1614) and *P. persica* 'Lovell' (C: 99.3 %, S: 97.5 %, D: 1.8%, F: 0.1 %, M: 0.6 %, n:1614).

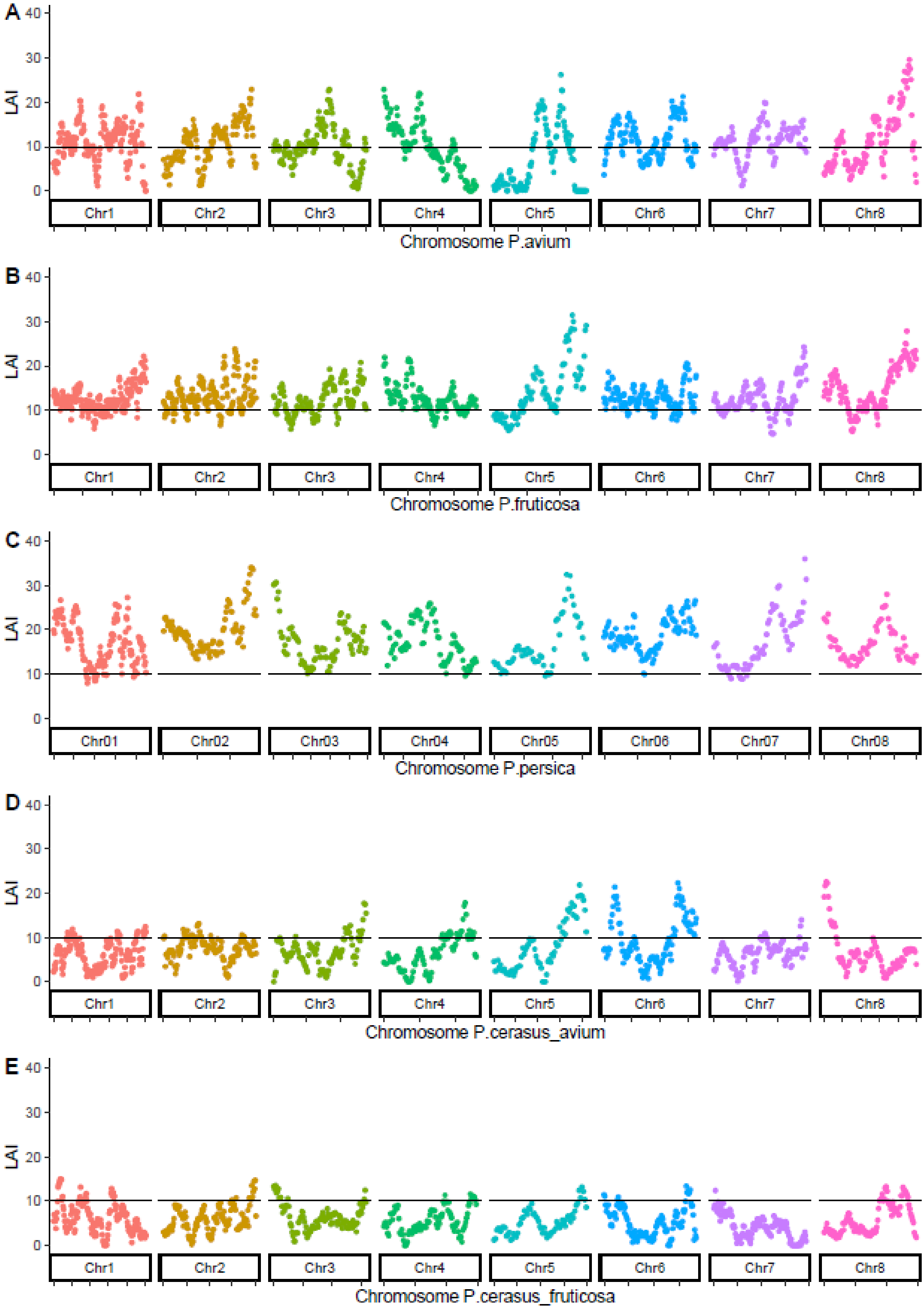

A
B
C
D
E
LAI
40
30
20
10
0
Chr1 Chr2 Chr3 Chr4 Chr5 Chr6 Chr7 Chr8
Chr01 Chr02 Chr03 Chr04 Chr05 Chr06 Chr07 Chr08
Chromosome P.avium
Chromosome P.fruticosa
Chromosome P.persica
Chromosome P.cerasus_avium
Chromosome P.cerasus_fruticosa

Figure S5 Assessing the quality of repetitive sequences between the chromosome sequences of *P. avium* 'Tieton' (A), *P. fruticosa* ecotype Hármashatárhegy (B), *P. perisca* 'Lovell', and (C) *P. cerasus* subgenome *avium* and *P. cerasus* subgenome *fruticosa* using the LAI index. The genomes *P. cerasus* [this study] and *P. avium* were sequenced with ONT 9.4.1 and Illumina (Wang wet al. 2020), *P. fruticosa* with ONT 10.3 (Wöhner et al. 2021) and *P. persica* with Illumina and Sanger sequencing of fosmid and BAC clones (Verde et al. 2017).

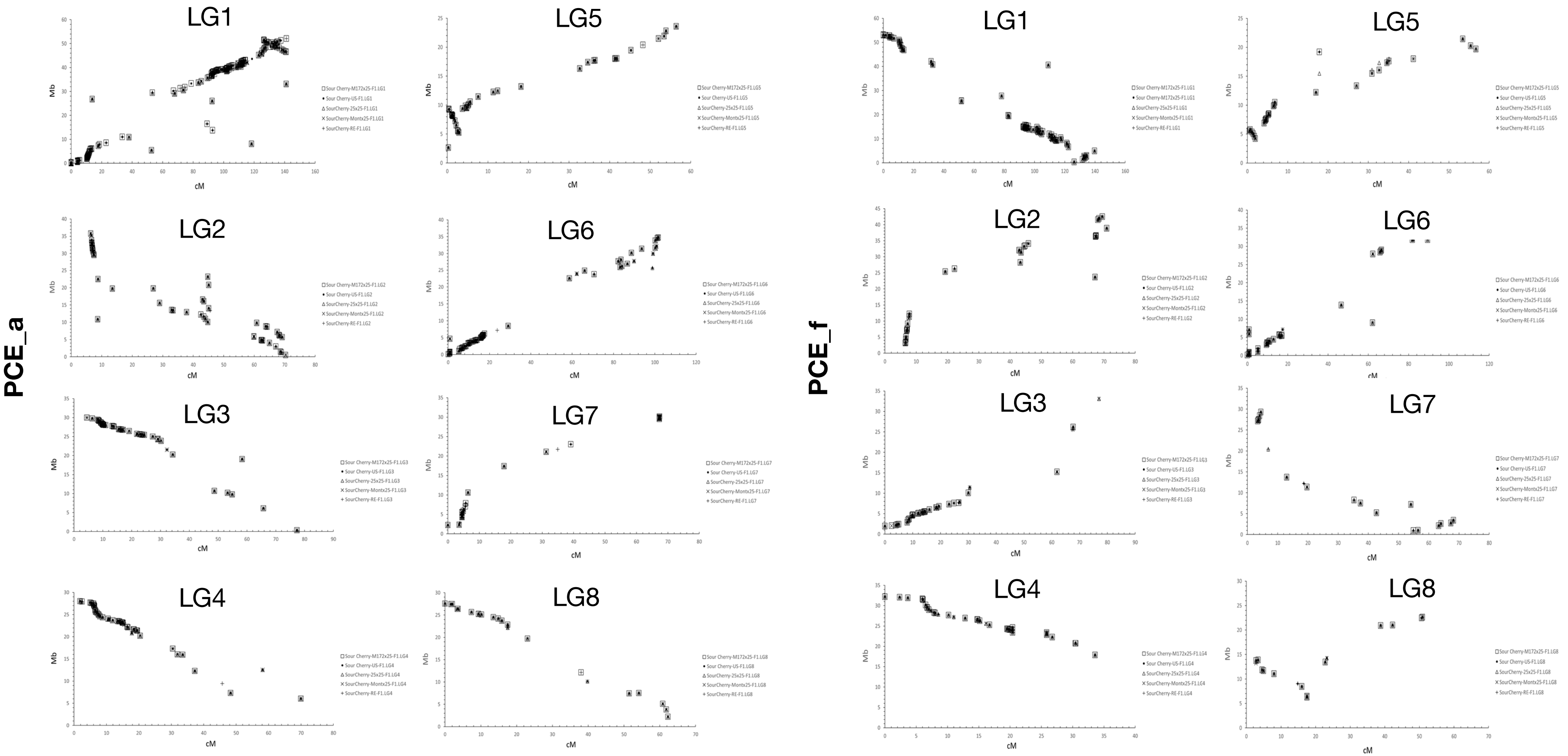

Figure S6 Collinearity plots between the five published genetic maps of sour cherry (M172x25-F1, US-F1, 25x25-F1, Montx25-F1, RE-F1) and the *P. cerasus* cv 'Schattenmorelle' subgenome *avium* (*Pce*S_a) and *fruticosa* (*Pce*S_f).X-axis represents the genetic position of a marker in the genetic linkage map given in centi Morgan (cM). Y-axis represents the physical position of a marker sequence within the genome sequence of the respective subgenome given in Mega base pairs (Mbp).

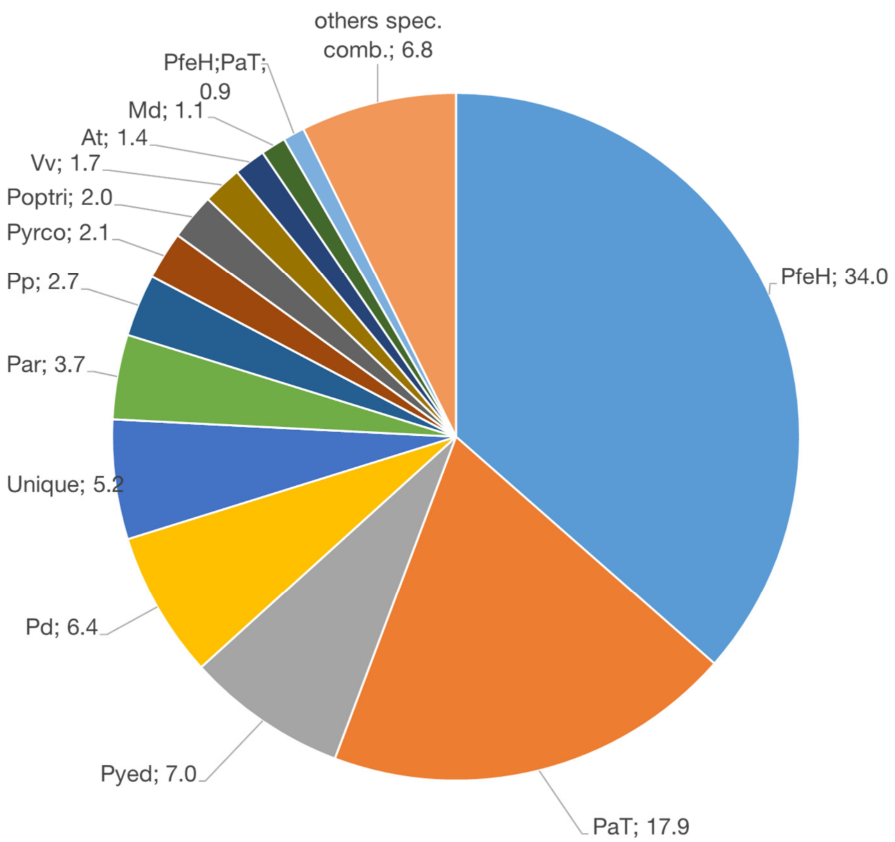

Figure S7 Percentage of *P. cerasus* (*Pce*) proteins by IAA compared with 15 reference species. *P. fruticosa* ecotype Hármashatárhegy (P_{feH}), *P. avium* 'Tieton' (Pa_{T}), *P. yedonensis* (*Pyed*), *P. domestica* (*Pd*), *P. armeniaca* (*Par*), *P. persica* (*Pp*), *Pyrus communis* (*Pyrco*), *Populus trichocarpa* (*Poptri*), *Vitis vinifera* (*Vv*), *Arabidopsis thaliana* (*At*), *Malus domestica* (*Md*).

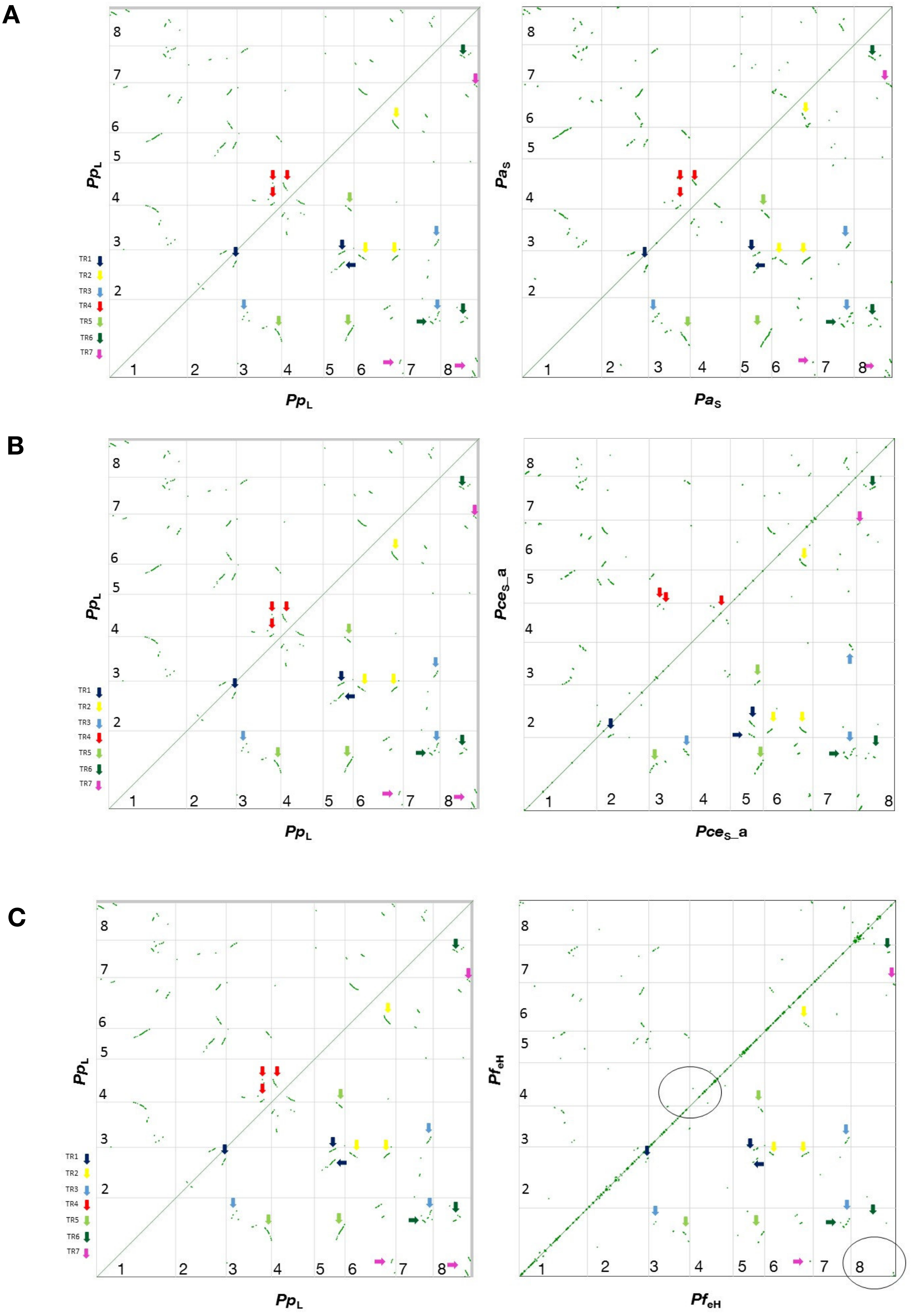
A
B
C
Pp_L
Pa_S
Pce_S_a
Pf_eH
TR1
TR2
TR3
TR4
TR5
TR6
TR7
1
2
3
4
5
6
7
8

D

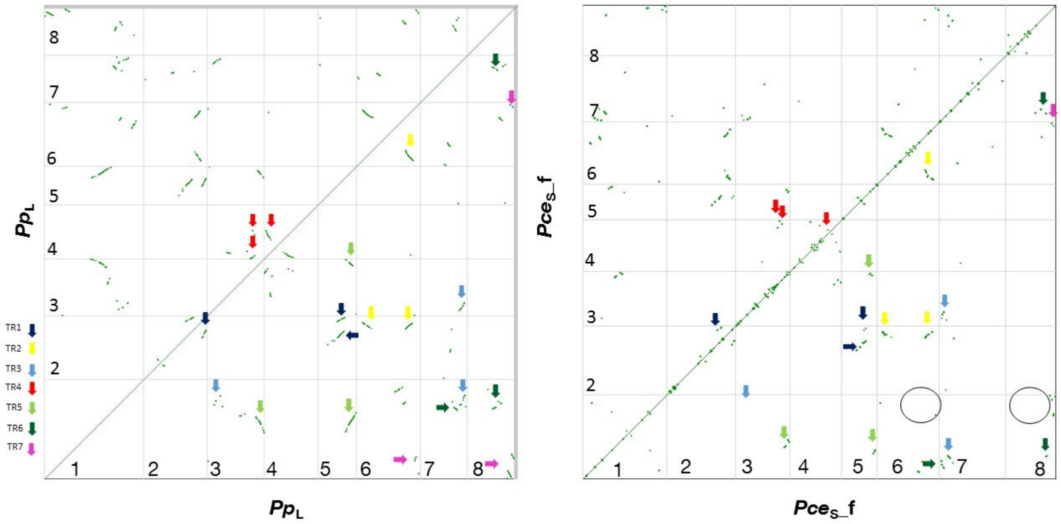

Figure S8 Synmap2 plots of self-self comparisons between (A) *Prunus persica* 'Lovell' (Pp_L) and *P. avium* 'Sato Nishiki' (Pa_S), (B) *P. fruticosa* 'Hármashatárhegy' (Pf_{eH}), (C) *P. cerasus_avium* 'Schattenmorelle' (Pce_S_a), (D) *P. cerasus_fruticosa* ‘Schattenmorelle’ (Pce_S_f) for the identification of triplicated regions (TR) 1-7.

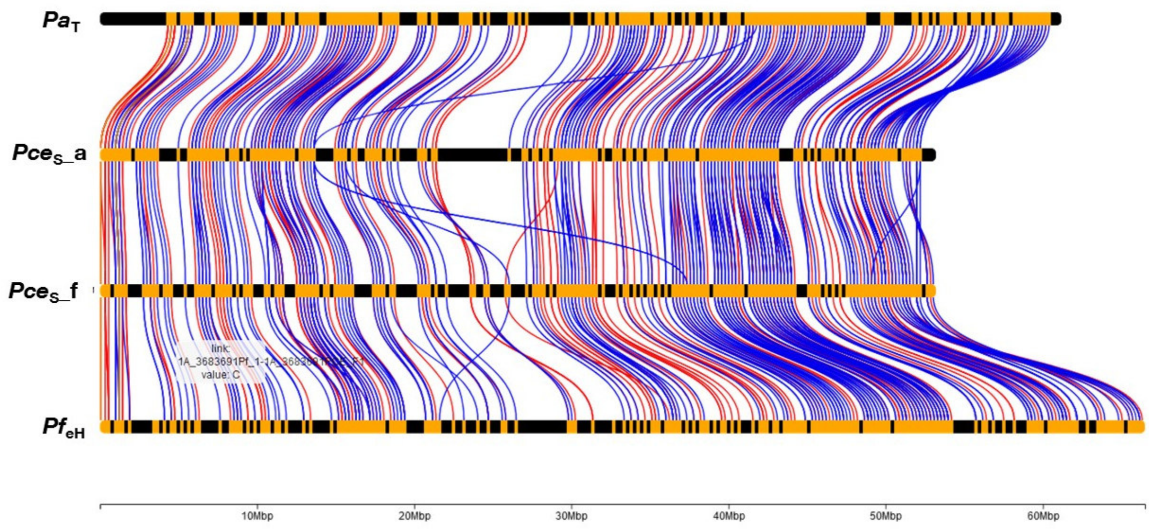

Chr1

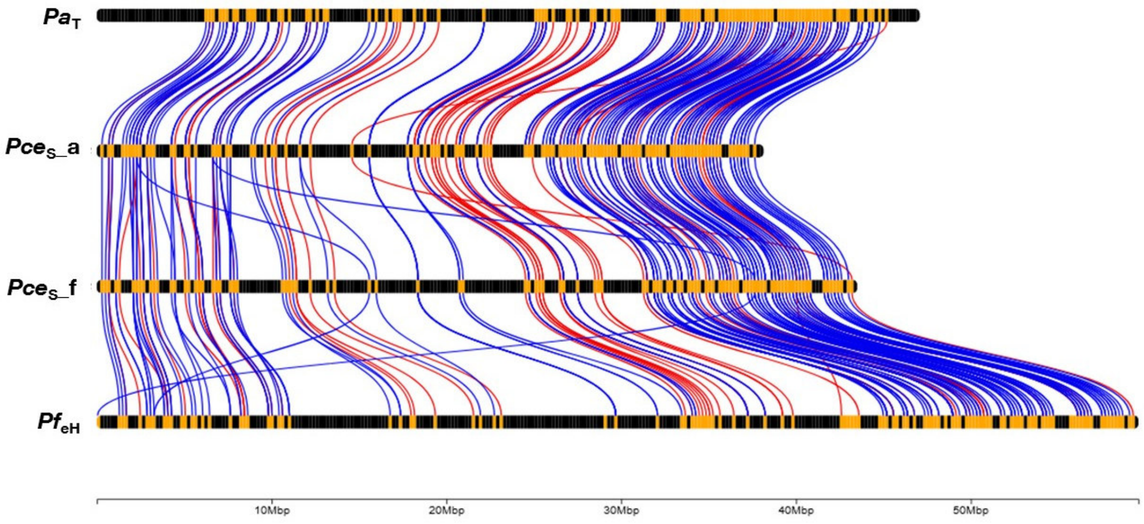

Chr2

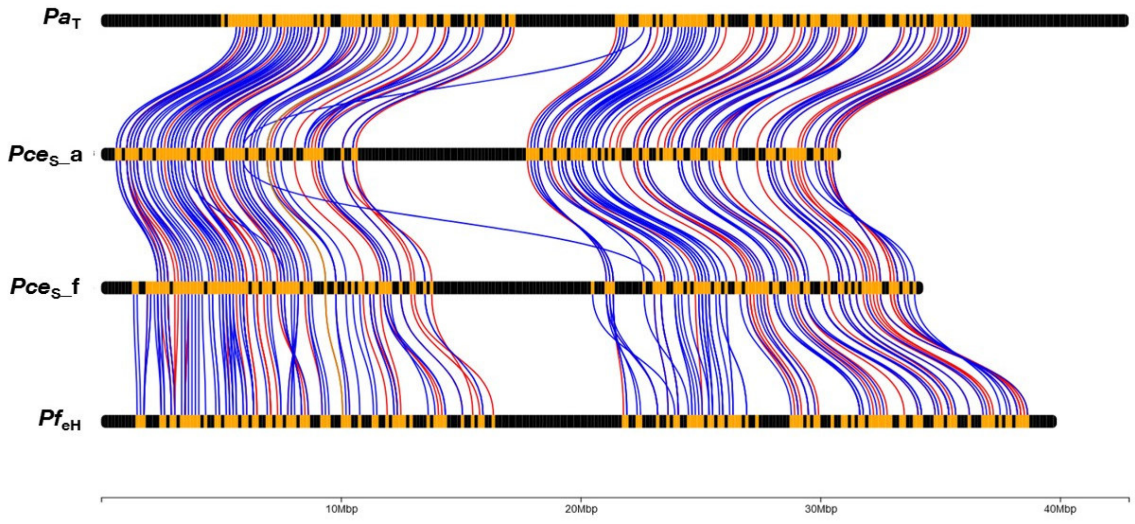

Chr3

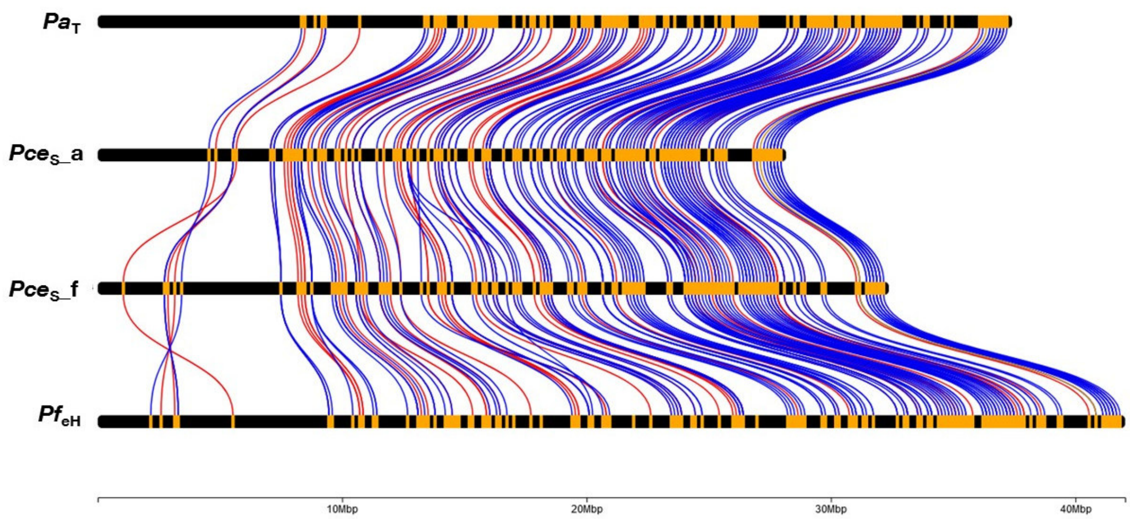

Chr4

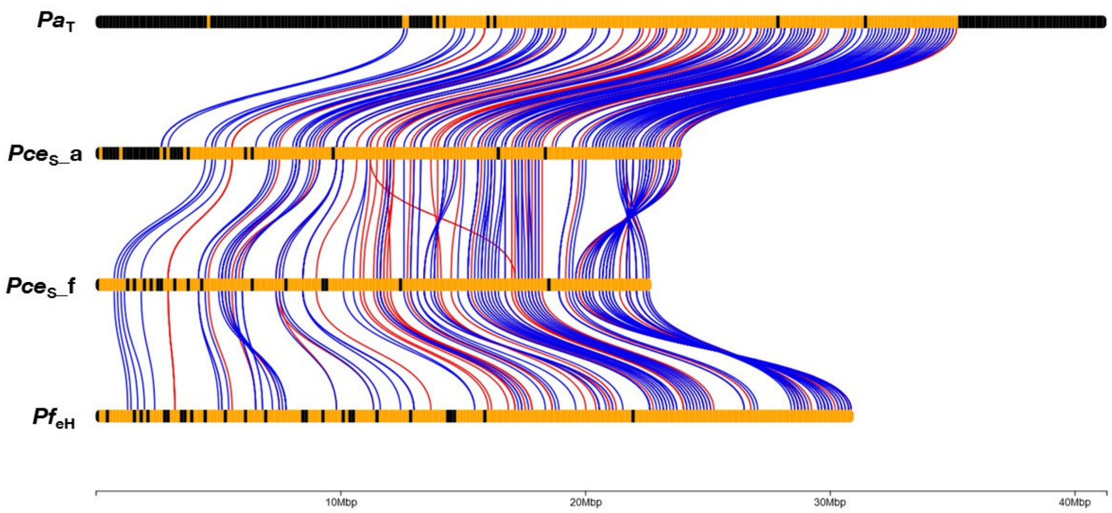

Chr5

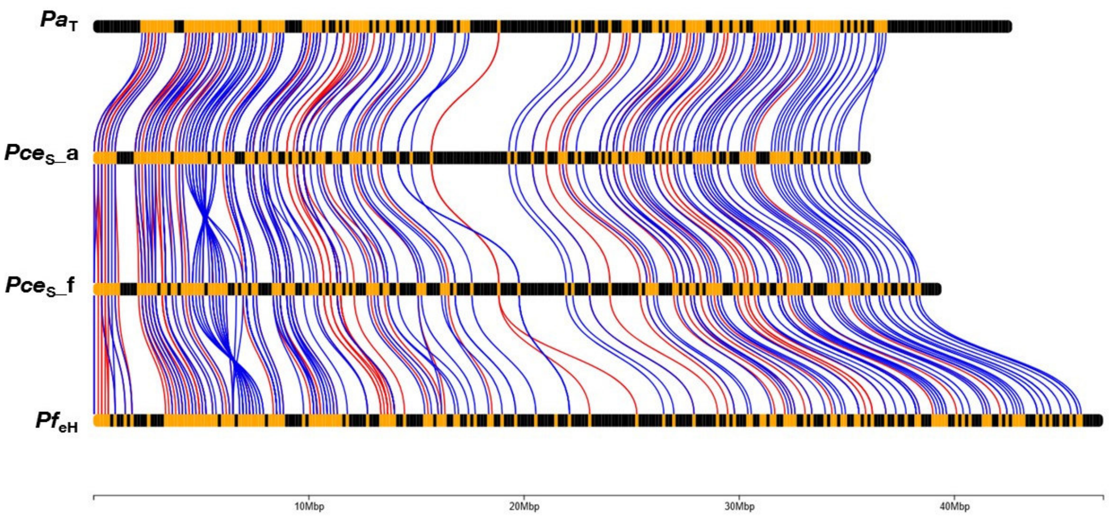

Chr6

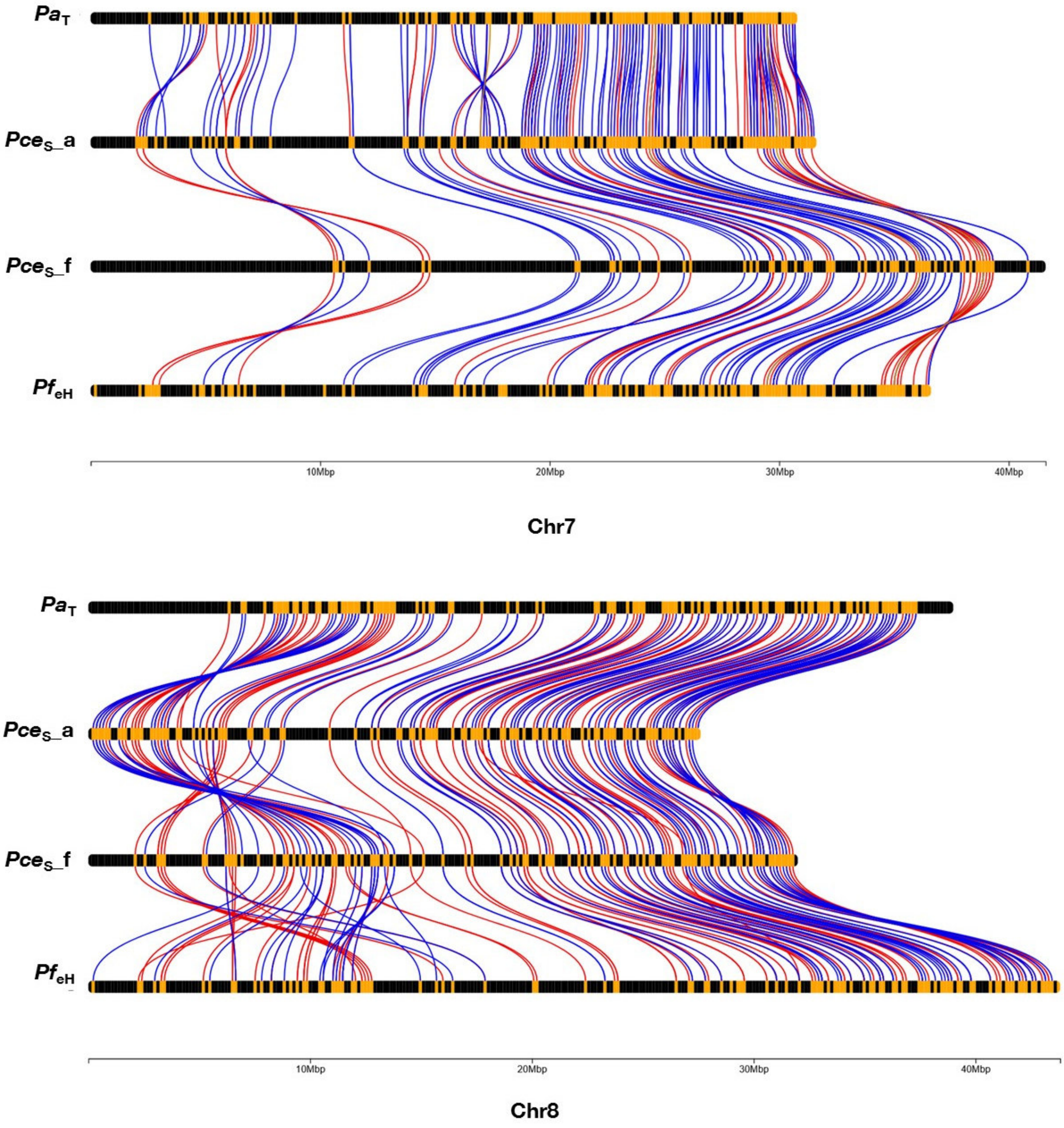

Figure S9 Positional co-linearity comparison between the two subgenomes *P. cerasus_avium* 'Schattenmorelle' (*Pce*$_{\mathrm{S}}$_a), *P. cerasus_fruticosa* ‘Schattenmorelle’ (*Pce*$_{\mathrm{S}}$_f) and *P. avium* 'Tieton' (*Pa*$_{\mathrm{T}}$), *P. fruticosa* 'Hármashatárhegy' (*Pf*$_{\mathrm{eH}}$), using the molecular markers from the 9+6k SNP array. The plots were generated using the R-software package chromoMap v0.4.1.

A

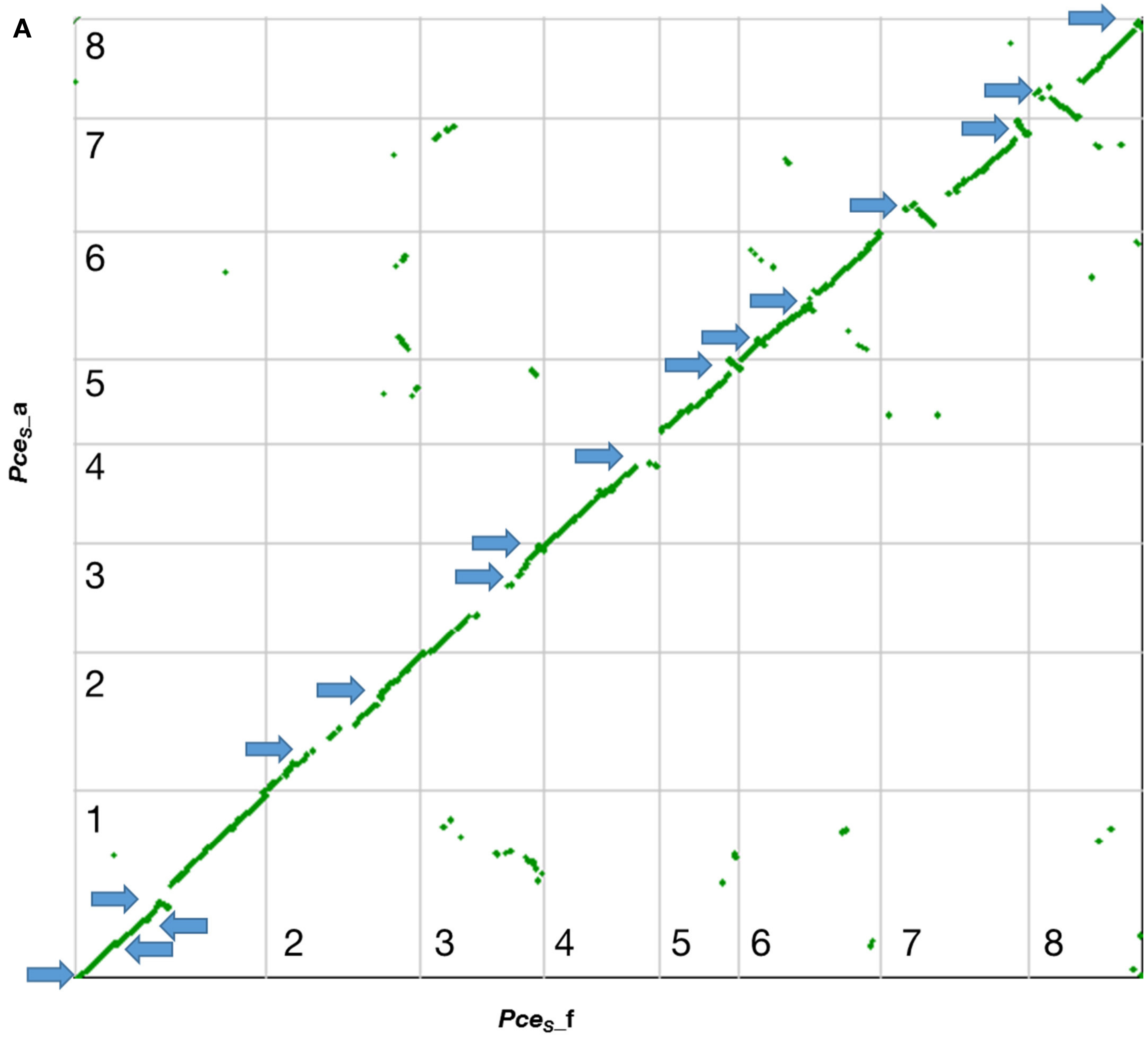

B

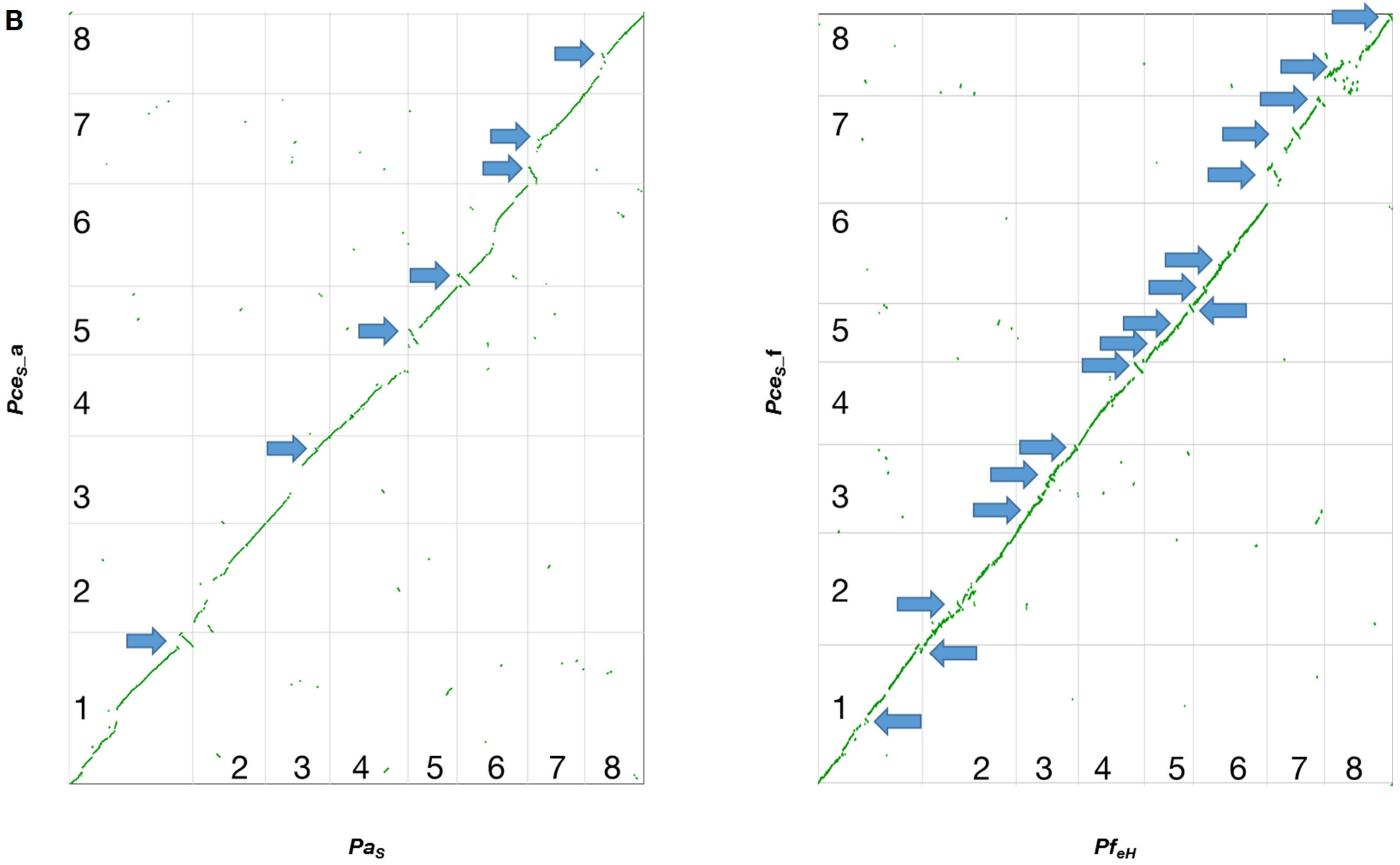

Figure S10 Synteny between (a) *P. cerasus* subgenome *_avium* (*Pce*S_a) and *P. cerasus* subgenome *_fruticosa* (*Pce*S_f), and (b) the subgenomes and the genotypes *Prunus avium* 'Tieton' (Pa_T) and *P. fruticosa* (Pf_{eH}) of the ancestral species *P. avium* and *P. fruticosa.* Blue arrows indicate positions where inversions occurred.

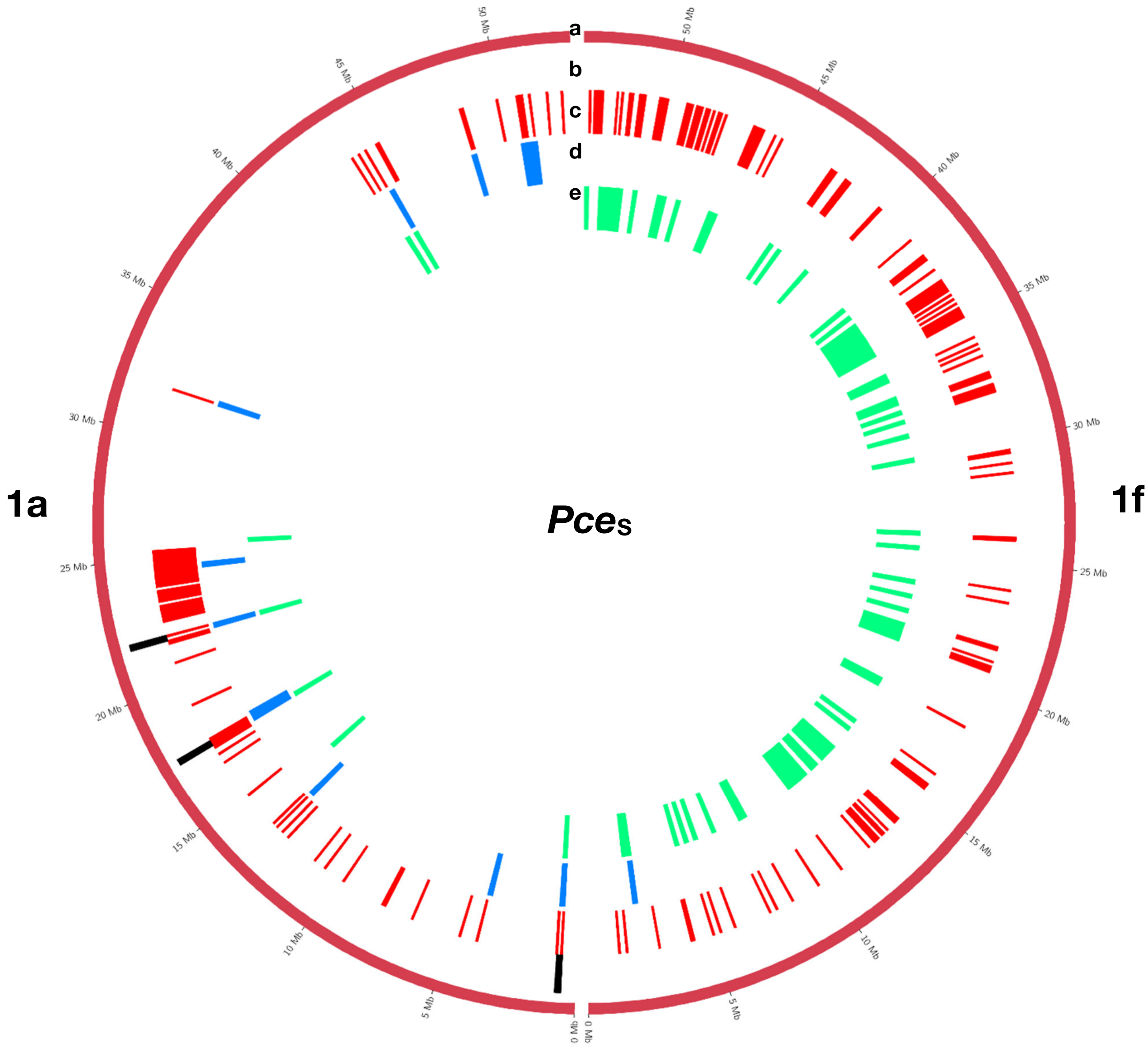

Figure S11 Detected regions of homoeologous exchanges in the genome of *P. cerasus* 'Schattenmorelle'. Circos plot of 16 single pseudomolecules 1 to 8 (see continuating plots) of the subgenomes of Pce_{S}_a and Pce_{S}_f. (a) chromosome length (Mb); (b) region in Pce_{S}_a and in Pce_{S}_f detected that match all three following analysis methods: (c) regions (100k window) were intraspecific %-covered bases from mapped reads (Pce_{S}_a to Pa_{T}, Pce_{S}_f to Pf_{eH}) was < than interspecific %-covered bases from mapped reads (Pce_{S}_a to Pf_{eH}, Pce_{S}_f to Pa_{T}); (d) regions were intraspecific difference of %-covered bases from obtained RNAseq reads (*Pa* and Pce_{S}_a, *Pf* and Pce_{S}_f) > than interspecific difference of %-covered bases from obtained RNAseq reads (*Pf* and Pce_{S}_a, *Pa* and Pce_{S}_f); (e) regions were the proportion of transcripts with intraspecific amino acid identity (Pa_{T} and Pce_{S}_a, Pf_{eH} and Pce_{S}_f) < than the proportion of transcripts with interspecific amino acid identity (Pf_{eH} and Pce_{S}_a , Pa_{T} and Pce_{S}_f).

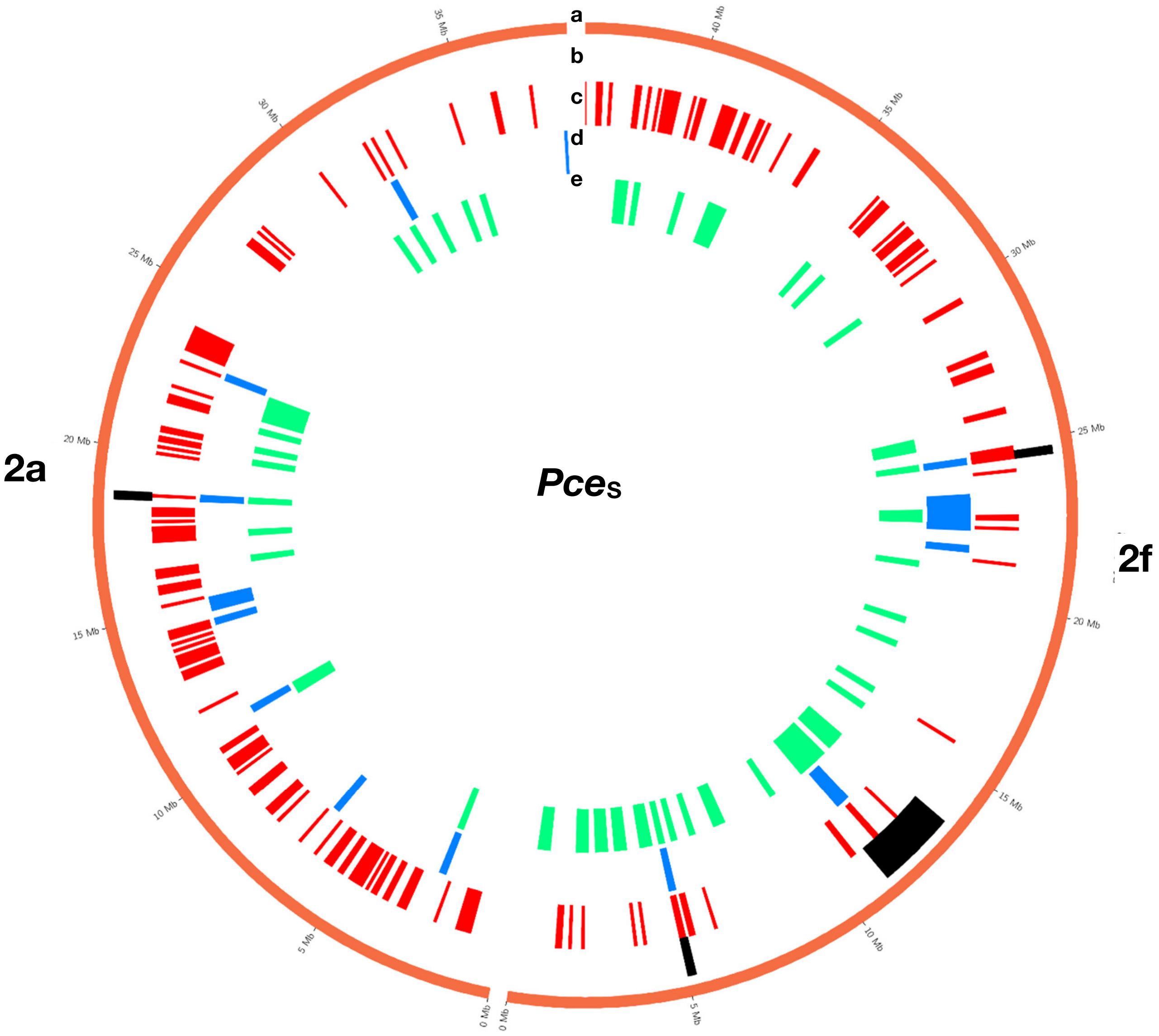

Continuation of figure S10 Detected regions of homoeologous exchanges in the genome of *P. cerasus* 'Schattenmorelle', pseudomolecule 2.

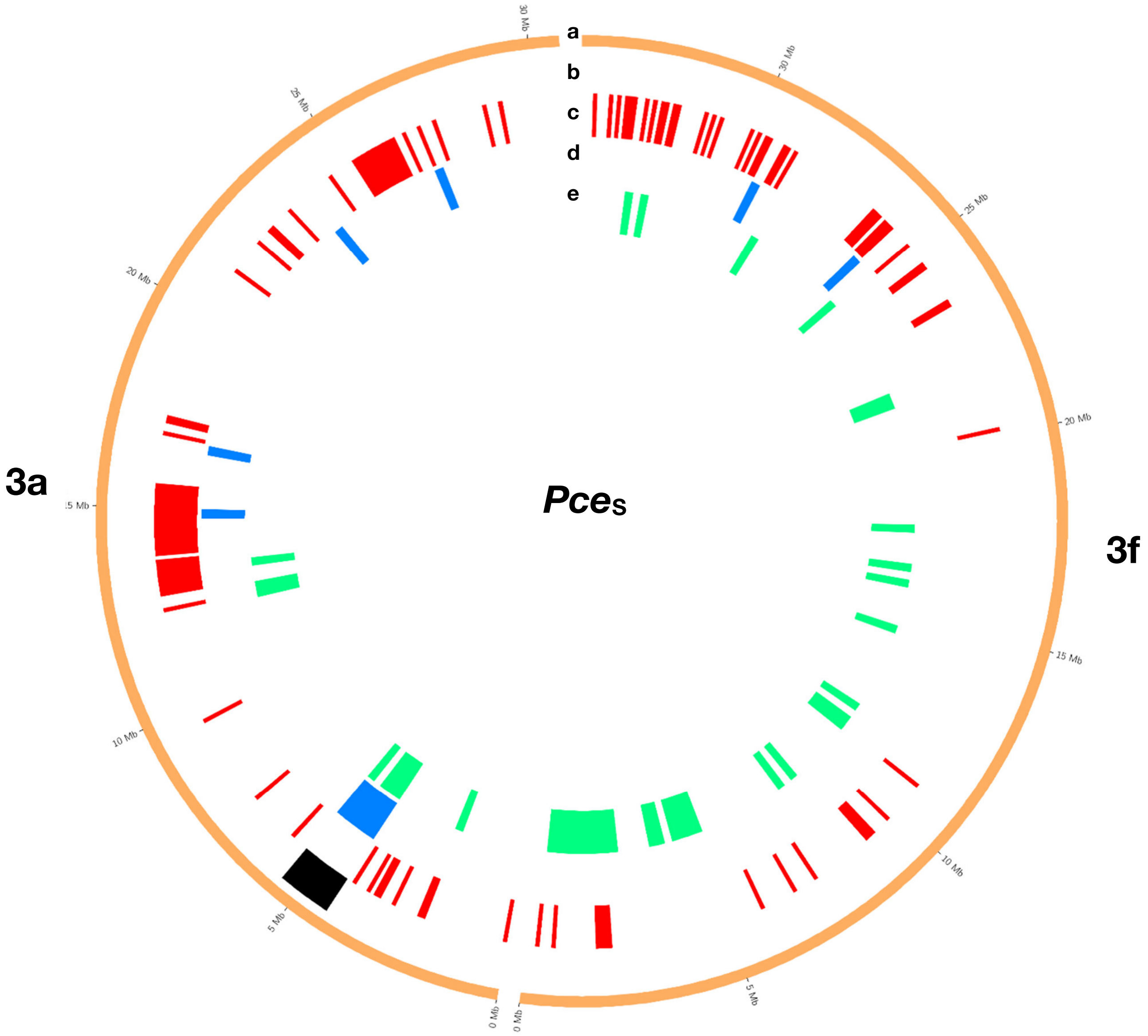

Continuation of figure S10 Detected regions of homoeologous exchanges in the genome of *P. cerasus* 'Schattenmorelle', pseudomolecule 3.

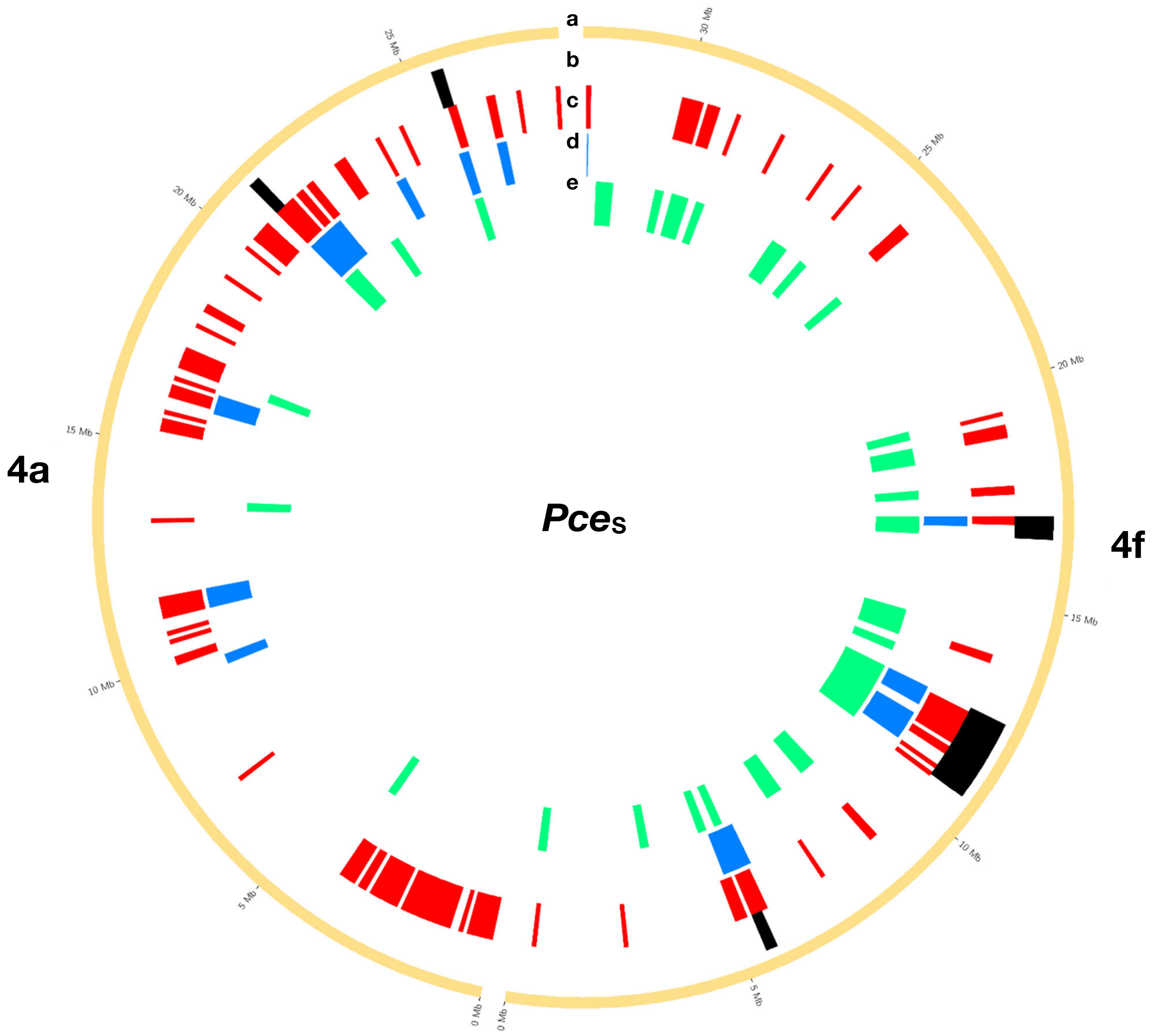

Continuation of figure S10 Detected regions of homoeologous exchanges in the genome of *P. cerasus* 'Schattenmorelle', pseudomolecule 4.

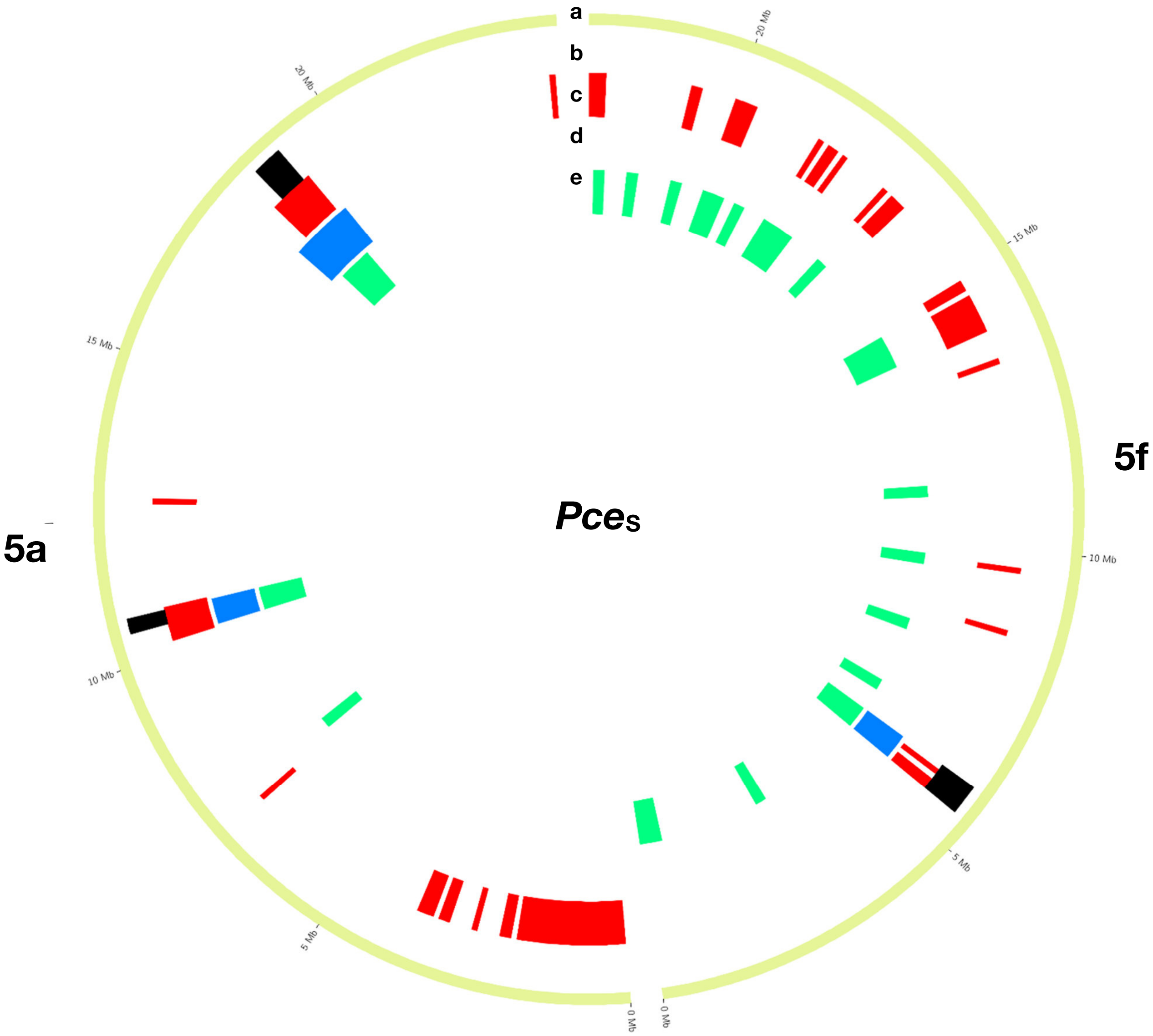

Continuation of figure S10 Detected regions of homoeologous exchanges in the genome of *P. cerasus* 'Schattenmorelle', pseudomolecule 5.

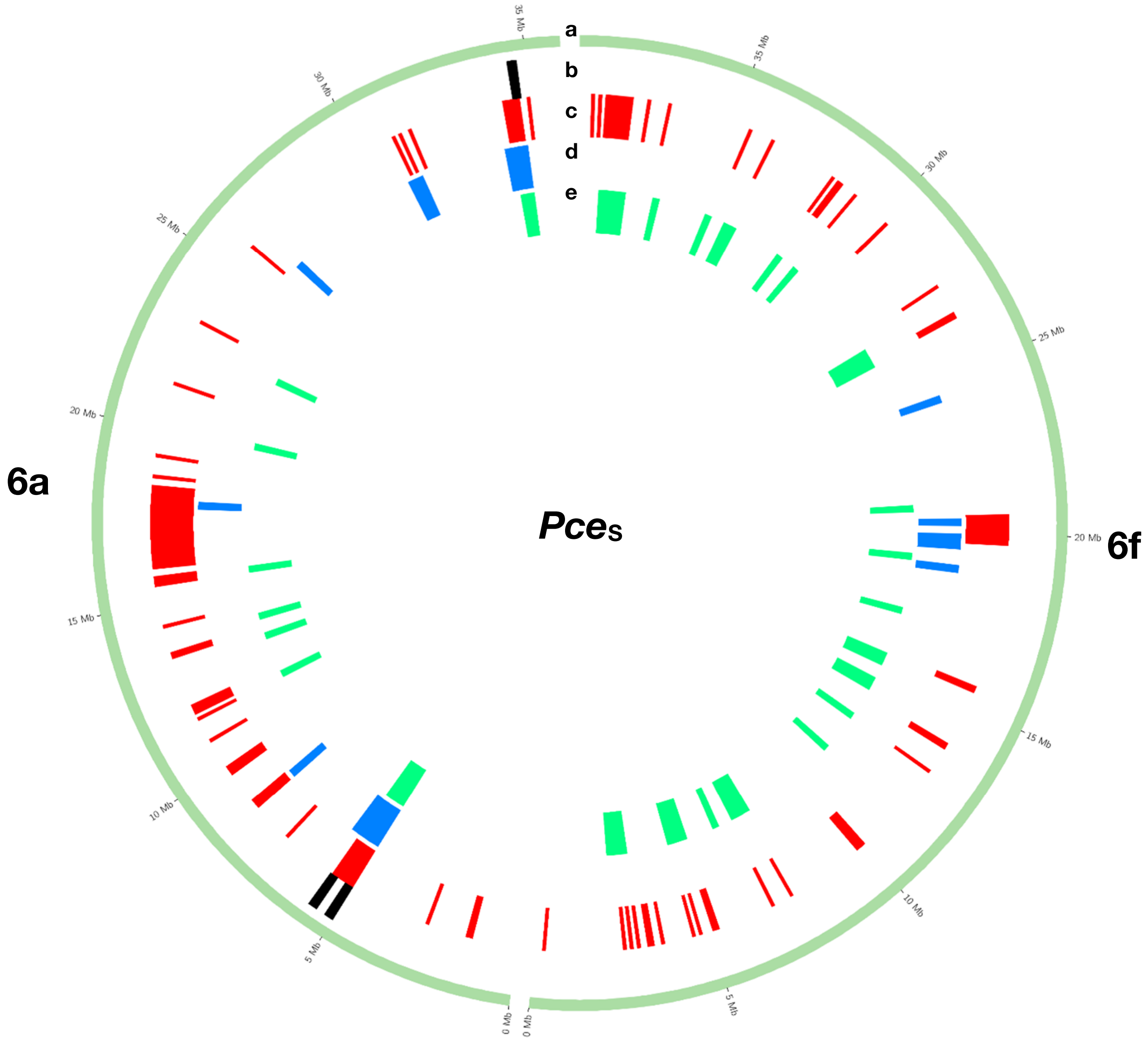

Continuation of figure S10 Detected regions of homoeologous exchanges in the genome of *P. cerasus* 'Schattenmorelle', pseudomolecule 6.

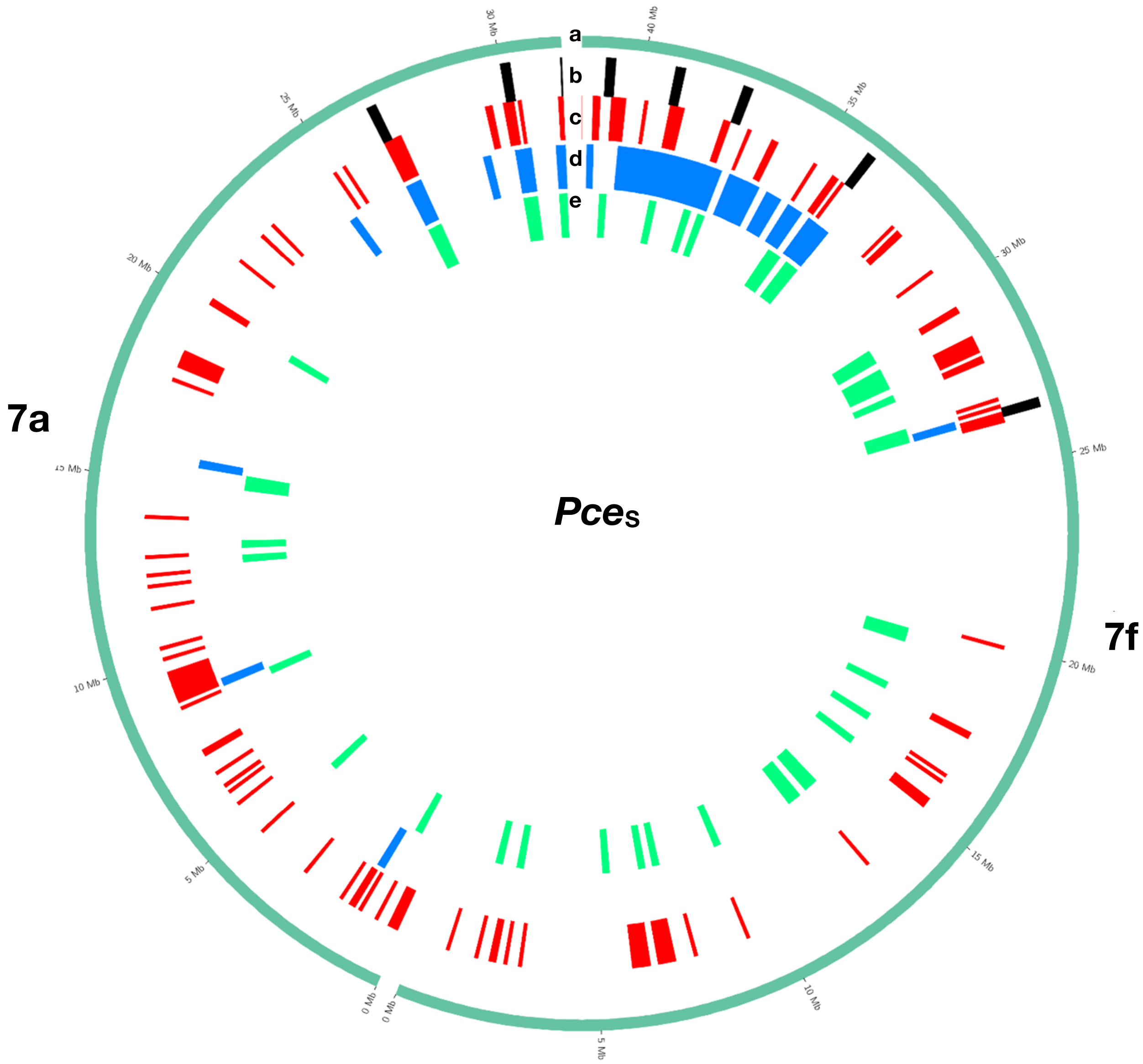

Continuation of figure S10 Detected regions of homoeologous exchanges in the genome of *P. cerasus* 'Schattenmorelle', pseudomolecule 7.

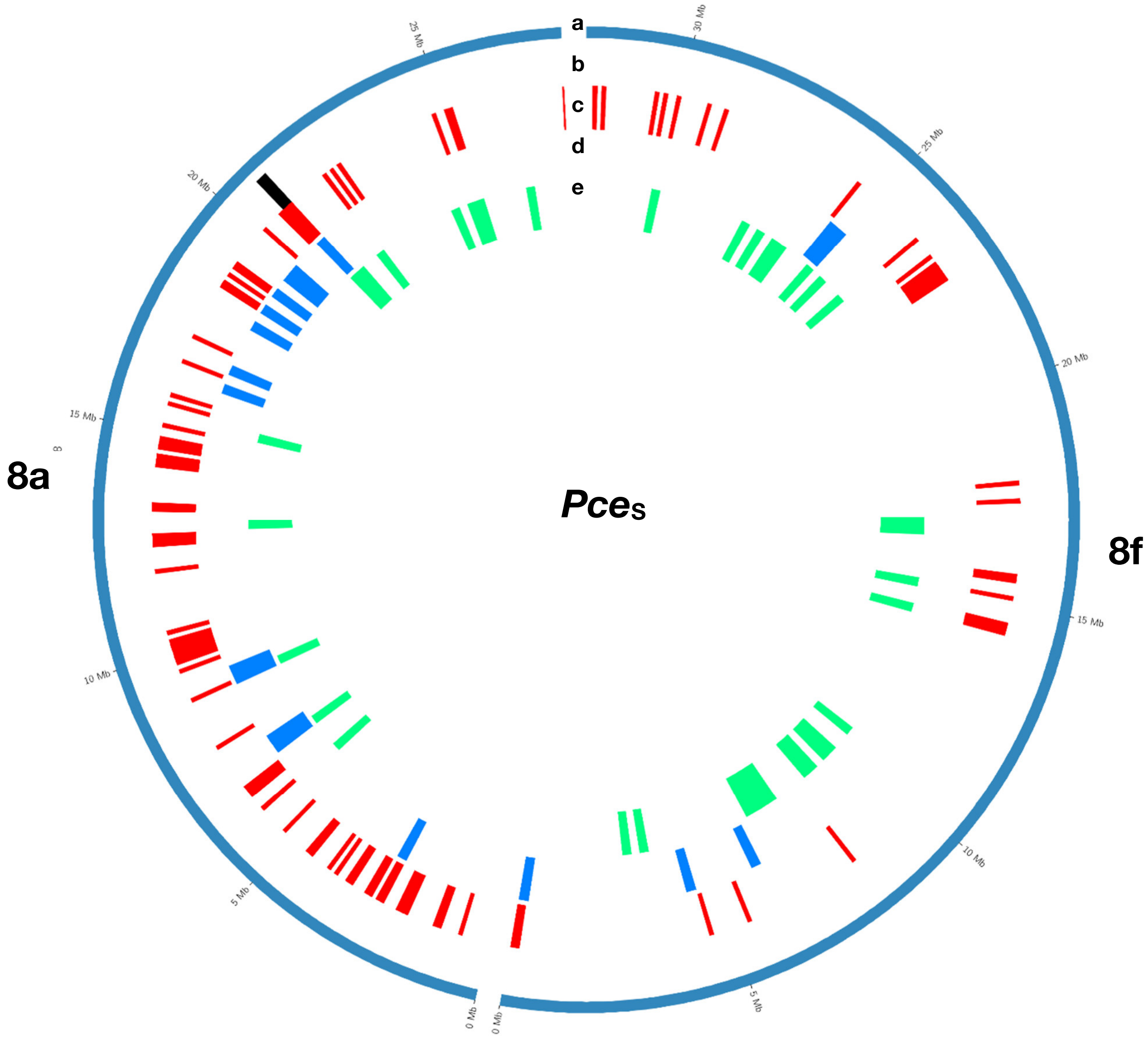

Continuation of figure S10 Detected regions of homoeologous exchanges in the genome of *P. cerasus* 'Schattenmorelle', pseudomolecule 8.

Table S1 Properties of the *Pces*_a and *Pces*_f subgenomes after contig separation.

	*Pces*_a	*Pces*_a purged	*Pces*_f	*Pces*_f purged
Contigs	1714	1058	2036	1065
Total assembly size (Kb)	473,2	339,3	598,7	411,3
N50 size (bp)	328,2	406,2	460,3	592,4
N50 index	434	263	358	200
N90 size (bp)	142,7	164,1	128,8	169,5
N90 index	1,321	777	1,397	698
complete_busco	-	1340	-	1270
% complete	-	93.1	-	88.2
complete_and_single_copy_busco	-	1209	-	982
complete_and_duplicated_busco	-	131	-	288
fragmented_busco	-	25	-	37
missing_busco	-	75	-	9.2
total_busco_searched	-	1440	-	1440

Table S2 Pseudomolecule statistics for *Pce*s

Pseudomolecule	Total size (bp)	%
*Pce*s _a_1.0_chr1	52824182	9.3
*Pce*s _a _1.0_chr2	38212775	6.7
*Pce*s _a _1.0_chr3	30675746	5.4
*Pce*s _a _1.0_chr4	28226313	5.0
*Pce*s _a _1.0_chr5	23885644	4.2
*Pce*s _a _1.0_chr6	35906979	6.3
*Pce*s _a _1.0_chr7	31537404	5.5
*Pce*s _a _1.0_chr8	27735405	4.9
*Pce*s _f_1.0_chr1	53495658	9.4
*Pce*s _f _1.0_chr2	43438225	7.6
*Pce*s _f _1.0_chr3	34378064	6.0
*Pce*s _f _1.0_chr4	32374258	5.7
*Pce*s _f _1.0_chr5	22600031	4.0
*Pce*s _f _1.0_chr6	39437034	6.9
*Pce*s _f _1.0_chr7	41603557	7.3
*Pce*s _f _1.0_chr8	32145936	5.7
	568477211	100

Table S3 Iso-Seq results

	No
SMRT cells	2
Circular Consensus Sequencing reads (Raw)	4,815,872
Circular Consensus Sequencing reads (Filtered)	4,499,940
Circular Consensus Sequencing reads (Full-Length)	4,476,054
HQ isoforms	248,218
LQ isoforms	480

Table S4 Functional annotation results generated by interproscan using BRAKER & GeMoMa combination of ab-initio and homology-based structural gene annotation and statistics

Interproscan annotations	No.	*Pce*s_a	*Pce*s_f	Contigs_A	Contigs_F
Coils	26427	12258	11902	983	1284
Gene3D	135081	63213	60649	4627	6592
Hamap	2773	1323	1217	108	125
PANTHER	217622	100591	98613	7622	10796
Pfam	155028	72373	69489	5449	7717
Phobius	314975	147171	139810	11215	16779
PIRSF	9105	4208	4187	328	382
PRINTS	85141	40737	37507	2991	3906
ProDom	1125	484	512	59	70
ProSitePatterns	32422	15152	14560	1097	1613
ProSiteProfiles	89962	42225	40678	3050	4009
SignalP_EUK	11628	5290	5360	373	605
SMART	77106	36030	35078	2420	3578
SUPERFAMILY	104068	48770	46637	3666	4995
TIGRFAM	19751	9185	8780	756	1030
TMHMM	99627	47068	43744	3488	5327
Sum	**1381841**	646078	530906	48232	156625
Genes					
total	60123	26947	27876	2122	3178
annotated	56047	25152	25960	1988	2947
Transcripts					
total	107508	49698	48576	3799	5435
annotated	103243	47807	46585	3657	5194
annotated GO	71870	33554	32284	2561	3471
annotated pathways	9114	4317	4115	299	383
Mean length (bp)	3580	3864	3808	4031	3565
Mean length of predicted proteins	469	469	460	466	450

Table S5 Mapping of marker sequences to *Prunus cerasus* 'Schattenmorelle' (*Pce*S) genome

Genetic map	No. of marker input	No. of mapped markers
M172x25-F1	1795	1514
US-F1	1787	1506
25x25-F1	1807	1510
Montx25-F1	1795	1517
RE-F1	1856	1509

Table S6 Position of inversions within the subgenomes of *Pce*$_S$ indicated by collinearity of marker positions.

Chr. *Pce*$_S$ vs. *Pce*$_S$	position of inversion in Mbp	Chr. *Pce*$_S$_a vs. *Pa*$_T$	position of inversion in Mbp	Chr. *Pce*$_S$_f vs. *Pf*$_{eH}$	position of inversion in Mbp
Chr1a	9-11, 42-43, 51-52	Chr1a	49-52	Chr1f	50-53
Chr1f	11-12, 43-44, 52-53	Chr1a	56-62	Chr1f	61-65
Chr2a	-	Chr2a	-	Chr2f	-
Chr2f	-	Chr2a	-	Chr2f	-
Chr3a	30-32	Chr3a	-	Chr3f	7-9, 33-34
Chr3f	33-34	Chr3a	-	Chr3f	8-9, 37-39
Chr4a	1-7	Chr4a	-	Chr4f	1-7
Chr4f	1-7	Chr4a	-	Chr4f	1-7
Chr5a	8-9, 15-17, 22-25	Chr5a	-	Chr5f	6-7, 15-17, 19-22
Chr5f	6-7, 15-17, 19-22	Chr5a	-	Chr5f	7-8, 20-21, 28-31
Chr6a	5-6	Chr6a	-	Chr6f	5-7
Chr6f	5-7	Chr6a	-	Chr6f	6-8
Chr7a	1-7, 29-32	Chr7a	1-7, 17-18, 29-32	Chr7f	10-15, 38-42
Chr7f	10-15, 38-42	Chr7a	1-5, 17-19, 31-32	Chr7f	1-7, 32-37
Chr8a	1-9, 26-28	Chr8a	1-9	Chr8f	1-15
Chr8f	1-15, 30-32	Chr8a	1-12	Chr8f	1-13

Note S1 Calculation of the 70% quantile from IAA.

For each reference species, the best iAA per transcript was determined. Midpoints of all transcripts were calculated. Windows of size 0.5Mb centered at these midpoints were used to determine the 70% quantile of the iAA values. Therefor all transcripts with midpoint in this windows were collected. The 70% quantile was computed using the corresponding iAA values and ignoring missing values. The 70% quantile was plotted in a graph against the two subgenomes Pce_{S}_a and Pce_{S}_f of *P. cerasus* cv 'Schattenmorelle'. A higher IAA value of proteins from *P. avium* cv 'Tieton' (Pa_{T}) in comparison to proteins from *P. fruticosa* ecotype Hármashatárhegy (Pf_{eH}) is expected for Pce_{S}_a and vice versa. An example is given below (Example plot 1). The blue line represents the 70% quantile value obtained from the comparison between Pce_{S}_a and Pa_{T}. Due to the relation of both species, the blue line is nearly 1, except for some regions. The red line represents the comparison between Pce_{S}_f and $Pf_{\mathrm{eH.}}$ The species are not related which is indicated by a lower 70% quantile value. In case of a translocation between Pce_{S}_a and Pce_{S}_f, the values of the red line will increase whereas the value of the blue line will decrease. An example is given in Example plot 2. Note S2 contains 8 plots were the 70% quantile values of IAA obtained from comparisons between *P. avium* 'Tieton' (Pa_{T}) and *P. fruticosa* ecotype Hármashatárhegy (Pf_{eH}) with *P. cerasus* 'Schattenmorelle' (Pce_{S}) were plotted against the chromosomes of subgenome Pce_{S}_a. Additionally, eight plots were the 70% quantile values of IAA obtained from comparisons between Pa_{T} and Pf_{eH} with Pce_{S} were plotted against the chromosomes of subgenome Pce_{S}_f. The same was calculated for the 14 reference species used in the final annotation procedure.

Comparison of 70%-quantile of IAA between transcripts from *P. cerasus* cv 'Schattenmorelle' assigned to *P. fruticosa* ecotype Hármashatárhegy (Prufru, red) or *P. avium* cv 'Tieton' (Pruavi, blue) for each chromosome.

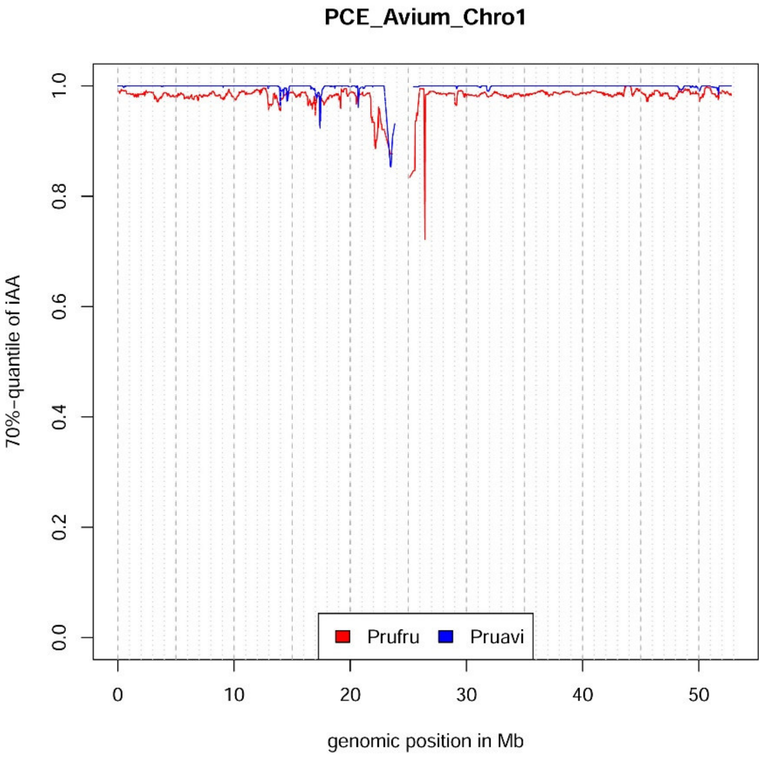

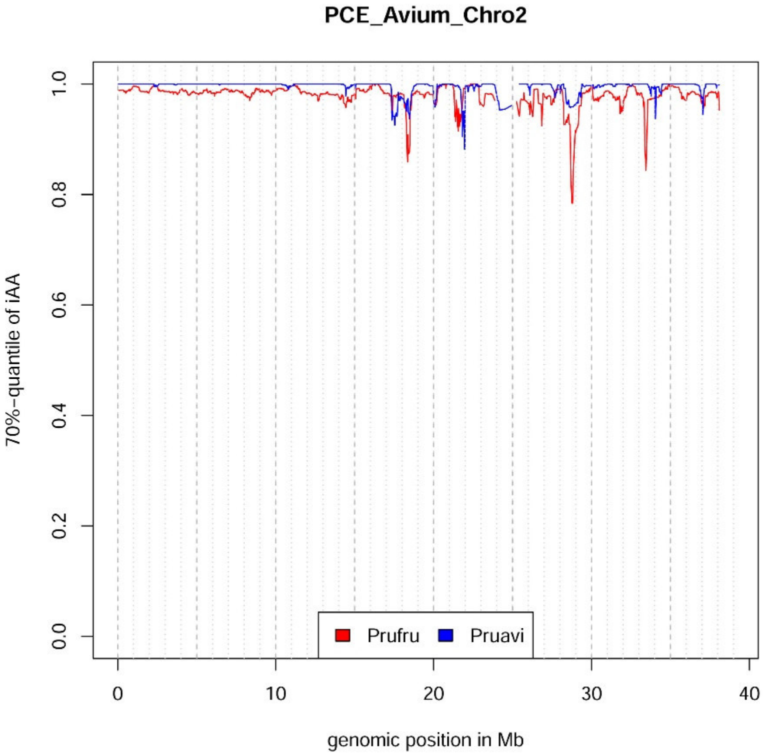

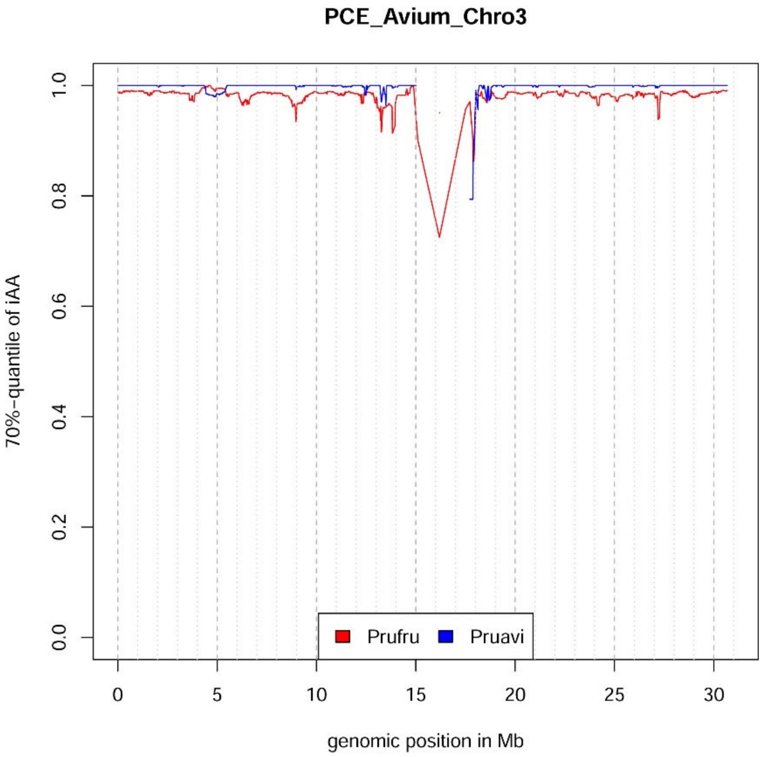
PCE_Avium_Chro3
70%-quantile of iAA
0.0
0.2
0.4
0.6
0.8
1.0
Prufru
Pruavi
0
5
10
15
20
25
30
genomic position in Mb

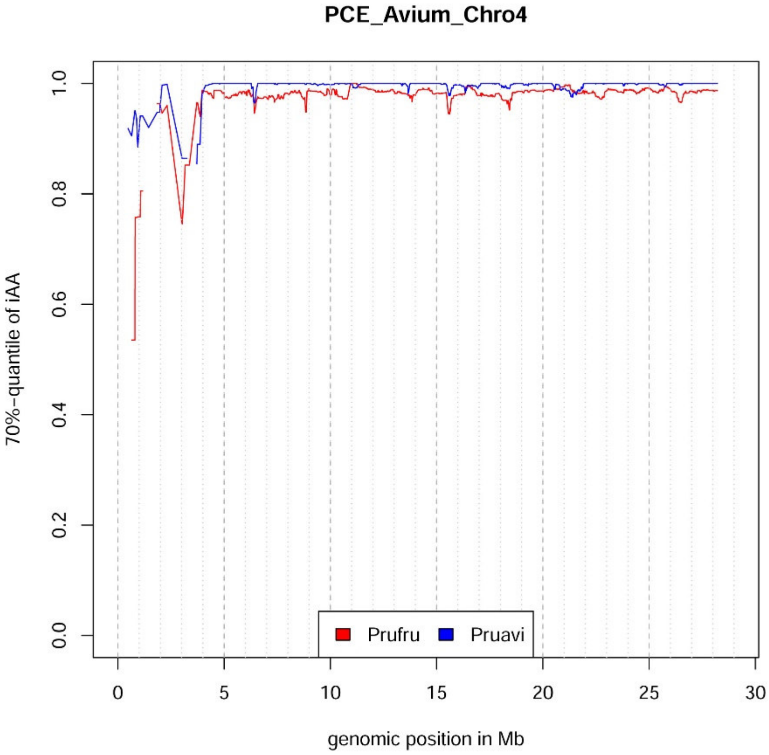
PCE_Avium_Chro4
70%-quantile of iAA
0.0
0.2
0.4
0.6
0.8
1.0
Prufru
Pruavi
0
5
10
15
20
25
30
genomic position in Mb

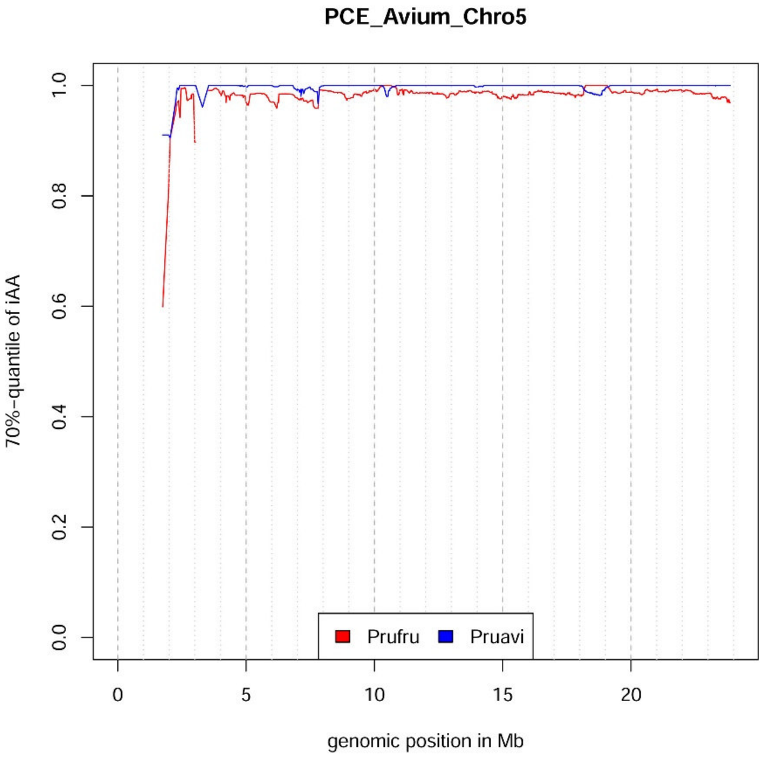
PCE_Avium_Chro5
70%-quantile of iAA
1.0
0.8
0.6
0.4
0.2
0.0
Prufru
Pruavi
0
5
10
15
20
genomic position in Mb

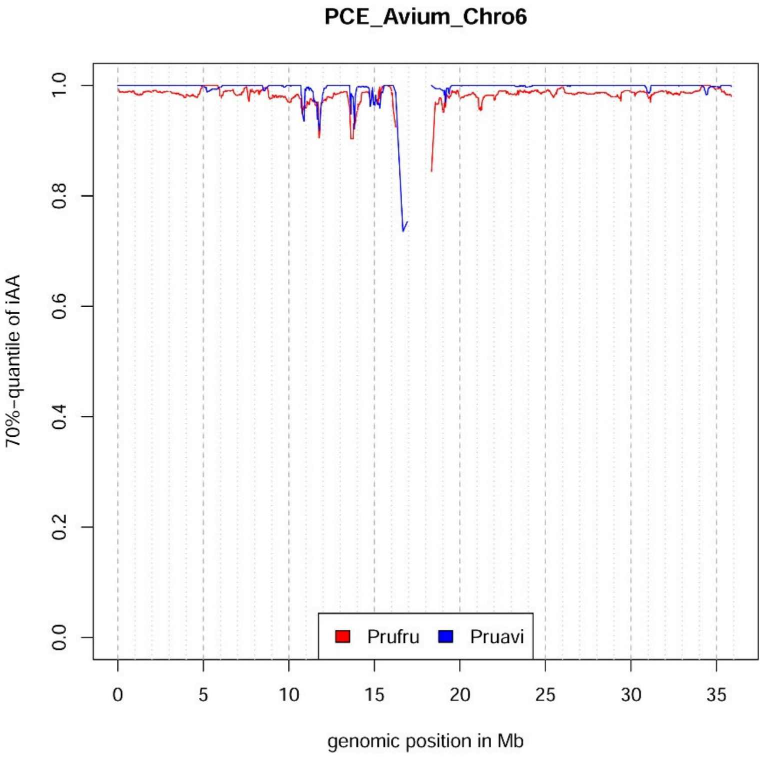
PCE_Avium_Chro6
70%-quantile of iAA
1.0
0.8
0.6
0.4
0.2
0.0
Prufru
Pruavi
0
5
10
15
20
25
30
35
genomic position in Mb

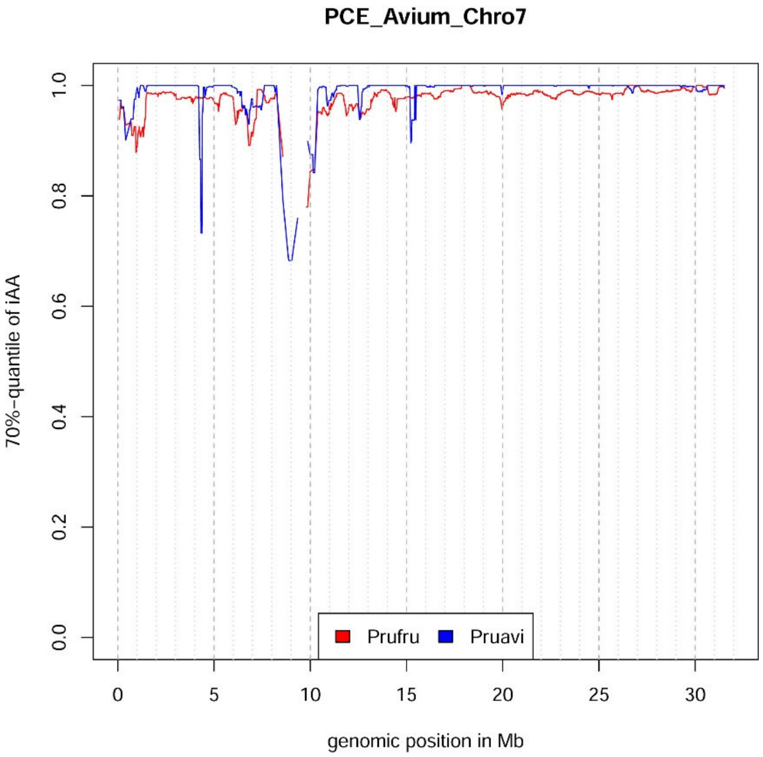
PCE_Avium_Chro7
70%-quantile of iAA
1.0
0.8
0.6
0.4
0.2
0.0
Prufru
Pruavi
0
5
10
15
20
25
30
genomic position in Mb

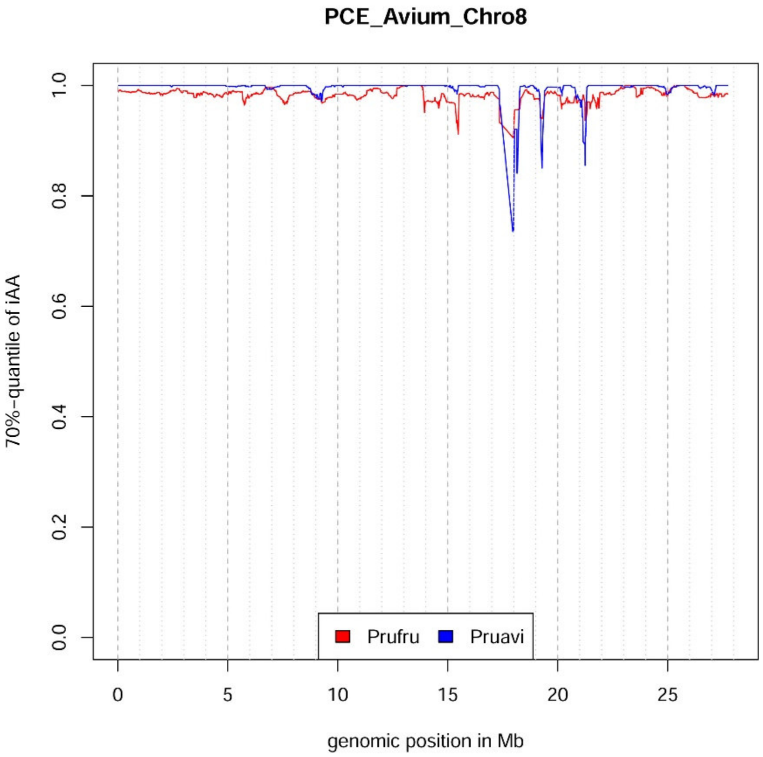
PCE_Avium_Chro8
70%-quantile of iAA
1.0
0.8
0.6
0.4
0.2
0.0
Prufru
Pruavi
0
5
10
15
20
25
genomic position in Mb

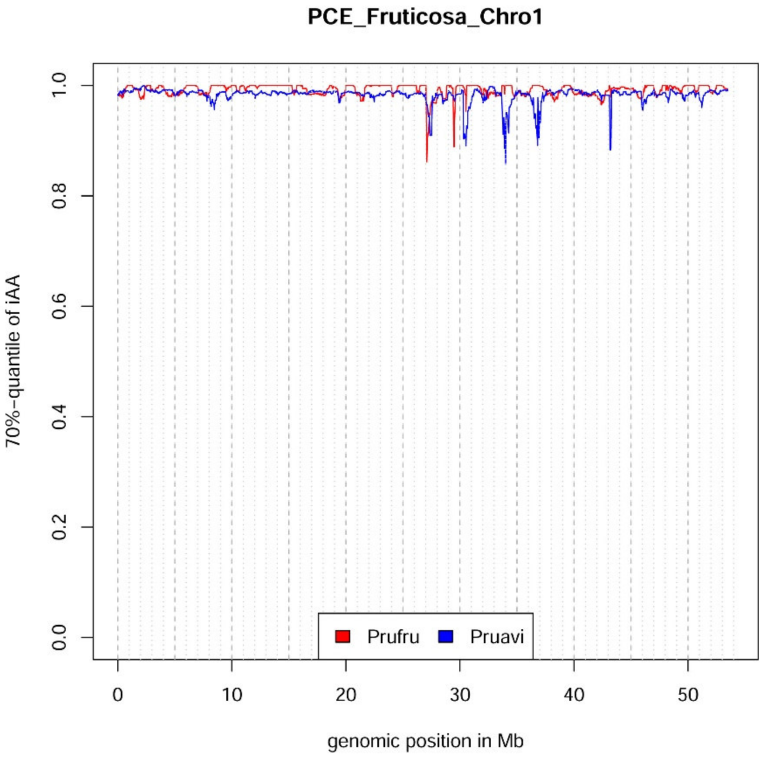
PCE_Fruticosa_Chro1
70%-quantile of iAA
0.0
0.2
0.4
0.6
0.8
1.0
Prufru
Pruavi
0
10
20
30
40
50
genomic position in Mb

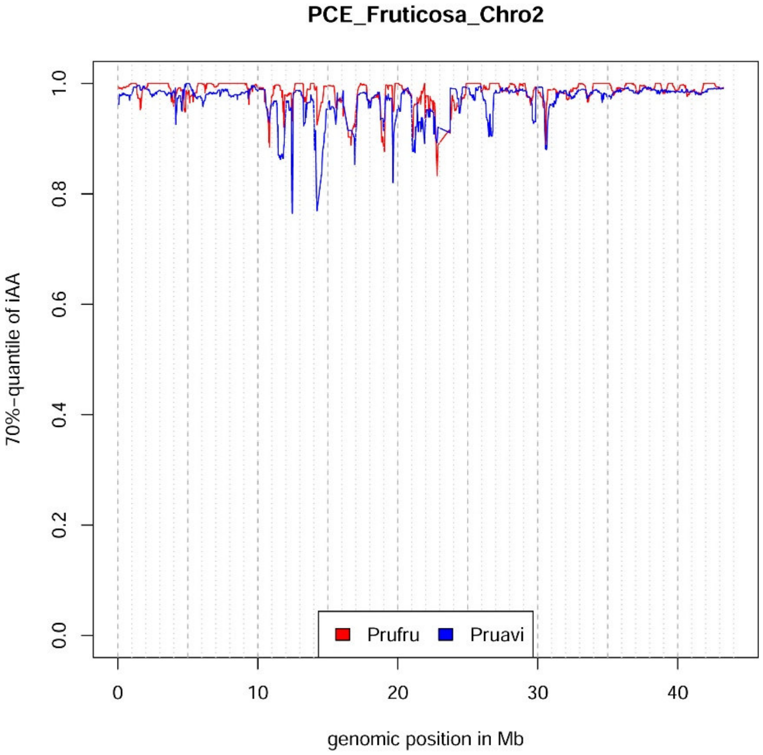
PCE_Fruticosa_Chro2
70%-quantile of iAA
0.0
0.2
0.4
0.6
0.8
1.0
Prufru
Pruavi
0
10
20
30
40
genomic position in Mb

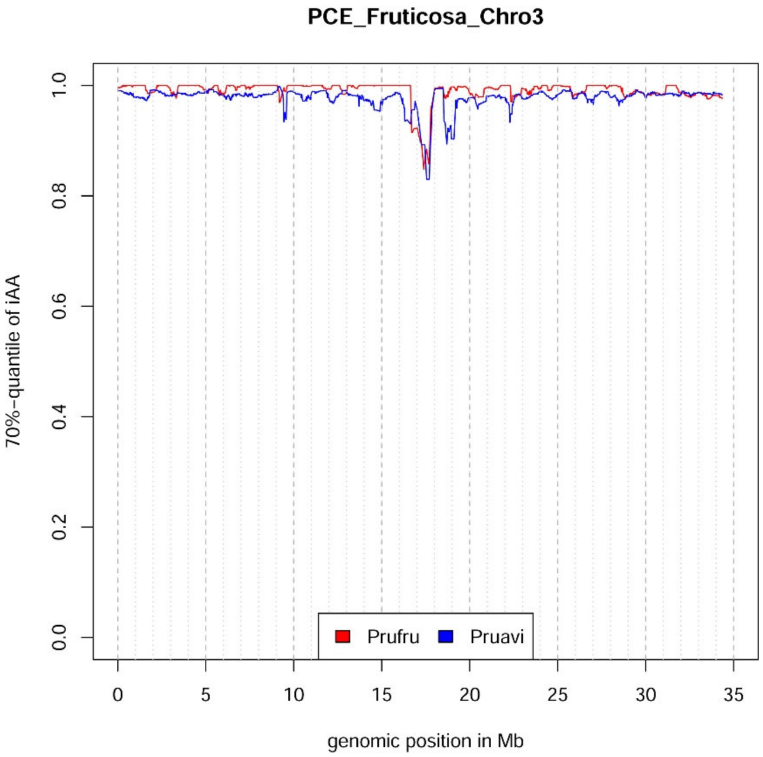
PCE_Fruticosa_Chro3
70%-quantile of iAA
1.0
0.8
0.6
0.4
0.2
0.0
Prufru
Pruavi
0
5
10
15
20
25
30
35
genomic position in Mb

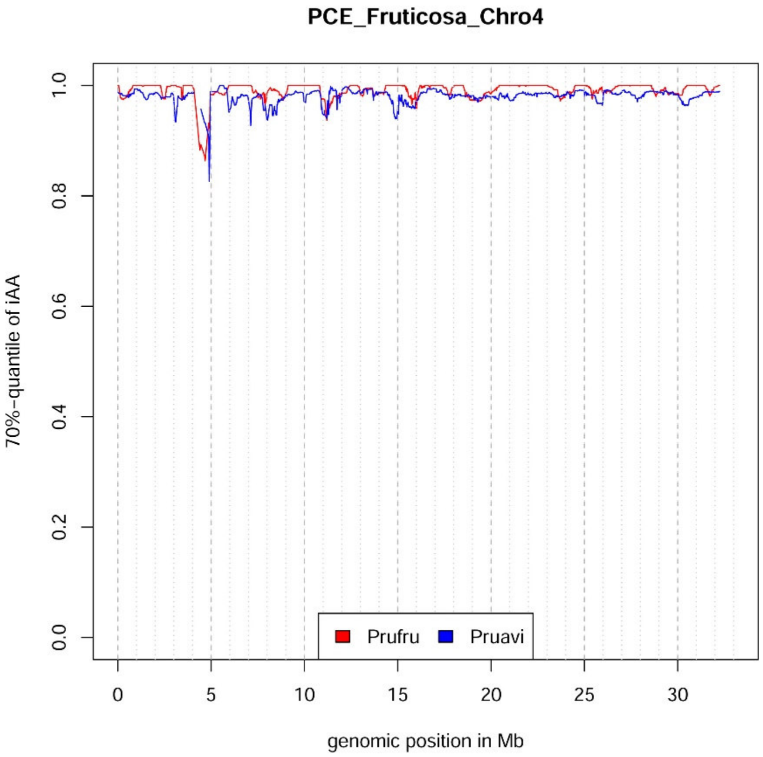
PCE_Fruticosa_Chro4
70%-quantile of iAA
1.0
0.8
0.6
0.4
0.2
0.0
Prufru
Pruavi
0
5
10
15
20
25
30
genomic position in Mb

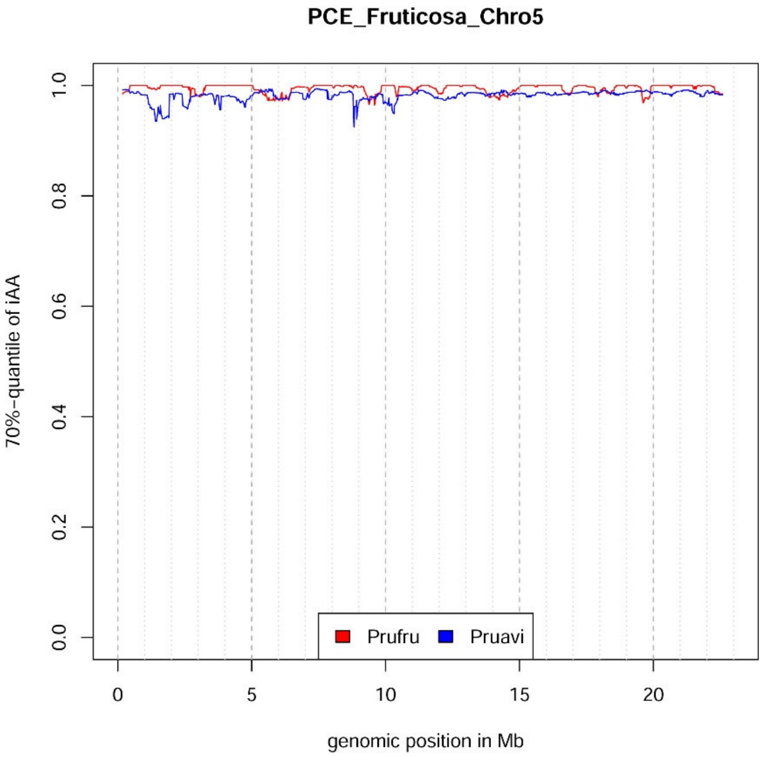
PCE_Fruticosa_Chro5
70%-quantile of iAA
1.0
0.8
0.6
0.4
0.2
0.0
Prufru
Pruavi
0
5
10
15
20
genomic position in Mb

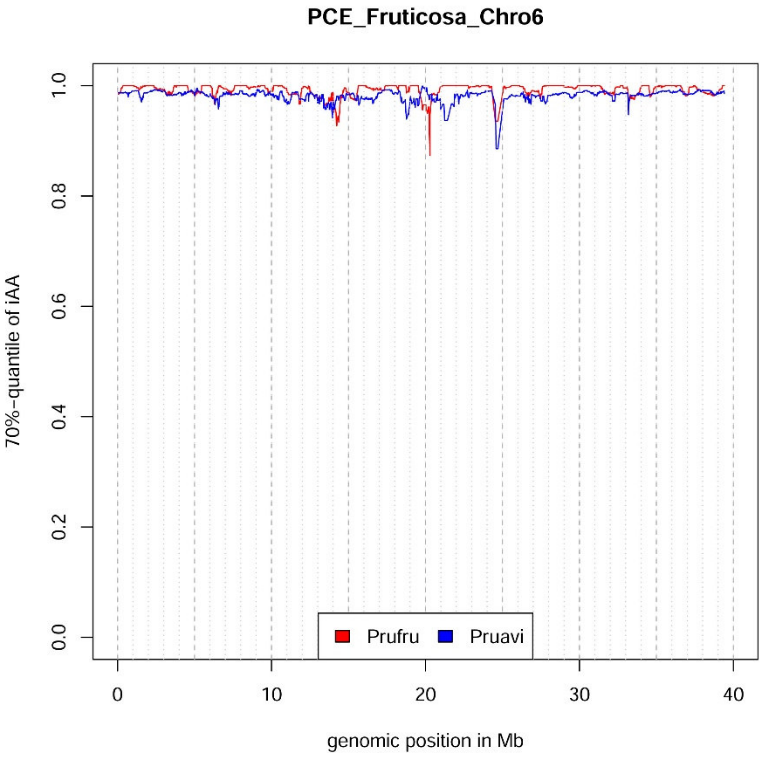
PCE_Fruticosa_Chro6
70%-quantile of iAA
1.0
0.8
0.6
0.4
0.2
0.0
Prufru
Pruavi
0
10
20
30
40
genomic position in Mb

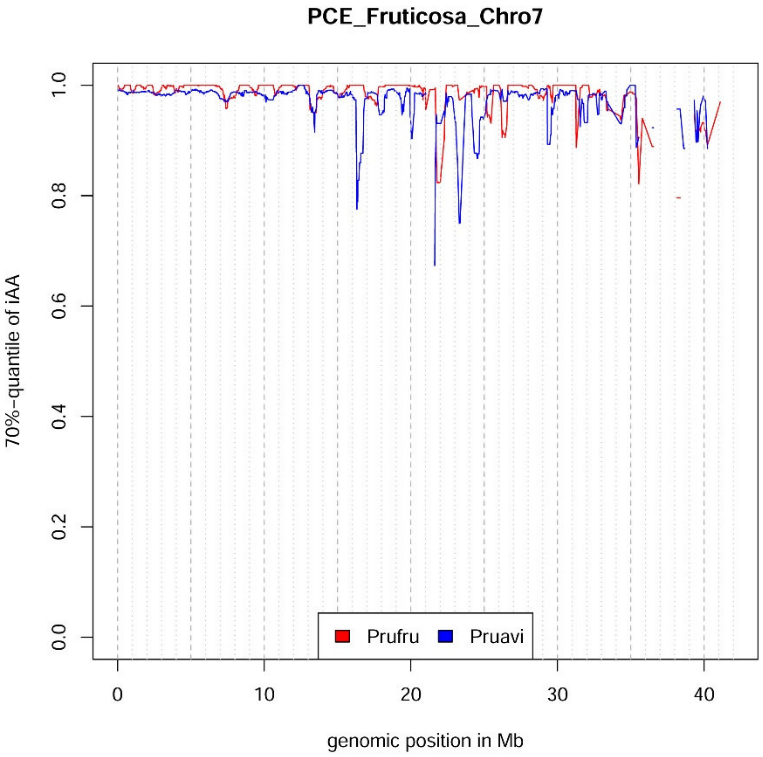
PCE_Fruticosa_Chro7
70%-quantile of iAA
0.0
0.2
0.4
0.6
0.8
1.0
0
10
20
30
40
genomic position in Mb
Prufru
Pruavi

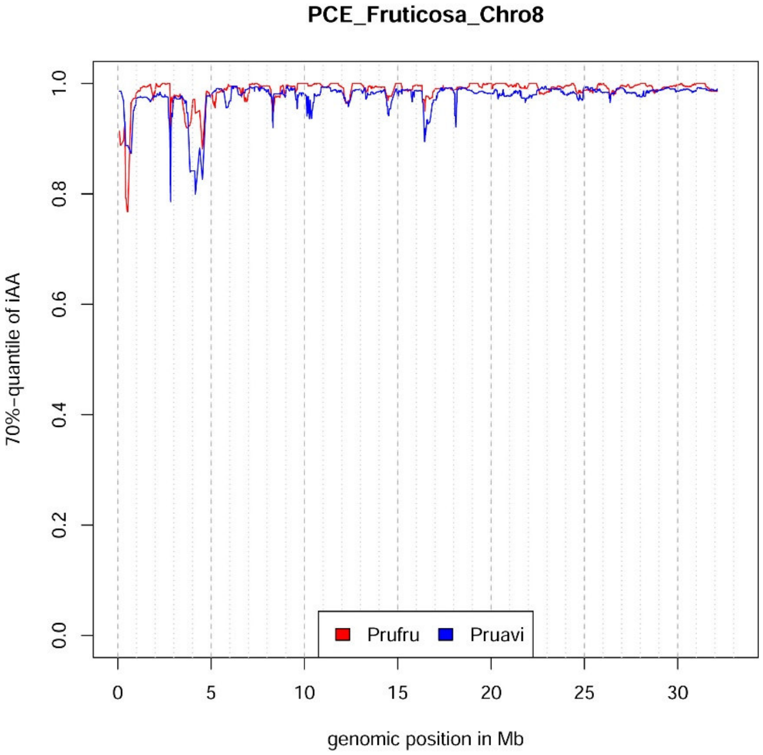
PCE_Fruticosa_Chro8
70%-quantile of iAA
0.0
0.2
0.4
0.6
0.8
1.0
0
5
10
15
20
25
30
genomic position in Mb
Prufru
Pruavi

Comparison of 70%-quantile of IAA between transcripts from *P. cerasus* cv 'Schattenmorelle' assigned to *P. fruticosa* ecotype Hármashatárhegy (Prufru, red), *P. avium* cv 'Tieton' (Pruavi, blue), *P. yedonensis* (Pyed, green), *P. domestica* (Pd, brown), *P. armeniaca* (Par, orange), *P. persica* (Pp, black), *Pyrus communis* (Pyrco, rose pink), *Populus trichocarpa* (Poptri, *yellow*), *Vitis vinifera* (Vv, green-blue), *Arabidopsis thaliana* (At, pink), *Malus domestica* (Md, lime pink), *Fragaria vesca* (Fraves, hot pink), *Rubus occidentalis* (Rubocc, mint-green), for each chromosome.

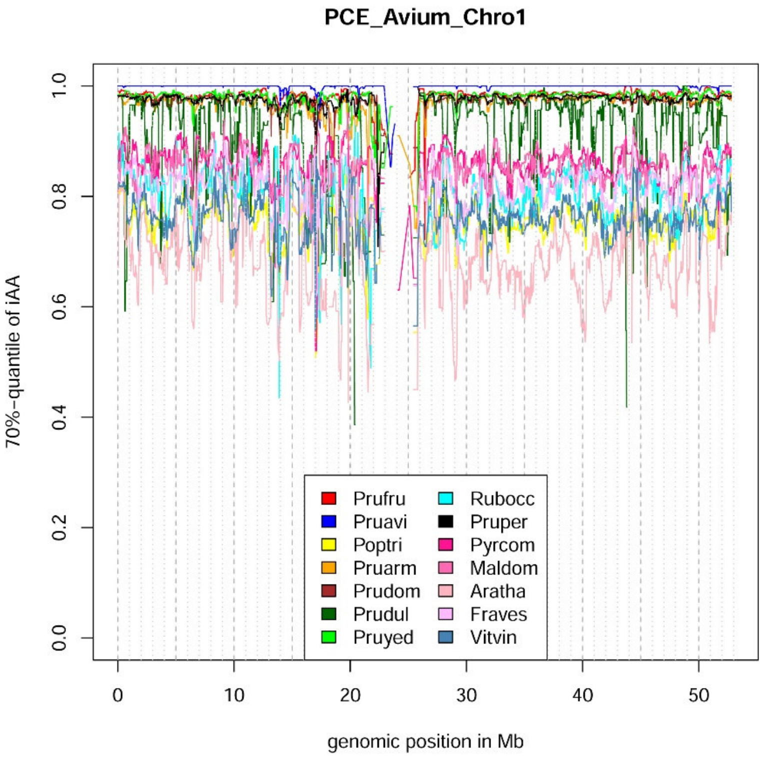

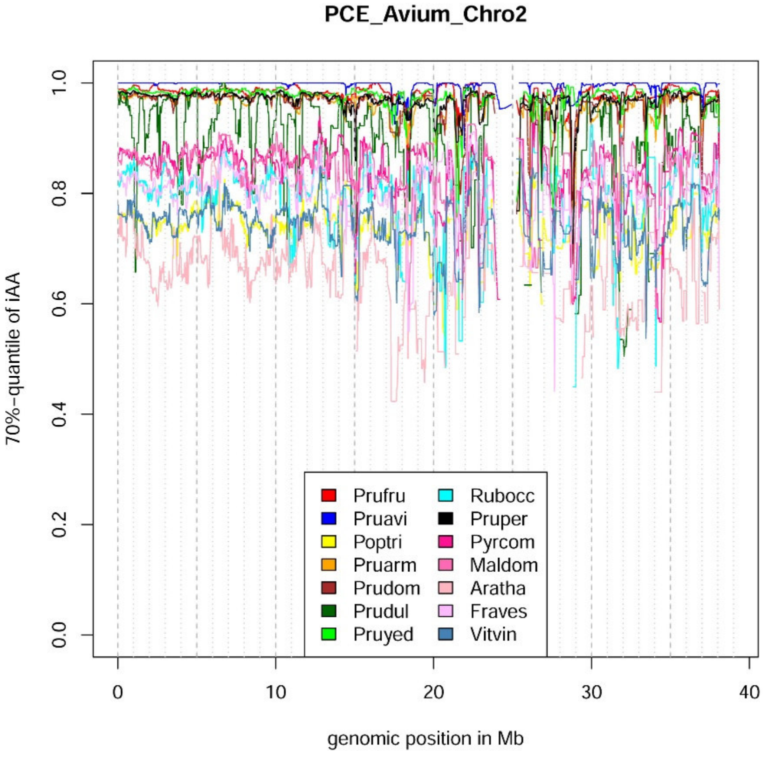

PCE_Avium_Chro3

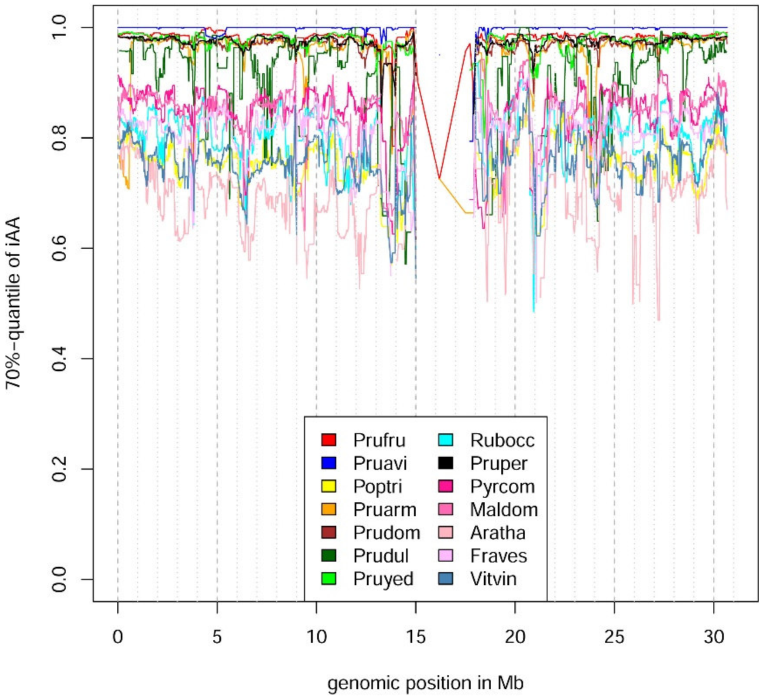

PCE_Avium_Chro4

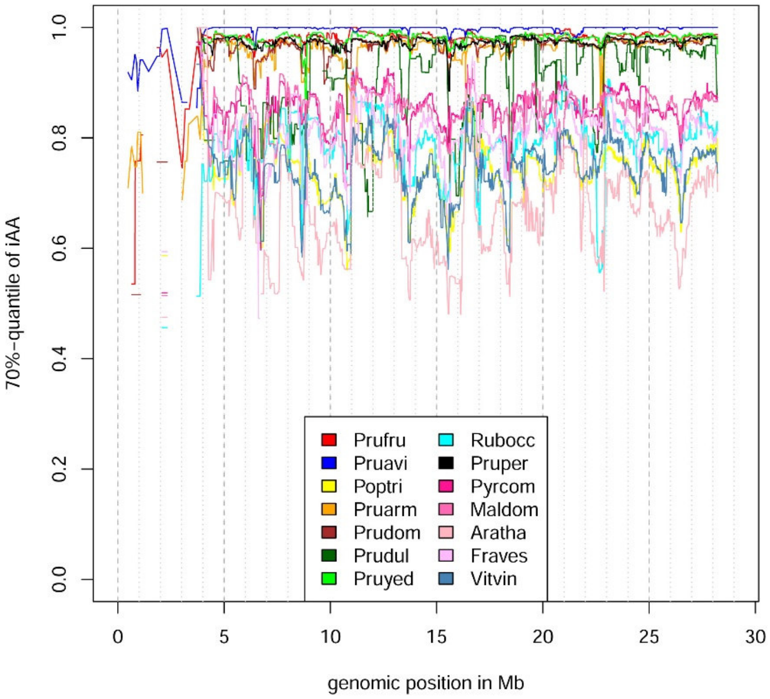

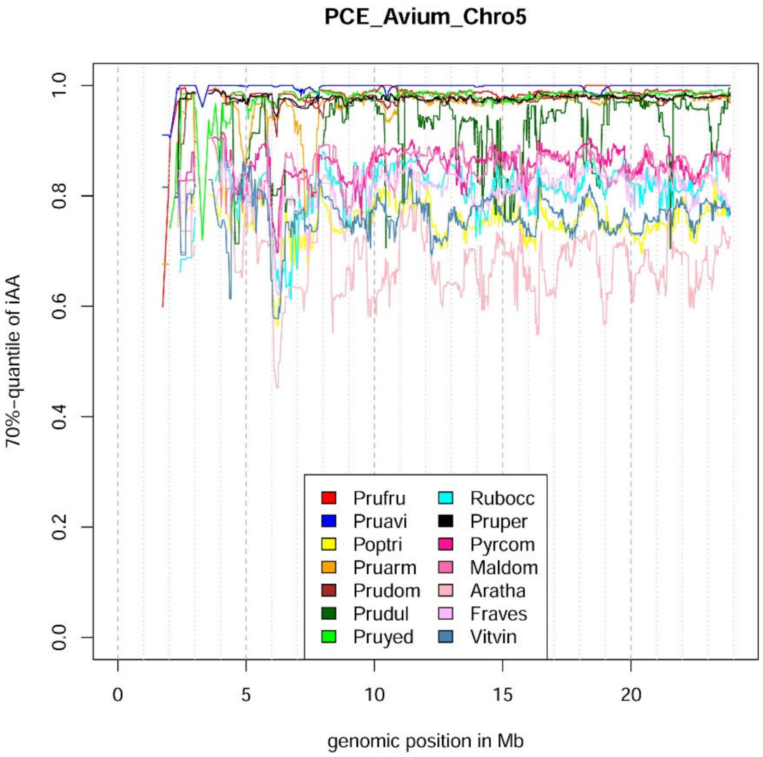
PCE_Avium_Chro5
70%-quantile of iAA
genomic position in Mb
Prufru
Pruavi
Poptri
Pruarm
Prudom
Prudul
Pruyed
Rubocc
Pruper
Pyrcom
Maldom
Aratha
Fraves
Vitvin

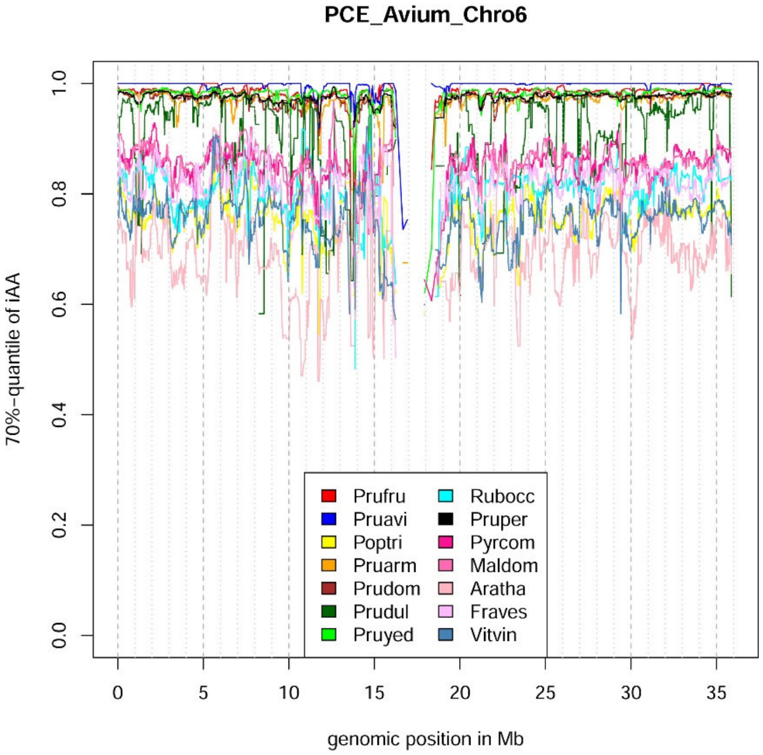
PCE_Avium_Chro6
70%-quantile of iAA
genomic position in Mb
Prufru
Pruavi
Poptri
Pruarm
Prudom
Prudul
Pruyed
Rubocc
Pruper
Pyrcom
Maldom
Aratha
Fraves
Vitvin

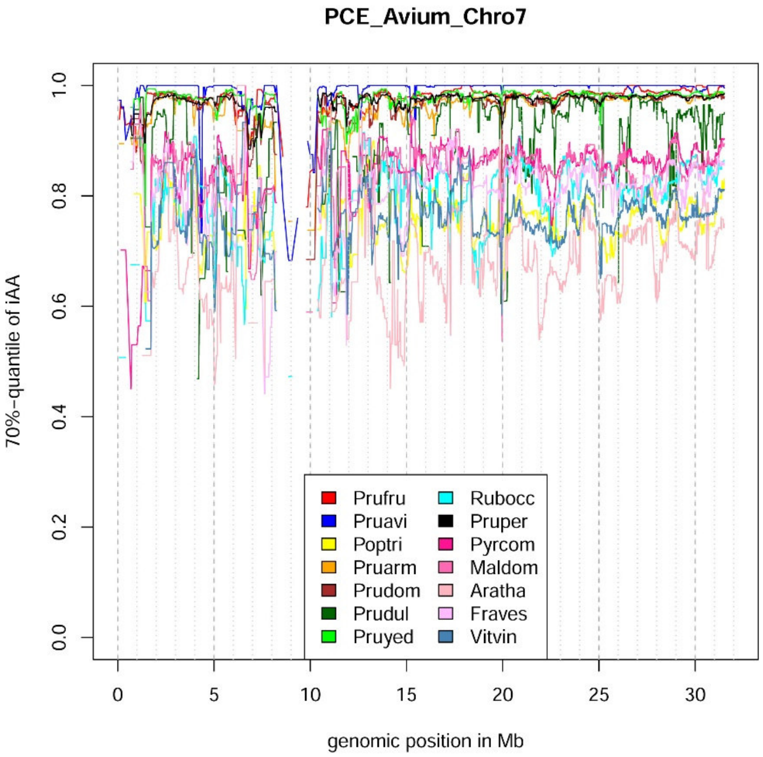

PCE_Avium_Chro7
70%-quantile of iAA
genomic position in Mb
Prufru
Pruavi
Poptri
Pruarm
Prudom
Prudul
Pruyed
Rubocc
Pruper
Pyrcom
Maldom
Aratha
Fraves
Vitvin

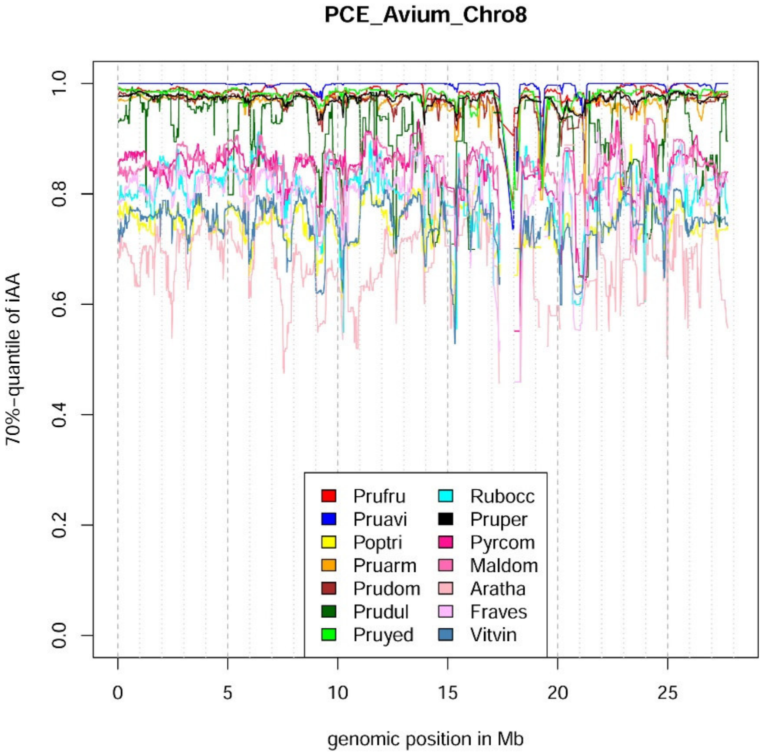

PCE_Avium_Chro8
70%-quantile of iAA
genomic position in Mb
Prufru
Pruavi
Poptri
Pruarm
Prudom
Prudul
Pruyed
Rubocc
Pruper
Pyrcom
Maldom
Aratha
Fraves
Vitvin

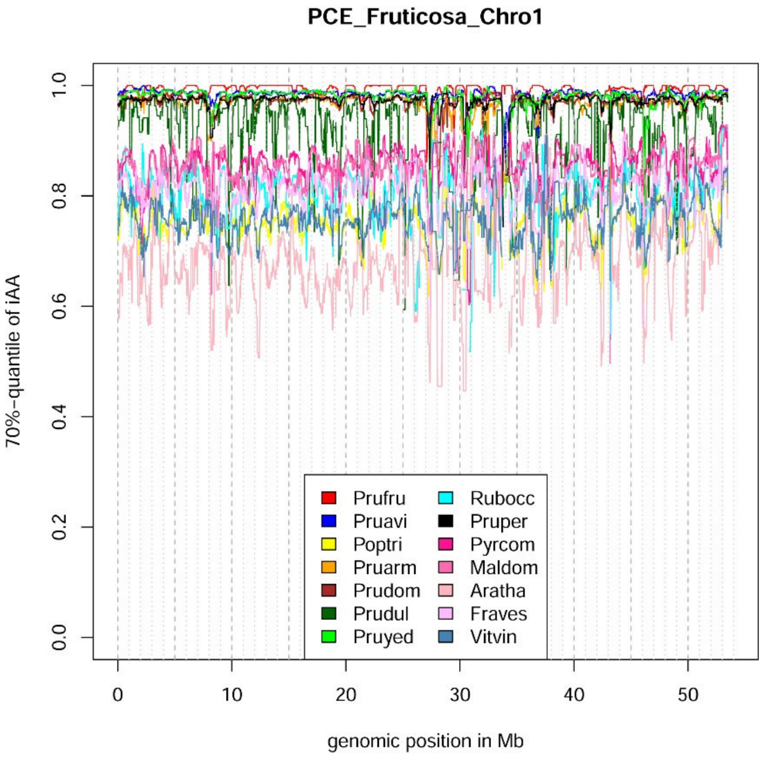
PCE_Fruticosa_Chro1
70%-quantile of iAA
genomic position in Mb
Prufru
Pruavi
Poptri
Pruarm
Prudom
Prudul
Pruyed
Rubocc
Pruper
Pyrcom
Maldom
Aratha
Fraves
Vitvin

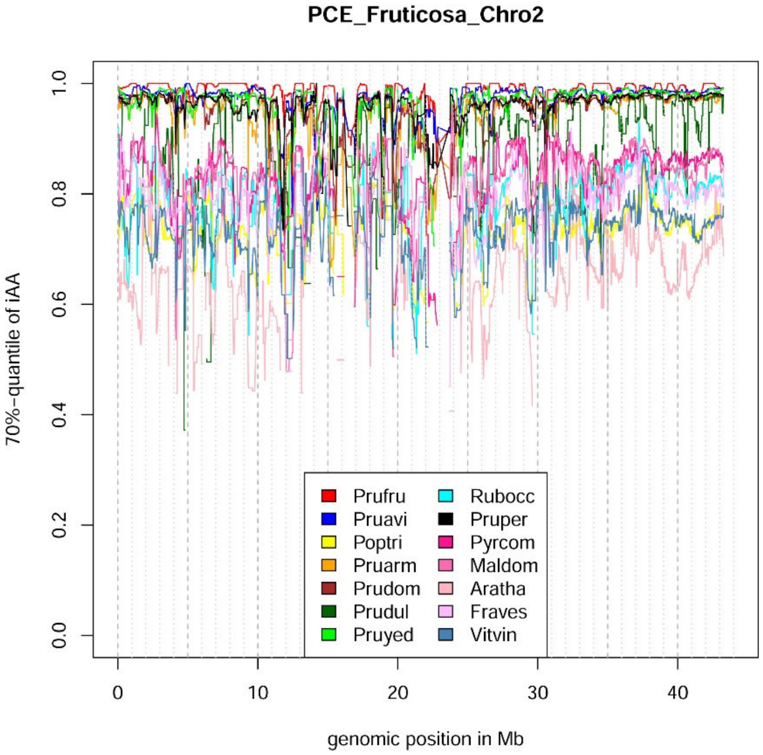
PCE_Fruticosa_Chro2
70%-quantile of iAA
genomic position in Mb
Prufru
Pruavi
Poptri
Pruarm
Prudom
Prudul
Pruyed
Rubocc
Pruper
Pyrcom
Maldom
Aratha
Fraves
Vitvin

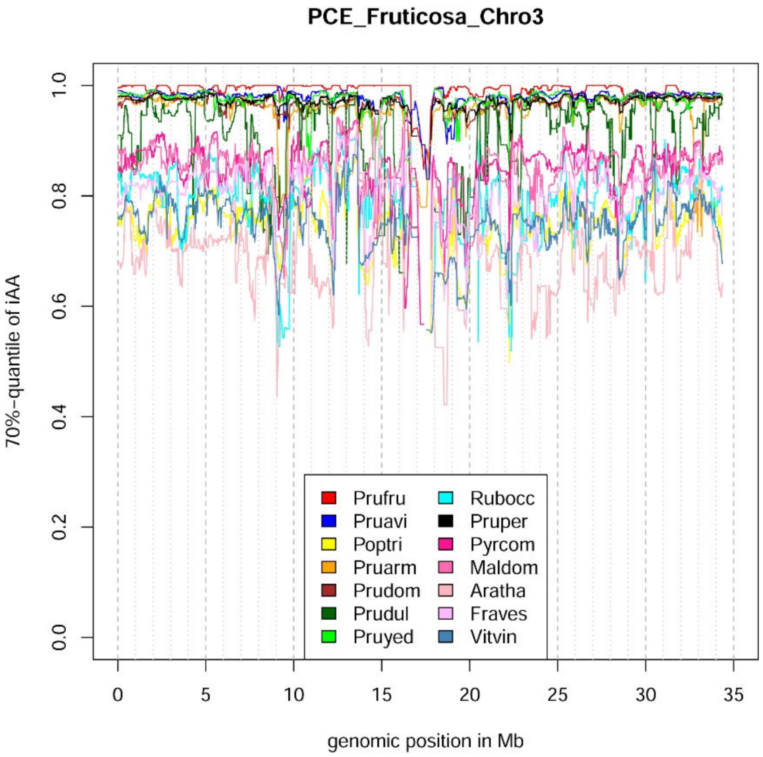
PCE_Fruticosa_Chro3
70%-quantile of iAA
genomic position in Mb
0.0
0.2
0.4
0.6
0.8
1.0
0
5
10
15
20
25
30
35
Prufru
Pruavi
Poptri
Pruarm
Prudom
Prudul
Pruyed
Rubocc
Pruper
Pyrcom
Maldom
Aratha
Fraves
Vitvin

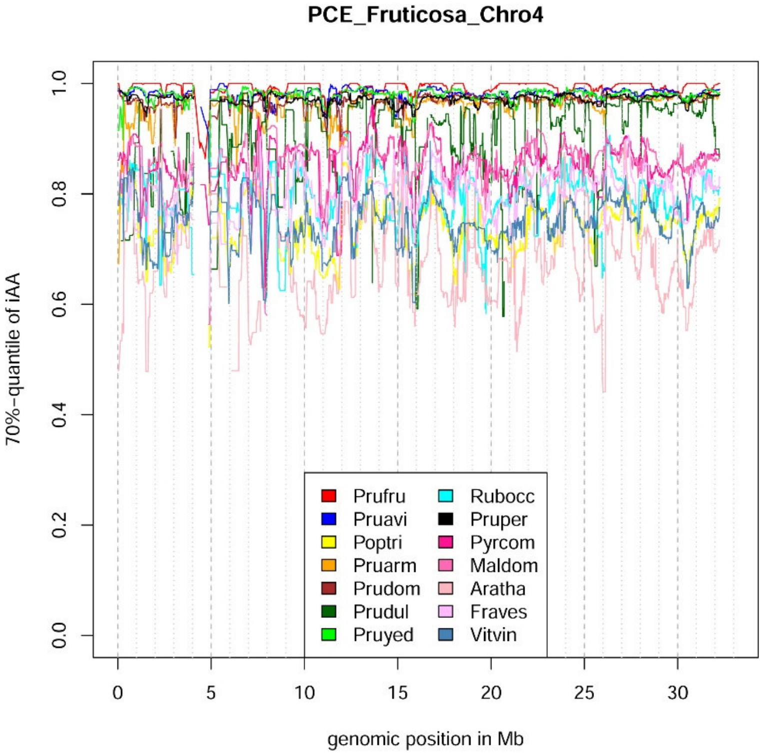
PCE_Fruticosa_Chro4
70%-quantile of iAA
genomic position in Mb
0.0
0.2
0.4
0.6
0.8
1.0
0
5
10
15
20
25
30
Prufru
Pruavi
Poptri
Pruarm
Prudom
Prudul
Pruyed
Rubocc
Pruper
Pyrcom
Maldom
Aratha
Fraves
Vitvin

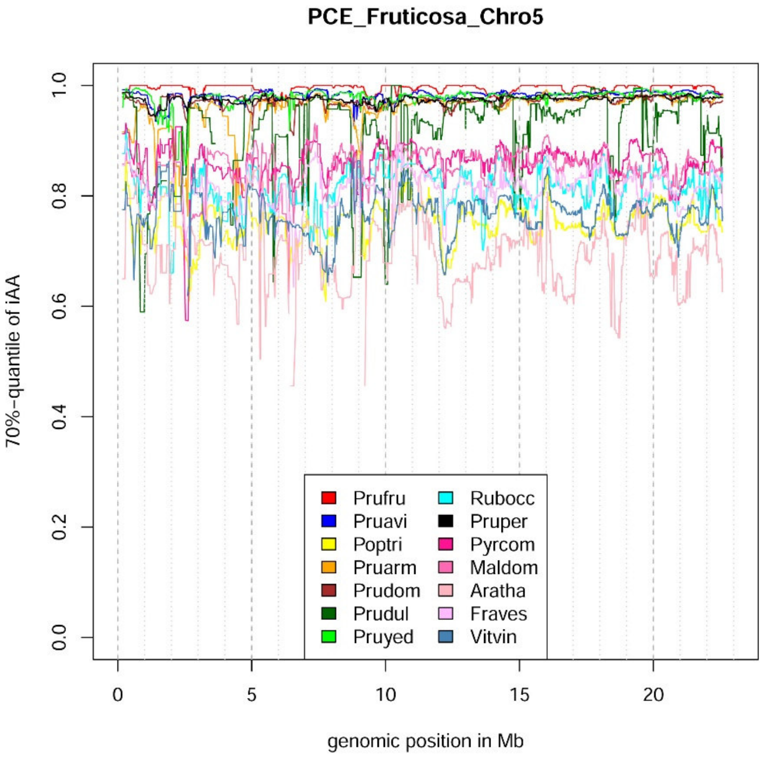
PCE_Fruticosa_Chro5
70%-quantile of iAA
0.0
0.2
0.4
0.6
0.8
1.0
0
5
10
15
20
genomic position in Mb
Prufru
Pruavi
Poptri
Pruarm
Prudom
Prudul
Pruyed
Rubocc
Pruper
Pyrcom
Maldom
Aratha
Fraves
Vitvin

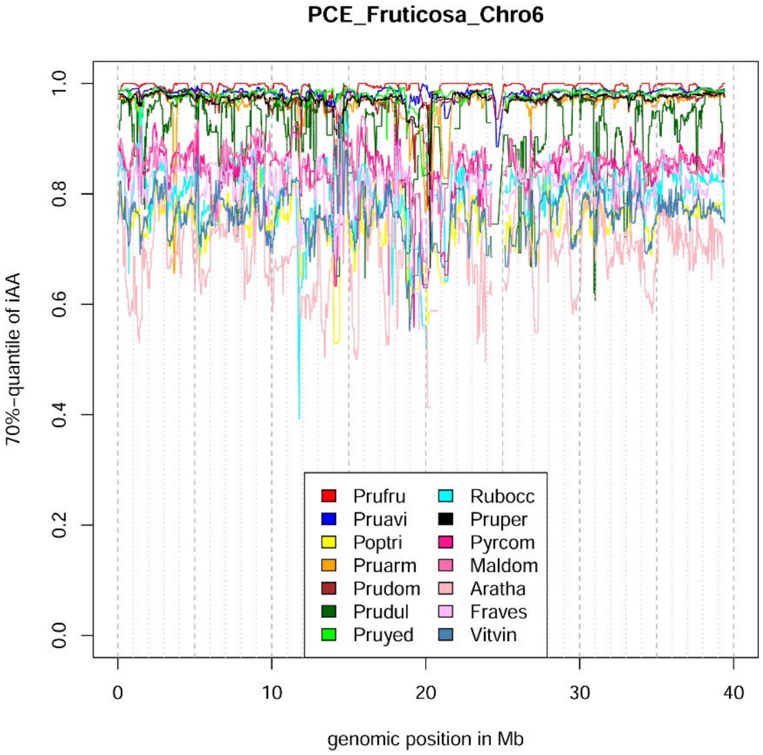
PCE_Fruticosa_Chro6
70%-quantile of iAA
0.0
0.2
0.4
0.6
0.8
1.0
0
10
20
30
40
genomic position in Mb
Prufru
Pruavi
Poptri
Pruarm
Prudom
Prudul
Pruyed
Rubocc
Pruper
Pyrcom
Maldom
Aratha
Fraves
Vitvin

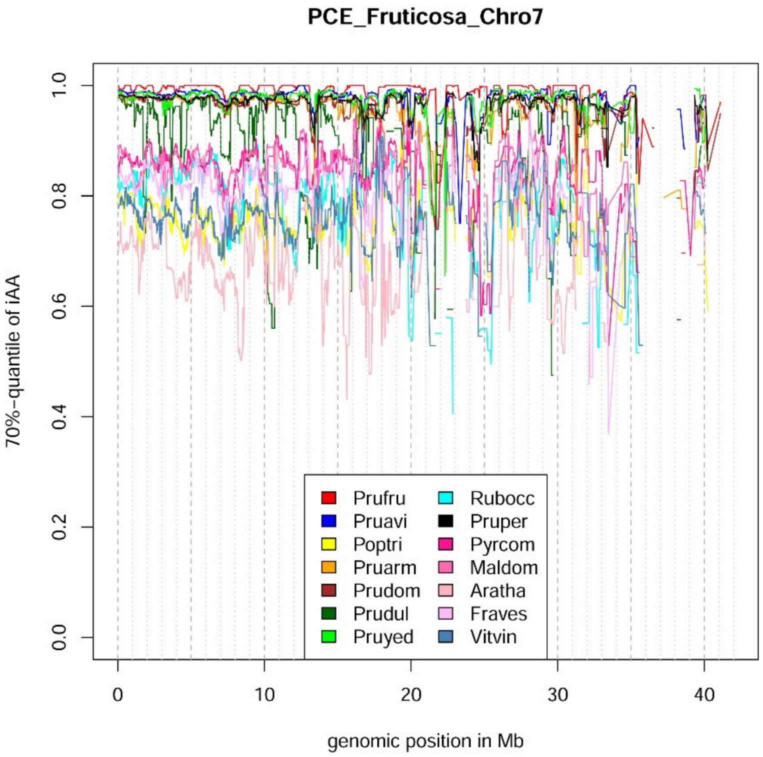

PCE_Fruticosa_Chro7
70%-quantile of iAA
genomic position in Mb
Prufru
Pruavi
Poptri
Pruarm
Prudom
Prudul
Pruyed
Rubocc
Pruper
Pyrcom
Maldom
Aratha
Fraves
Vitvin

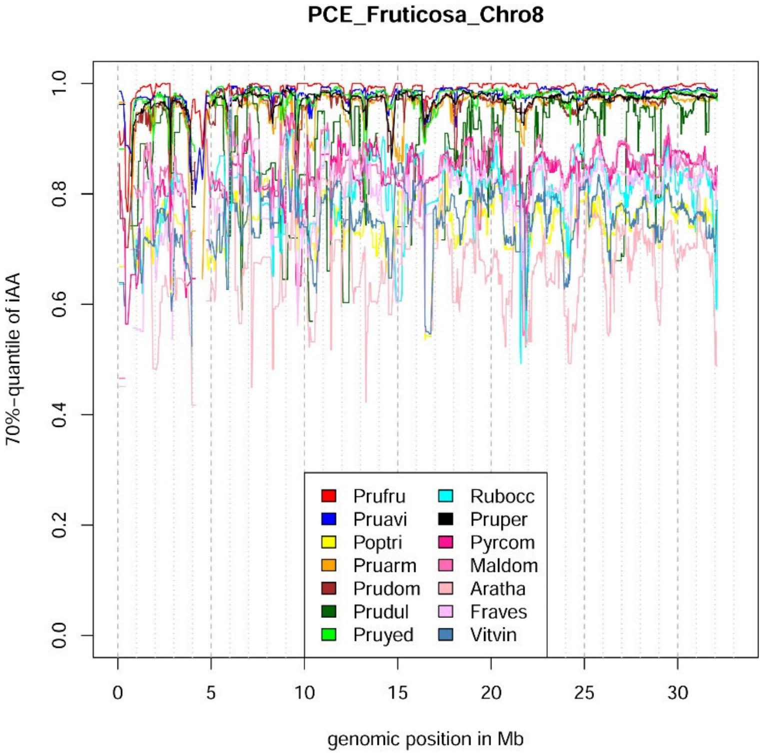

PCE_Fruticosa_Chro8
70%-quantile of iAA
genomic position in Mb
Prufru
Pruavi
Poptri
Pruarm
Prudom
Prudul
Pruyed
Rubocc
Pruper
Pyrcom
Maldom
Aratha
Fraves
Vitvin

Note S2 Calculation of %-covered bases obtained from RNAseq data.

The *Pce*S genome was divided into 250k windows. The percentage of covered bases using RNAseq data of *P. canescens* (SRX14816137), *P. serrulata* (SRX14816136), *P. mahaleb* (SRX14816140), *P. pensylvanica* (SRX14816144), *P. maackii* (SRX14816139), *P. subhirtella* (SRX14816145) was estimated for each window at a depth of 1. The results were plotted with a standard script using R.

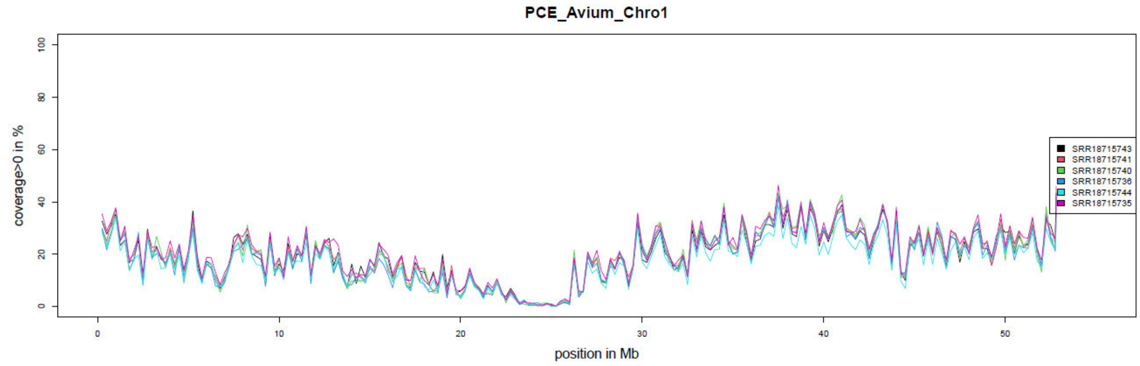

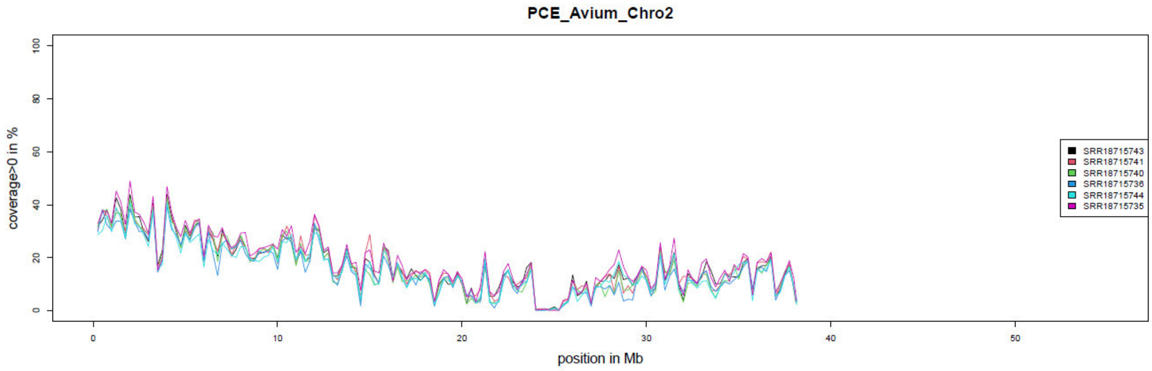

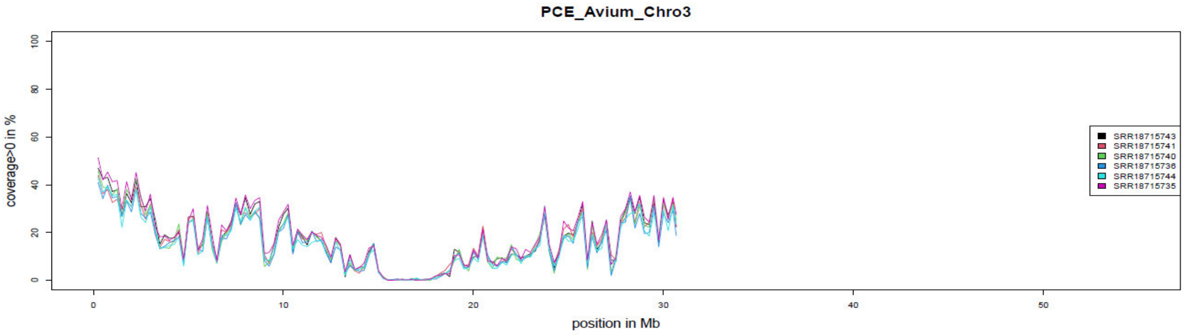

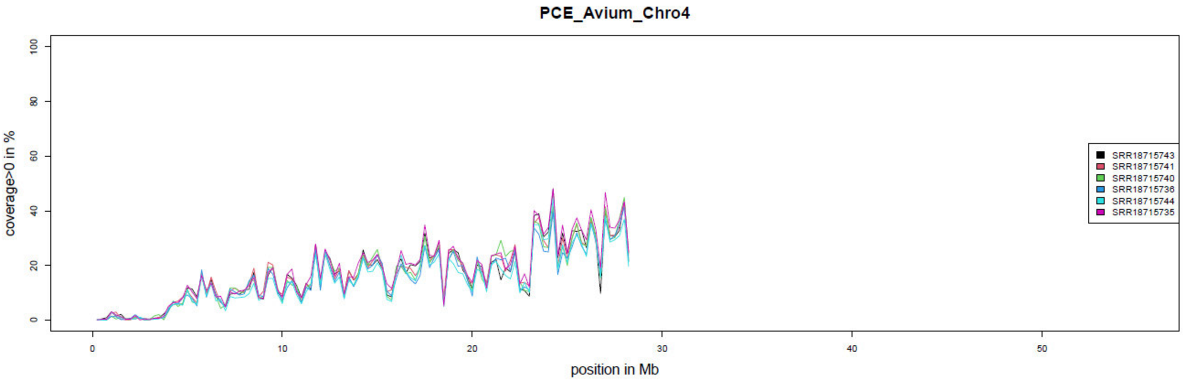

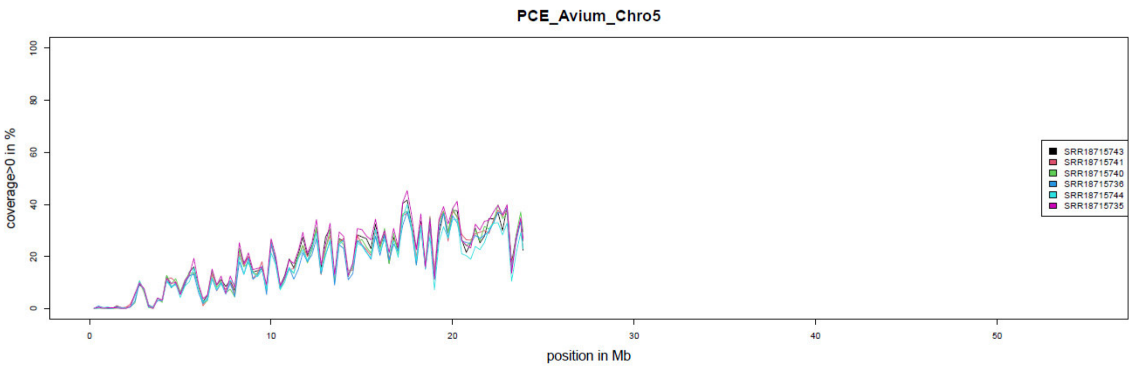
PCE_Avium_Chro5
coverage>0 in %
position in Mb
SRR18715743
SRR18715741
SRR18715740
SRR18715736
SRR18715744
SRR18715735

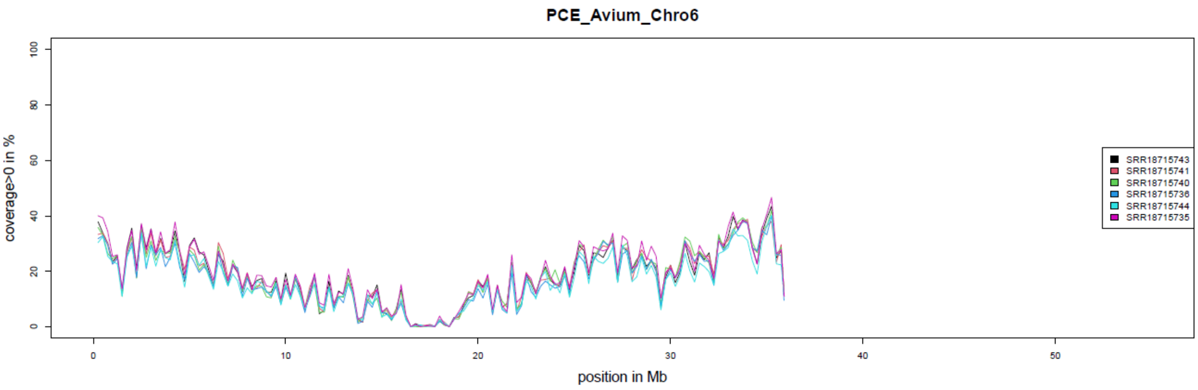
PCE_Avium_Chro6
coverage>0 in %
position in Mb
SRR18715743
SRR18715741
SRR18715740
SRR18715736
SRR18715744
SRR18715735

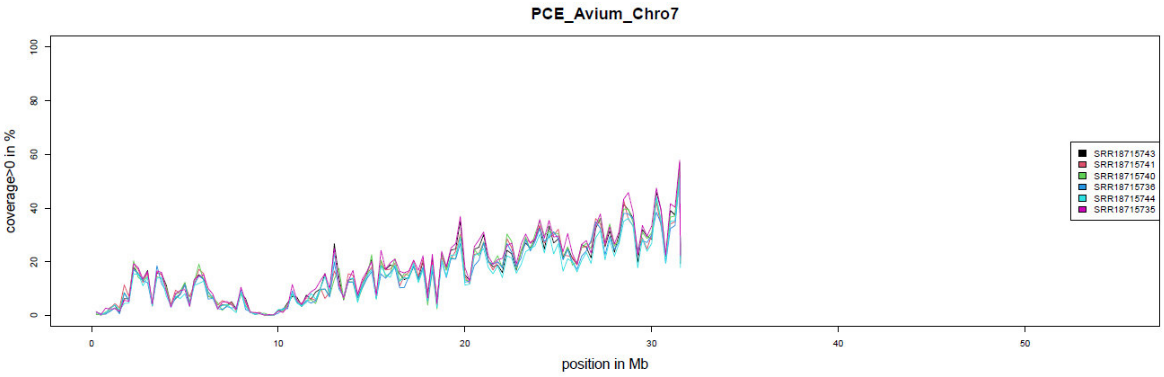
PCE_Avium_Chro7
coverage>0 in %
position in Mb
SRR18715743
SRR18715741
SRR18715740
SRR18715736
SRR18715744
SRR18715735

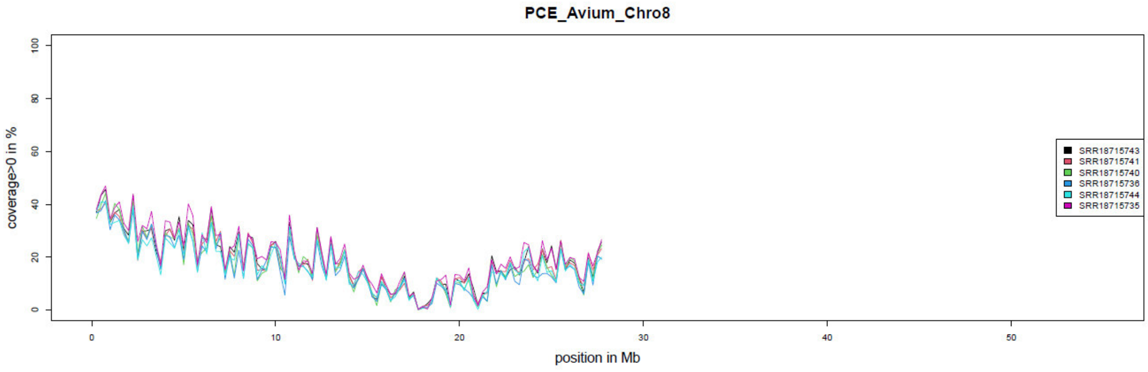
PCE_Avium_Chro8
coverage>0 in %
position in Mb
SRR18715743
SRR18715741
SRR18715740
SRR18715736
SRR18715744
SRR18715735

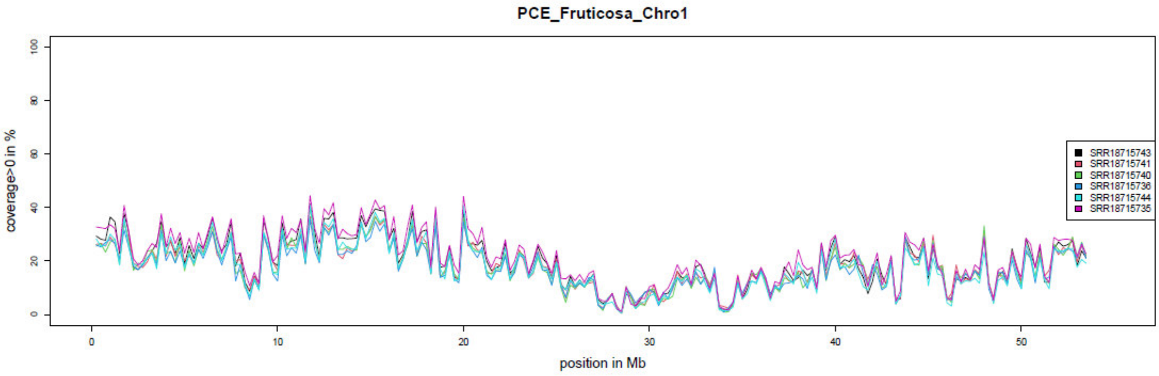
PCE_Fruticosa_Chro1
coverage>0 in %
position in Mb
SRR18715743
SRR18715741
SRR18715740
SRR18715736
SRR18715744
SRR18715735

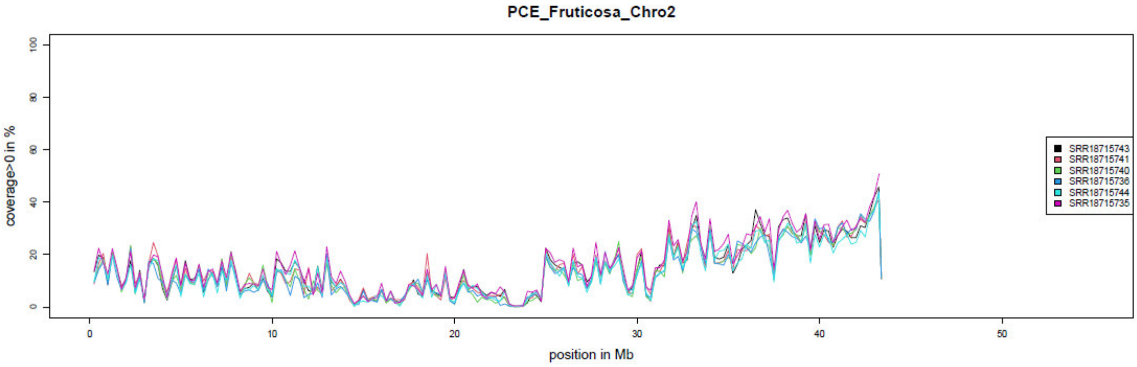
PCE_Fruticosa_Chro2
coverage>0 in %
position in Mb
SRR18715743
SRR18715741
SRR18715740
SRR18715736
SRR18715744
SRR18715735

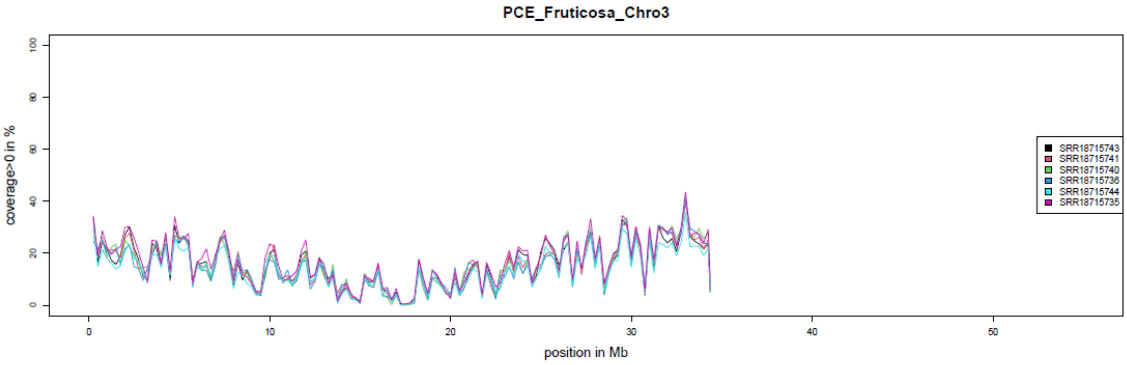
PCE_Fruticosa_Chro3
coverage>0 in %
position in Mb
SRR18715743
SRR18715741
SRR18715740
SRR18715736
SRR18715744
SRR18715735

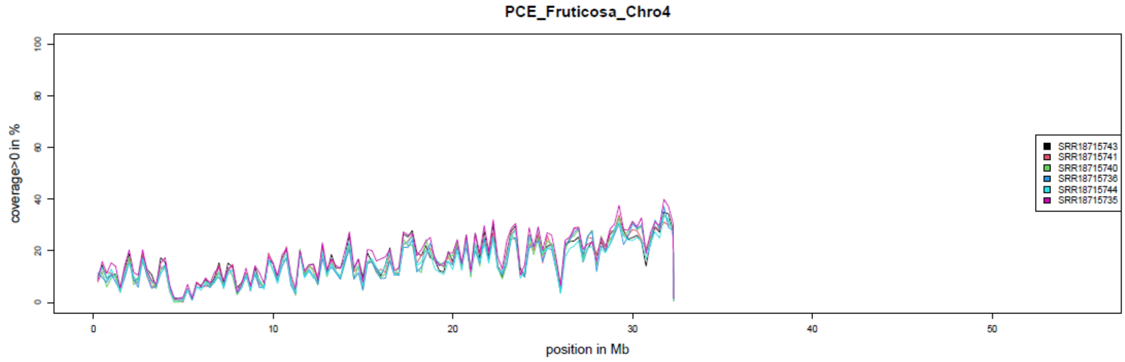
PCE_Fruticosa_Chro4
coverage>0 in %
position in Mb
SRR18715743
SRR18715741
SRR18715740
SRR18715736
SRR18715744
SRR18715735

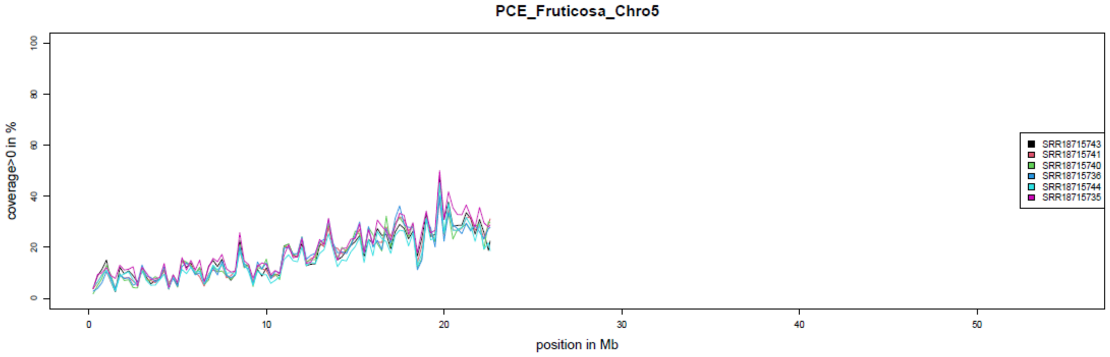
PCE_Fruticosa_Chro5
coverage>0 in %
position in Mb
SRR18715743
SRR18715741
SRR18715740
SRR18715736
SRR18715744
SRR18715735

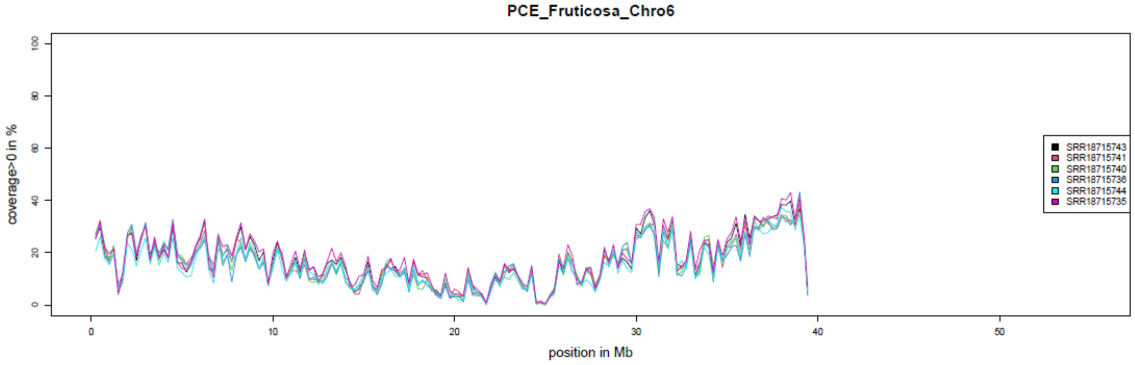
PCE_Fruticosa_Chro6
coverage>0 in %
position in Mb
SRR18715743
SRR18715741
SRR18715740
SRR18715736
SRR18715744
SRR18715735

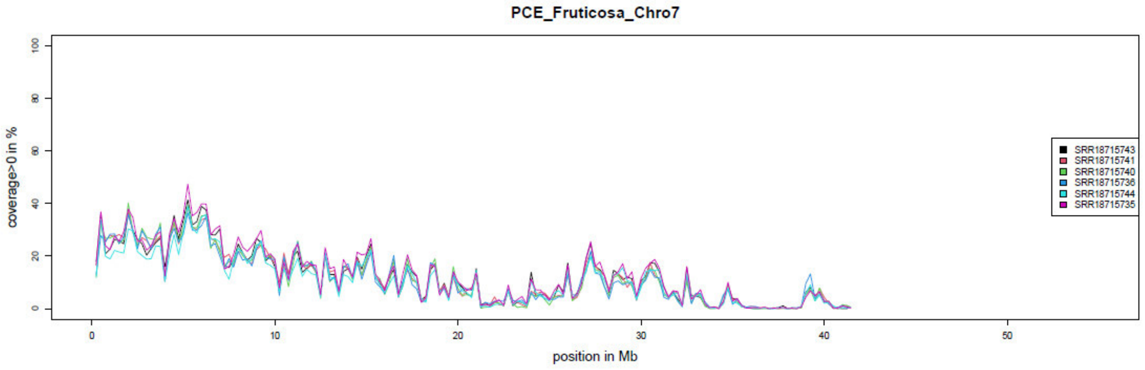
PCE_Fruticosa_Chro7
coverage>0 in %
position in Mb
SRR18715743
SRR18715741
SRR18715740
SRR18715736
SRR18715744
SRR18715735

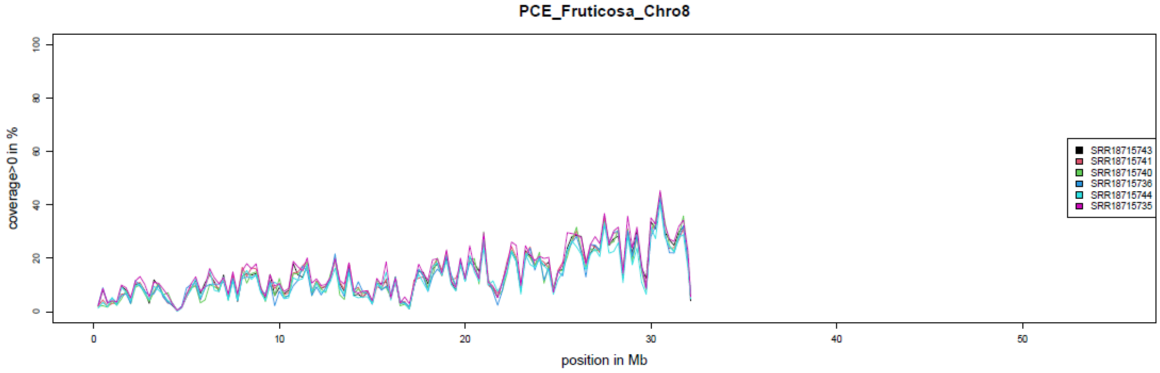
PCE_Fruticosa_Chro8
coverage>0 in %
position in Mb
SRR18715743
SRR18715741
SRR18715740
SRR18715736
SRR18715744
SRR18715735

Note S3 Access to the assembly hub for genome and annotation visualization at UCSC Genome Browser.

An assembly hub for genome and annotation visualization is permanently hosted at http://bioinf.uni-greifswald.de/hubs/pcer/hub.txt . To connect this assembly hub to the UCSC Genome Browser, go to https://genome.ucsc.edu, click on "My Data" -> "Track Hubs" -> select one of the mirrors-> "Connected Hubs" -> insert the link -> "Add Hub".

Note S4 Mummer plot between the genome sequence of *P. cerasus* 'Schattenmorelle' (this study) and 'Monmorency' (Goeckeritz et al. 2023). *Pce*$_S$_a – subgenome *P. avium* from 'Schattenmorelle', *Pce*$_S$_f – subgenome *P. fruticosa* from 'Schattenmorelle', *Pce*$_M$_B – subgenome *P. avium* from 'Montmorency', *Pce*$_M$_A – subgenome *P. fruticosa* from 'Montmorency' haplotype 1, *Pce*$_M$_A__ – subgenome *P. fruticosa* from 'Montmorency' haplotype 2.

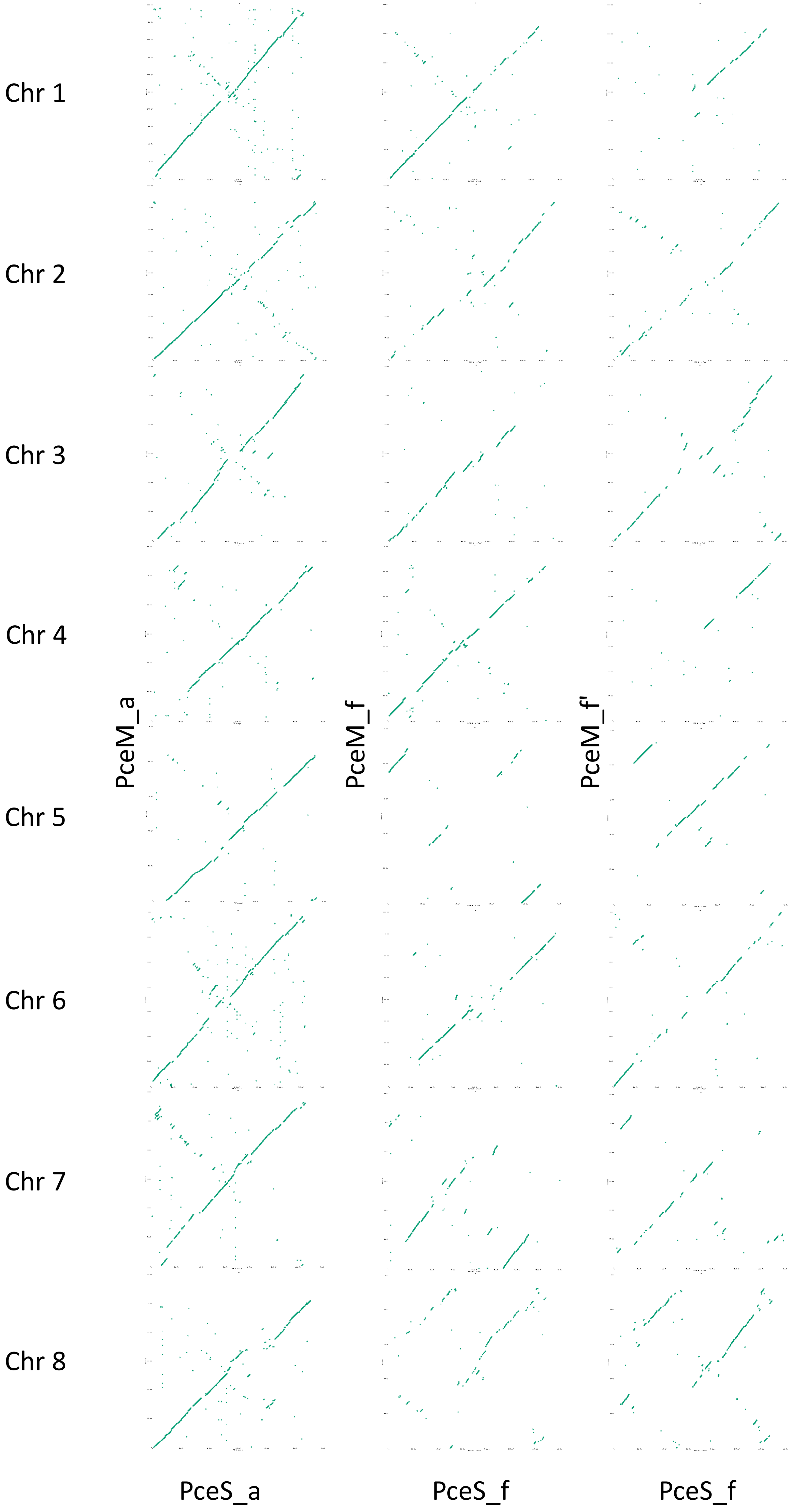
Chr 1
Chr 2
Chr 3
Chr 4
Chr 5
Chr 6
Chr 7
Chr 8
PceM_a
PceM_f
PceM_f'
PceS_a
PceS_f
PceS_f
