# Supplementary material for: The structure of the tetraploid sour cherry ‘Schattenmorelle’ (Prunus cerasus L.) genome reveals insights into its segmental allopolyploid nature
Source: Front Plant Sci. 2023 Dec 1;14:1284478. doi: 10.3389/fpls.2023.1284478 (PMC10722297; doi:10.3389/fpls.2023.1284478)
Supplement: Supplementary file 4 [file DataSheet_4.docx]

**References**

Kim, D., Paggi, J. M., Park, C., Bennett, C., & Salzberg, S. L. (2019). Graph-based genome alignment and genotyping with HISAT2 and HISAT-genotype. Nature biotechnology, 37(8), 907-915.

Li, H., & Durbin, R. (2009). Fast and accurate short read alignment with Burrows-Wheeler transform. Bioinformatics, 25(14), 1754–1760. https://doi.org/10.1093/bioinformatics/btp324

Li, H., & Durbin, R. (2010). Fast and accurate long-read alignment with Burrows–Wheeler transform. Bioinformatics, 26(5), 589–595. https://doi.org/10.1093/bioinformatics/btp698

Benson, G. (1999). Tandem repeats finder: a program to analyze DNA sequences.

Nucleic acids research, 27(2), 573-580

Bao, Z., & Eddy, S. R. (2002). Automated de novo identification of

repeat sequence families in sequenced genomes. Genome research, 12(8),

1269-1276.

Gremme, G., Steinbiss, S., & Kurtz, S. (2013). GenomeTools: a

comprehensive software library for efficient processing of structured genome

annotations. IEEE/ACM transactions on computational biology and bioinformatics,

10(3), 645-656.

Ou, S., & Jiang, N. (2018). LTR_retriever: a highly accurate and sensitive

program for identification of long terminal repeat retrotransposons.

Plant physiology, 176(2), 1410-1422.

Wheeler, T. J. (2009, September). Large-scale neighbor-joining with NINJA.

In International Workshop on Algorithms in Bioinformatics (pp. 375-389).

Springer, Berlin, Heidelberg.

Katoh, K., & Standley, D. M. (2013). MAFFT multiple sequence alignment

software version 7: improvements in performance and usability. Molecular

biology and evolution, 30(4), 772-780.

Fu, L., Niu, B., Zhu, Z., Wu, S., & Li, W. (2012). CD-HIT: accelerated for

clustering the next-generation sequencing data. Bioinformatics, 28(23),

3150-3152.

Li, H. (2018). Minimap2: pairwise alignment for nucleotide sequences.

Bioinformatics, 34(18), 3094-3100.

Li, H., Handsaker, B., Wysoker, A., Fennell, T., Ruan, J., Homer, N., ... &

Durbin, R. (2009). The sequence alignment/map format and SAMtools.

Bioinformatics, 25(16), 2078-2079.

Hoff, K. J., Lange, S., Lomsadze, A., Borodovsky, M., & Stanke, M. (2016).

BRAKER1: unsupervised RNA-Seq-based genome annotation with GeneMark-ET and

AUGUSTUS. Bioinformatics, 32(5), 767-769.

Hoff, K. J., Lomsadze, A., Borodovsky, M., & Stanke, M. (2019). Whole-genome

annotation with BRAKER. In Gene Prediction (pp. 65-95). Humana, New York, NY

J. Keilwagen, F. Hartung, J. Grau, GeMoMa: Homology-based gene prediction utilizing intron position conservation and RNA-seq data, in: Gene Prediction, Springer, 2019, pp. 161–177.

M. Tillich, P. Lehwark, T. Pellizzer, E.S. Ulbricht-Jones, A. Fischer, R. Bock, S. Greiner, GeSeq–versatile and accurate annotation of organelle genomes, Nucleic acids research 45 (2017) W6-W11.

M. Yan, X. Zhang, X. Zhao, Z. Yuan, The complete mitochondrial genome sequence of sweet cherry (*Prunus* *avium* cv.‘summit’), Mitochondrial DNA Part B 4 (2019) 1996–1997.

Katoh, K., & Standley, D. M. (2013). MAFFT Multiple Sequence Alignment Software Version 7: Improvements in Performance and Usability. *Molecular Biology and Evolution*, *30*(4), 772–780. <https://doi.org/10.1093/molbev/mst010>

Tamura K., Battistuzzi FU, Billing-Ross P, Murillo O, Filipski A, and Kumar S. (**2012**). Estimating Divergence Times in Large Molecular Phylogenies. *Proceedings of the National Academy of Sciences* **109**:19333-19338.

Tamura K., Qiqing T., and Kumar S. (**2018**). Theoretical Foundation of the RelTime Method for Estimating Divergence Times from Variable Evolutionary Rates. *Molecular Biology and Evolution***35**: 1770-1782.

Tao Q., Tamura K,. Mello B., and Kumar S. (**2020**) Reliable Confidence Intervals for RelTime Estimates of Evolutionary Divergence Times. *Molecular Biology and Evolution, 37(1): 280-290*

Wöhner, T. W., Emeriewen, O. F., Wittenberg, A. H., Schneiders, H., Vrijenhoek, I., Halász, J., ... & Flachowsky, H. (2021a). The draft chromosome-level genome assembly of tetraploid ground cherry (Prunus fruticosa Pall.) from long reads. *Genomics*, *113*(6), 4173-4183.

T.W. Wöhner, O.F. Emeriewen, A.H.J. Wittenberg, H. Schneiders, I. Vrijenhoek, J. Halász, K. Hrotkó, K.J. Hoff, L. Gabriel, J. Keilwagen, T. Berner, M. Schuster, A. Peil, J. Wünsche, S. Kropop, H. Flachowsky (2021b). Supporting Materials for - The Draft Chromosome-level Genome Assembly of Tetraploid Ground Cherry (*Prunus fruticosa* Pall.) from Long Reads. <https://www.openagrar.de/receive/openagrar_mods_00070329> (2021) (accessed 1 June 2021)

Jones D.T., Taylor W.R., and Thornton J.M. (1992). The rapid generation of mutation data matrices from protein sequences. Computer Applications in the Biosciences 8: 275-282.

Bolger AM, Lohse M, Usadel B. Trimmomatic: a flexible trimmer for Illumina sequence data. Bioinformatics 2014;30(15):2114–20. <https://doi.org/10.1093/bioinformatics/btu170>.

Arang Rhie (2020). Meryl. In GitHub repository. GitHub. https://github.com/marbl/meryl.
