# Supplementary material for: The structure of the tetraploid sour cherry ‘Schattenmorelle’ (Prunus cerasus L.) genome reveals insights into its segmental allopolyploid nature
Source: Front Plant Sci. 2023 Dec 1;14:1284478. doi: 10.3389/fpls.2023.1284478 (PMC10722297; doi:10.3389/fpls.2023.1284478)
Supplement: Supplementary file 5 [file DataSheet_5.docx]

Additional datasets from this study can be found in the following repository:

Wöhner, T., Emeriewen, O. F.,Wittenberg, A. H. J., Nijbroek, K.,Wang, R. P., Blom, Q5

E.-J., et al. (2023). Data set: the structure of the tetraploid sour cherry ‘Schattenmorelle’

(Prunus cerasus L.) genome reveals insights into its segmental allopolyploid nature.

OpenAgrar Repository. doi: 10.5073/20230324-105730-0
